# Associations of Exposure to Common Plasticizers and Organophosphate Pesticides during Pregnancy and in Childhood with Cognitive Performance in Adolescents: A Population-Based Study

**DOI:** 10.1021/acs.est.5c18986

**Published:** 2026-06-12

**Authors:** Yuchan Mou, Hanan El Marroun, Mengling Liu, Arash Derakhshan, Mònica Guxens, Vincent W. Jaddoe, Tonya White, Kurunthachalam Kannan, Suzanne Spaan, Anjoeka Pronk, Leonardo Trasande, Henning Tiemeier, Akhgar Ghassabian

**Affiliations:** † Department of Child and Adolescent Psychiatry/Psychology, Erasmus MC, University Medical Center Rotterdam, Rotterdam 3015 CN, The Netherlands; ‡ The Generation R Study Group, Erasmus MC, University Medical Center Rotterdam, Rotterdam 3015 CN, The Netherlands; § Department of Psychology, Education and Child Studies, Erasmus School of Social and Behavioural Sciences, Rotterdam 3062 PA, The Netherlands; ∥ Department of Population Health, 12296 NYU Grossman School of Medicine, New York, New York 10016, United States; ⊥ Department of Internal Medicine, Erasmus University Medical Center, Rotterdam 3015 CN, The Netherlands; # Academic Center for Thyroid Diseases, Erasmus University Medical Center, Rotterdam 3015 CN, The Netherlands; ∇ Institute for Risk Assessment Sciences (IRAS), Utrecht University, Utrecht 3584 CM, The Netherlands; ○ ISGlobal, Barcelona 08003, Spain; ◆ ICREA, Barcelona 08010, Spain; ¶ Universitat Pompeu Fabra, Barcelona 08002, Spain; ^††^ Spanish Consortium for Research on Epidemiology and Public Health (CIBERESP), Instituto de Salud Carlos III, Madrid 28029, Spain; ^‡‡^ Department of Pediatrics, Erasmus MC, University Medical Center Rotterdam, Rotterdam 3015 CN, The Netherlands; ^§§^ Section on Social and Cognitive Developmental Neuroscience, 25944 National Institute of Mental Health, Bethesda, Maryland 20814, United States; ^∥∥^ Department of Pediatrics, NYU Grossman School of Medicine, New York, New York 10016, United States; ^⊥⊥^ Wadsworth Center, 1094 New York State Department of Health, Albany, New York 12208, United States; ^##^ Department Risk Analysis for Prevention, Innovation and Development (RAPID), TNO, 34, Utrecht 3584 CB, The Netherlands; ^∇∇^ New York University Wagner Graduate School of Public Service, New York, New York 10012, United States; ^○○^ Department of Social and Behavioral Sciences, Harvard TH Chan School of Public Health, Boston, Massachusetts 02115, United States

**Keywords:** endocrine-disrupting chemicals, nonpersistent chemicals, mixture analysis, epidemiology, neurodevelopment

## Abstract

Individuals are exposed to chemicals in daily life. Yet, few studies have examined the long-lasting joint effect of prenatal and childhood exposure to endocrine-disrupting chemical (EDC) on cognitive performance. We analyzed data from mother–child pairs from the Generation R birth cohort (The Netherlands, 2002–2006) with urinary levels of ten phthalate metabolites, bisphenol A, and five nonspecific organophosphate pesticides metabolites three times during pregnancy (*n* = 565) and at 5 years of age (*n* = 539). Child cognitive performance was assessed using the vocabulary, matrix reasoning, digit span, and coding subtests of the Wechsler Intelligence Scale at 13 years. Using hierarchical Bayesian kernel machine regression, we found that prenatal EDC mixture level at 75th percentile versus the median was associated with 0.33 decrease (95% credible interval: −0.60, −0.06) in verbal comprehension and with 0.26 decrease (−0.51, −0.02) in matrix reasoning scores, with di­(2-ethyhexyl) phthalate and dibutyl phthalates as primary contributing chemicals to the mixture effect for matrix reasoning. Higher childhood levels of EDC mixture were associated with higher verbal scores, in contrast to the inverse associations observed for prenatal exposure, although this finding should be interpreted with caution due to potential exposure misclassification, selection bias, and residual confounding. Overall, our findings suggest that prenatal exposure to a mixture of plasticizers and pesticides may have a long-lasting adverse effect on offspring’s cognition.

## Graphical abstract

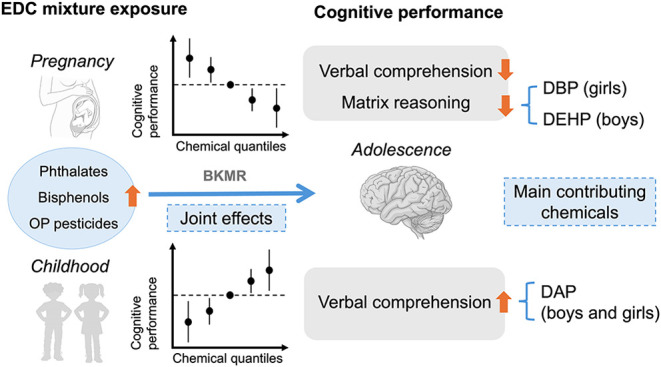

## Introduction

Brain development is sensitive to hormonal influences, particularly during periods of rapid growth, such as structural differentiation and functional maturation that occurs during the prenatal period and childhood.^1,2^ Exposure to endocrine-disrupting chemicals (EDCs), a group of synthetic compounds that have been extensively used in industrial and consumer products for decades,^3^ can potentially interfere with important brain processes because of the hormonal disrupting properties and developmental neurotoxicity of these chemicals.^4–6^ Recent reviews of epidemiological studies have provided generally consistent evidence for associations of exposure to several groups of EDCs, including common plasticizers (e.g., phthalates and bisphenols)^7–9^ and organophosphate (OP) pesticides,^10^ with adverse cognitive performance in the general population. All of these chemicals are abundantly detected in humans, mainly through food and commonly used consumer products.^11–13^

People are exposed to multiple chemicals simultaneously, which can result in additive, synergistic, or antagonistic interactions among chemicals. Investigating chemical mixtures facilitate capturing such complex effects and pinpointing driving chemicals in the mixture.^14^ Given that the prenatal period presents a critical window of neurodevelopment characterized by rapid neurogenesis, neuronal migration, and differentiation, processes that are largely dependent on endocrine hormone signaling and may be particularly vulnerable to disruption by environmental chemicals,^4^ prior research has mainly focused on prenatal chemical exposure. Recent studies suggest that prenatal exposure to chemical mixtures, including phenols, phthalates, and OP pesticides, was inversely associated with children’s intelligent quotient (IQ),^15–18^ with some studies showing sex differences in the associations.^15,19^ For instance, a study using data from the Generation R Study found that higher prenatal exposure to the chemical mixture, including phthalates, bisphenol, and OP pesticides, was associated with lower nonverbal IQ in six-year-old children, mainly driven by a mixture of phthalates.^16^ Another study explored a mixture of five groups of EDCs and found negative associations of mixture with full-scale IQ of children at 7 years of age, with more pronounced associations among boys. Bisphenol F was found to be the strongest contributor to the mixture effect.^15^ It remains unclear whether the effect of prenatal exposure to chemical mixtures persists into adolescence. Beyond prenatal window, brain development continues throughout childhood with ongoing synaptogenesis, myelination, and synaptic pruning, that support the maturation of cognitive functions such as language and reasoning, making childhood another relevant exposure window.^4^ Moreover, although cross-sectional negative associations have been found between childhood metabolite levels of phthalates and IQ scores,^20^ longitudinal data on childhood EDC exposure and cognitive development are limited.

In this study, we examined the associations of prenatal and early childhood exposures to a mixture of phthalates, bisphenols, and OP pesticides with cognitive performance in adolescents. From the overall mixture effect, we identified the major components that contributed to the observed associations. Furthermore, we explored potential sex differences in these associations.

## Methods

### Study Design and Population

This study was embedded in the Generation R Study, an ongoing population-based prospective cohort from early fetal life onward in Rotterdam, The Netherlands.^21^ The study was approved by the Medical Ethics Committee of Erasmus Medical Center in Rotterdam, The Netherlands (MEC 198.782/2001/31, MEC 217.595/2002/202, MEC-2007-413, MEC-2015-749). Written informed consent was obtained from all participating children and their parents.

Of 8879 eligible women with a delivery date between April 2002 to January 2006 and enrolled in pregnancy, 2089 women provided up to three urine samples during pregnancy. Among them, urine samples of 1405 women who had singleton pregnancies with postnatal consent and whose children participated in follow-up assessment at age 6 were analyzed for phthalate metabolites and bisphenols.^22^ For OP pesticide exposure assessment, a subsample of 800 mother–child pairs who had urine samples in pregnancy and follow-up data of children were selected.^23,24^ The selection of child urine samples for chemical measurements was based on the availability of a chemical assessment in their mothers. In total, 775 mothers and 742 children at age 6 had information on all chemical assays, from whom 565 and 539 had data on child cognitive assessment at 13 years of age, respectively (Figure S1).

### Measurements

#### EDC Exposure Assessment

Pregnant women provided spot urine samples at three time points during gestation: early (median = 12.9 weeks of gestation, range = 6.5–17.9 weeks), mid (median = 20.4 weeks of gestation, range = 18.1–24.9 weeks), and late pregnancy (median = 30.2 weeks of gestation, range = 27.4–34.5 weeks). Children provided a spot urine sample at the age of 6 years (median = 6.0, range = 5.0–6.9). These samples were analyzed for bisphenols, phthalate metabolites, and nonspecific dialkylphosphate (DAP) metabolites of OP pesticides. Detailed information on urine sample collection and the quantification of EDCs is described elsewhere.^22,24,25^ Briefly, the quantitative detection of eight bisphenols was performed by liquid–liquid extraction following enzymatic deconjugation and high-performance liquid chromatography–electrospray ionization–tandem mass spectrometry (HPLC-ESI-MS/MS) at Wadsworth laboratory, New York. Quantitative detection of 18 phthalate metabolites was achieved using solid-phase extraction following enzymatic deconjugation of the glucuronidated phthalate monoesters and HPLC-ESI-MS/MS at Wadsworth laboratory. Limits of detection (LOD) for bisphenols and phthalate metabolites were in the range of 0.008–1.11 ng/mL. Quality control procedure included the use of procedural blanks to monitor background contamination, matrix spike samples to assess recovery and analytical performance, calibration check standards, and methanol injections after each batch samples to monitor instrumental drift and carry-over between samples. Six DAP metabolites of OP pesticides were measured using gas chromatography coupled with tandem mass spectrometry (GC-MS/MS) at Institut National de Santé Publique in Quebec (INSPQ), Canada, with LODs in the range of 0.06–0.50 ng/mL. Quality control procedure included the assessment of interday precision based on reference materials and the evaluation of reliability through interlaboratory comparisons in a subset of participants. The coefficients of variation were <15% for phthalate and bisphenol metabolites, and 4.1–9.1% for DAP metabolites.^22,24^

We included chemicals with detection frequencies higher than 70%, resulting in 16 chemicals during pregnancy and 14 chemicals at the age 6 years in the further analyses. During pregnancy, included phthalate metabolites were monobenzyl phthalate (mBzP), mono­(2-ethyl-5-carboxypentyl) phthalate (mECPP), mono­(2-ethyl-5-hydroxyhexyl) phthalate (mEHHP), mono­(2-ethyl-5-oxohexyl) phthalate (mEOHP), mono­(2-carboxymethyl)­hexyl phthalate (mCMHP), monobutyl phthalate (mBP), monoisobutyl phthalate (mIBP), monoethyl phthalate (mEP), monomethyl phthalate (mMP), and mono­(3-carboxypropyl) phthalate (mCPP). For bisphenols, bisphenol A (BPA) was included. For OP pesticides, diethyl phosphate (DEP), diethylthiophosphate (DETP), dimethyldithiophosphate (DMDTP), dimethylphosphate (DMP), and dimethylthiophosphate (DMTP) were included. At the age 6 years, the same set of chemical metabolites were included except for mBzP and DMDTP, which had detection frequencies at 59% and 27%, respectively.

To account for urinary dilution, creatinine concentration was quantified together with chemical exposure measurements and used to adjust chemical concentration using the Boeniger method with the formula $E_{\mathrm{cr}}=E_{o}\frac{{\mathrm{Cr}}_{\mathrm{median}}}{{\mathrm{Cr}}_{o}}$ ,^26^ where *E*_cr_ is the creatinine-standardized chemical concentration, *E*_o_ is the observed chemical concentration, Cr_median_ is the median of creatinine concentrations in the study sample, Cr_o_ is the observed urinary creatinine concentration. Concentrations below the LOD were imputed by the LOD of that compound divided by √2.

#### Cognitive Performance in Adolescence

Cognitive performance was assessed using four subtests of the Wechsler Intelligence Scale for Children-Fifth Edition (WISC-V) when children were 13 years (median = 13.5, 95% range = 13.1–4.7). The WISC-V is a validated tool for assessments of cognitive performance in 6- to 16-year-olds. In Dutch population, it has demonstrated high inter-rater agreement (>0.98) for verbal comprehension subtests, which involve more subjective scoring, suggesting a high degree of consistency in the scores.^27^ In the Generation R Study, all four subtests were administered by research assistants trained by a clinical neuropsychologist. Research assistants first scored the same subset of tests and proceeded only if reliability reached 0.9. Uncertain responses were resolved by reviewing task recordings and scoring issues were discussed in monthly meetings. The four subtests included vocabulary, matrix reasoning, digit span and coding, corresponding to the measurement of verbal comprehension, fluid reasoning, working memory, and processing speed. Further details of selection of subtests and administration are described elsewhere.^28,29^ Raw subsets scores were converted to age-standardized T-scores (ranging from 1 to 19) based on Dutch norm scores, with higher scores indicating better performance. The standardized subtest scores were approximately normally distributed.

### Covariates

Child sex and date of birth were collected from medical records completed by obstetricians and community midwives. Information on household income, maternal age, national origin, education attainment, and smoking habits was collected through self-reported questionnaires during pregnancy. Household income was categorized into <€1200, €1200–€2200, and >€2200 per month. Mothers’ highest education attainment was dichotomized into low (from no education up to lower vocational training) and high (higher vocational training/university). Smoking during pregnancy was categorized as never, until pregnancy was known, and continued. Folic acid supplement use was grouped into no use during embryogenesis, started the first 10 weeks and started periconceptional. Maternal prepregnancy weight was obtained by self-report questionnaires. Maternal nonverbal IQ was assessed using the computerized Raven’s Advanced Progressive Matrices Test Set I when mothers visited the research center when their children were 6 years old.^30^ Maternal verbal IQ was assessed using the vocabulary test of Wechsler Adult Intelligence Scale when mother and children participated the follow-up at age 13.^31^ Child migration background was determined based on the country of origin of their parents and grouped as being from the Netherlands and from outside the Netherlands. Height and weight were measured by trained staff at the research center when children were 6 years old (range = 4.8–9.1 years). Child’s sex- and age-specific BMI (kg/m^2^) standard deviation (SD) scores were calculated using Dutch reference growth curves.

### Statistical Analysis

The characteristics of study population was described as mean and SD for continuous variables with normal distributions, median, and interquartile range (IQR) for continuous variables with skewed distributions, or percentages of categorical variables. Distributions of prenatal and childhood creatinine-adjusted urinary concentrations of phthalate metabolites, bisphenols, and DAP metabolites were described as median and IQR, and differences between girls and boys were evaluated using the Mann–Whitney *U* test. Correlations among chemical concentrations were presented using Spearman’s correlation.

Prior to the analyses, we imputed missing values of covariates in the sample of 8879 eligible mother–child pairs, using multiple imputation by chained equations under the assumption that data were missing at random, generating 30 imputed data sets with 50 iterations using the *mice* package in R.^32^ Subsequent analyses were restricted to the analytical samples of 565 and 539 mother–child pairs with complete data on chemical assessment and cognitive performance. In these analytical samples, the percentages of missing values in covariates ranged from 0.4% (parity) to 19.7% (folic acid supplementation) (Table S1). All chemicals were log 2 transformed and standardized. To reduce the influence of outliers, values above the 99th percentile were winsorized with the 99th percentile value prior to inclusion in the statistical models.^33^ Given nonpersistent properties of included chemicals, we averaged prenatal chemical concentration across pregnancy under the assumption of relatively consistent exposure during pregnancy. Intraclass correlation coefficients for the three measurements across pregnancy, assessed using two-way mixed effects models with multiple measurements and absolute agreement, ranged from 0.09 to 0.64 (Table S4).

We conducted Bayesian kernel machine regression (BKMR) to examine the association between the EDC mixture and the four subtests of cognitive performance allowing for potential nonlinear and synergistic effects among chemicals in the EDC mixture. We also examined sex-specific associations using stratified analyses by sex, fitting BKMR models for boys and girls separately. This approach allows us to flexibly model the joint effect of the EDC mixture using a kernel function to characterize the high-dimensional exposure–response function.^34^ To improve estimation precision and minimize noise, we used a two-step BKMR modeling approach. In the first step, we applied BKMR with hierarchical variable selection (hBKMR) using Gaussian kernel to the full set of individual chemicals. Chemicals were grouped based on their parent compounds and the model simultaneously selected important variable among groups and among individual chemicals.^35^ See Supporting Table S2 for the full list of chemicals and groups. We considered group-specific posterior inclusion probabilities (PIP) ≥ 0.2 as suggestive evidence to keep candidate chemical groups in subsequent analyses. In the second step, we ran hBKMR restricted to the chemicals within the selected groups to estimate associations with cognitive performance. In these models, we estimated the overall effects of the mixture by assessing the average difference in cognitive performance scores associated with simultaneously increasing quantiles of all chemicals, compared to when all chemicals are fixed at their median. Overall mixture associations were estimated by posterior mean estimates and 95% credible intervals (CrI). We estimated group-specific and conditional PIPs to assess the relative importance of each group and each chemical within the group in relation to the outcome. We used a commonly used PIP ≥ 0.5 as the cutoff^36,37^ to flag chemical groups or individual chemicals with important contribution to the overall mixture associations. Then, we examined marginal dose–response associations between each chemical and the outcome by visualizing the exposure–response functions for increasing concentrations of each chemical, when all other chemicals are fixed at their median. Lastly, we summarized the contribution of individual chemicals and potential interactions by visualizing the effect of an increase from the 25th to 75th percentile (i.e., interquartile range, IQR) in a single chemical on cognitive performance scores when all other chemicals are fixed at either the 25th, 50th, or 75th percentile.

For all hBKMR analysis, we fitted separate models for each of the 30 imputed data sets, using 6500 iterations with eight Markov chains per model. Due to the computational demands of BKMR, we randomly selected 10 hBKMR fits to derive final estimates using Rubin’s method. Posterior distributions of the model parameters were generated using Markov Chain Monte Carlo, and convergence and chain stability were evaluated using trace plots and Gelman’s r-hat statistics (all r-hat <1.05). After confirming adequate diagnostics, we combined the eight chains into a single chain of 50,000 iterations, discarded the first 30,000 iterations as burn-in, and kept every fifth iteration for inference. In addition, we performed single-chemical associations analysis of individual chemical metabolites in relation to adolescent cognitive performance.

We used directed acyclic graphs (DAG) to guide the selection of confounders reported in previous studies for the associations between prenatal EDC mixture exposure (Figure S2A) and childhood EDC mixture exposure (Figure S2B) with adolescents’ cognitive performance.^16,22,38–40^ All models for prenatal and childhood chemical exposure were adjusted for child age at the cognitive assessment, child sex, child migration background, maternal age, prepregnancy BMI, parity, educational level, family income, maternal smoking during pregnancy, and maternal folic acid supplement use. In addition, models of verbal subtest were adjusted for maternal verbal IQ, and models of coding, digit span, matrix reasoning subtests were adjusted for maternal nonverbal IQ. Models of childhood chemical exposure was additionally adjusted for child BMI at age 5.

In a sensitivity analysis, we examined whether prenatal chemical exposure acted as a confounder in the overall association of childhood chemical mixture exposure with IQ by additionally adjusting for prenatal molar concentrations of phthalic acid, BPA, and the weighted molar sum concentration of DAP metabolites in childhood exposure models.

We performed the BKMR analysis using the *bkmr, bkmrhat*, and *causalbkmr* packages in R version 4.4.2 and all other analysis in R 4.4.1.

## Results

The characteristics of study population are presented in Table 1. About 60% of the mothers had high educational levels, family income >€2200, and a Dutch national origin. The average verbal comprehension score for 14-year-old children was 9.8 (SD = 2.9), the coding score was 12.8 (SD = 3.2), the digit span score was 9.5 (SD = 2.7), and the matrix reasoning score was 9.2 (SD = 2.5) out of a total possible score of 19. Comparison between the characteristics of Generation R participants recruited in pregnancy, respondents with chemicals measurements, and respondents in the analytical sample (available exposure and outcome measures) showed that mothers and children who were included in analyses more often have a Dutch national origin, higher socioeconomic status, less smoking during pregnancy, more folic acid supplementation use started periconceptional, lower BMI at the age of 5 years, and higher cognitive performance scores (Table S3) than the other two groups.

**Table 1:**
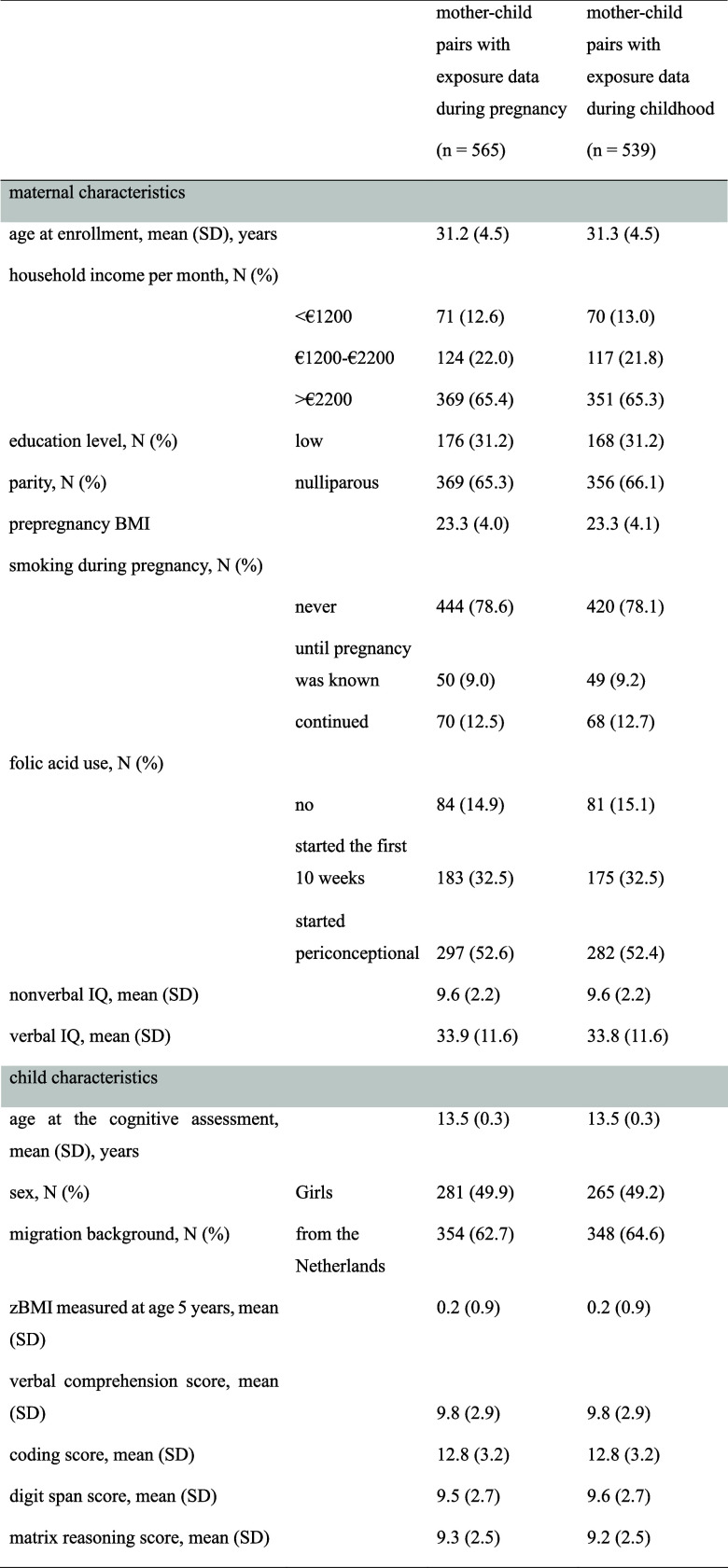
Characteristics of the Study Population^a^

a The results of this study are shown in Figure 1. Values are mean (standard deviation, SD) for continuous variables with a normal distribution or valid numbers (%) for categorical variables. Missing data of covariates were imputed with multiple imputation (*m* = 30 imputations).

Median (IQR) of prenatal (averaged across pregnancy) and childhood (age 6) urinary concentrations of phthalate and DAP metabolites and bisphenol A are reported in Table 2. There were no significant differences in prenatal and childhood chemical concentrations by child sex (all *p*-value >0.05), except prenatal DEP (DAP metabolite, *p* = 0.005). Further details on descriptive statistics of chemicals are shown in Tables S4 and S5. Moderate to high correlations were observed among metabolites originating from the same parent phthalate compound (ranging from 0.58 to 0.97) or DAPs (ranging from 0.24 to 0.73), both during pregnancy and in childhood (Figures S3 and S4). Correlations between prenatal and childhood exposure chemicals were overall null to low (|*r*| ≤ 0.23; see Figure S5).

**Table 2:** Distributions of Prenatal and Childhood Urinary Phthalates, Bisphenols, and Organophosphate Pesticides Metabolite Concentrations^a^,^b^

		averaged pregnancy			in childhood		
metabolites unit (ng/mL)	parent compounds	total	girls^c^	boys^c^	total	girls^c^	boys^c^
mBzP	BBzP	4.89 (2.94, 9.70)	5.11 (2.79, 9.74)	4.66 (3.04, 9.51)	N/A	N/A	N/A
mCMHP	DEHP	8.39 (6.05, 12.33)	8.43 (5.94, 12.61)	8.33 (6.18, 12.02)	4.16 (2.78, 6.08)	4.23 (2.79, 6.14)	4.02 (2.78, 5.81)
mECPP	DEHP	18.65 (12.39, 27.08)	18.19 (12.15, 26.44)	19.00 (12.69, 27.26)	9.13 (5.77, 15.35)	9.98 (5.93, 15.68)	8.83 (5.66, 14.83)
mEHHP	DEHP	12.45 (8.03, 19.33)	11.52 (7.55, 18.65)	13.18 (8.37, 19.72)	6.91 (4.71, 12.11)	7.43 (4.95, 12.30)	6.74 (4.23, 11.85)
mEOHP	DEHP	10.50 (6.79, 16.48)	9.70 (6.40, 15.83)	11.05 (7.42, 17.04)	3.87 (2.55, 7.03)	4.09 (2.72, 7.05)	3.75 (2.43, 6.74)
mBP	DBP	15.99 (10.69, 25.51)	15.40 (10.26, 23.14)	16.50 (10.98, 27.45)	6.72 (4.09, 12.65)	7.03 (4.31, 13.04)	6.44 (3.88, 12.25)
mIBP	DBP	19.66 (12.84, 34.02)	19.30 (12.76, 37.91)	20.65 (12.84, 31.92)	14.89 (9.21, 26.60)	15.86 (9.63, 28.28)	13.90 (8.82, 23.68)
mEP	DE phthalate	177.71 (67.94, 416.58)	183.44 (75.28, 435.74)	162.08 (66.27, 395.71)	13.82 (8.10, 30.85)	13.94 (8.71, 33.94)	13.55 (7.86, 25.64)
mMP	DMP	5.04 (3.49, 7.72)	5.08 (3.45, 7.54)	5.01 (3.52, 8.11)	2.46 (0.75, 4.60)	2.25 (0.54, 4.51)	2.71 (0.88, 4.83)
mCPP	DNOP	1.63 (1.16, 2.50)	1.54 (1.17, 2.33)	1.70 (1.15, 2.69)	1.32 (0.88, 2.20)	1.37 (0.92, 2.25)	1.27 (0.84, 2.18)
BPA	BPA	1.64 (1.05, 3.00)	1.57 (1.01, 3.01)	1.64 (1.08, 3.00)	0.56 (0.22, 1.30)	0.58 (0.24, 1.36)	0.54 (0.20, 1.20)
DEP	DAP	5.04 (3.14, 7.60)	4.61 (3.00, 6.89)	5.41 (3.30, 8.28)	3.27 (1.65, 6.66)	3.01 (1.62, 6.06)	3.39 (1.70, 7.05)
DETP	DAP	1.55 (0.83, 2.77)	1.45 (0.83, 2.67)	1.68 (0.83, 2.98)	0.31 (0.17, 0.81)	0.31 (0.16, 0.75)	0.31 (0.17, 0.85)
DMDTP	DAP	0.62 (0.36, 1.20)	0.60 (0.35, 1.14)	0.63 (0.36, 1.32)	N/A	N/A	N/A
DMP	DAP	15.29 (10.93, 21.20)	14.40 (10.73, 20.62)	15.95 (11.42, 21.51)	7.22 (4.47, 12.84)	7.23 (4.50, 12.88)	7.19 (4.39, 12.24)
DMTP	DAP	14.75 (9.94, 24.02)	13.96 (9.54, 23.31)	15.82 (10.64, 24.83)	5.02 (2.17, 10.78)	5.05 (2.13, 11.36)	5.00 (2.32, 10.34)

a Abbreviations: monobenzyl phthalate, mBzP; butyl benzyl phthalate, BBzP; mono-[(2-carboxymethyl)­hexyl] phthalate, mCMHP; mono-(2-ethyl-5-carboxypentyl) phthalate, mECPP; mono-(2-ethyl-5-hydroxyhexyl) phthalate, mEHHP; mono-(2-ethyl-5-oxohexyl) phthalate, mEOHP; di­(2-ethyhexyl) phthalate, DEHP; monobutyl phthalate, mBP; monoisobutyl phthalate, mIBP; dibutyl phthalate, DBP; monoethyl phthalate, mEP; diethyl phthalate, DE phthalate; monomethyl phthalate, mMP; dimethyl phthalate, DMP; mono­(3-carboxypropyl) phthalate, mCPP; di-n-octyl phthalate, DNOP; bisphenol A, BPA; diethyl phosphate, DEP; diethylthiophosphate, DETP; dimethyldithiophosphate, DMDTP; dimethylphosphate, DMP; dimethylthiophosphate, DMTP; dialkylphosphate, DAP; not applicable, N/A.

b All metabolite concentrations were adjusted for creatinine concentrations. Descriptive statistics of metabolite concentrations are presented as median (interquartile range) in units of ng/mL.

c Chemical concentrations between girls and boys were tested using the Mann–Whitney *U* test. No sex differences were observed for prenatal or childhood chemical concentrations (all *p* > 0.05), except prenatal DEP concentration (*p* = 0.005).

In the first step of the hBKMR models (with the full set of chemicals), di­(2-ethylhexyl) phthalate (DEHP), dibutyl phthalate (DBP), and DAP were identified as candidate chemical groups (group PIP ≥ 0.2) and included in the step-two mixture analyses (Table S6 for prenatal exposure and Table S7 for childhood exposure). In the hBKMR analysis of selected EDC mixture exposure during pregnancy (step two), we observed that higher levels of exposure to the mixture of DEHP, DBP, and DAP were associated with lower verbal comprehension scores (Figure 1A, orange line) and lower matrix reasoning scores (Figure 1B, orange line). When chemicals were simultaneously set at their 75th percentile levels compared to their median levels, average verbal comprehension scores at age 13 years were 0.33 points lower (95% CrI: −0.60, −0.06), and average matrix reasoning scores were 0.26 lower (95% CrI: −0.51, −0.02). In sex-stratified hBKMR models, a negative overall association between prenatal chemical mixture exposure and verbal comprehension scores was observed in boys (Figure 1A, purple line) but not in girls (Figure 1, A, green line). For matrix reasoning, boys showed lower scores with higher prenatal chemical mixture exposure levels, whereas girls showed an L-shaped nonlinear overall association (Figure 1B). For coding, a negative overall association was observed when all chemicals were below their median levels compared to the median in girls but not in boys (Figure 1D); however, there were less certainty about the overall association of prenatal chemical mixture exposure with digit span and coding (Figure 1C,D).

**Figure 1:**
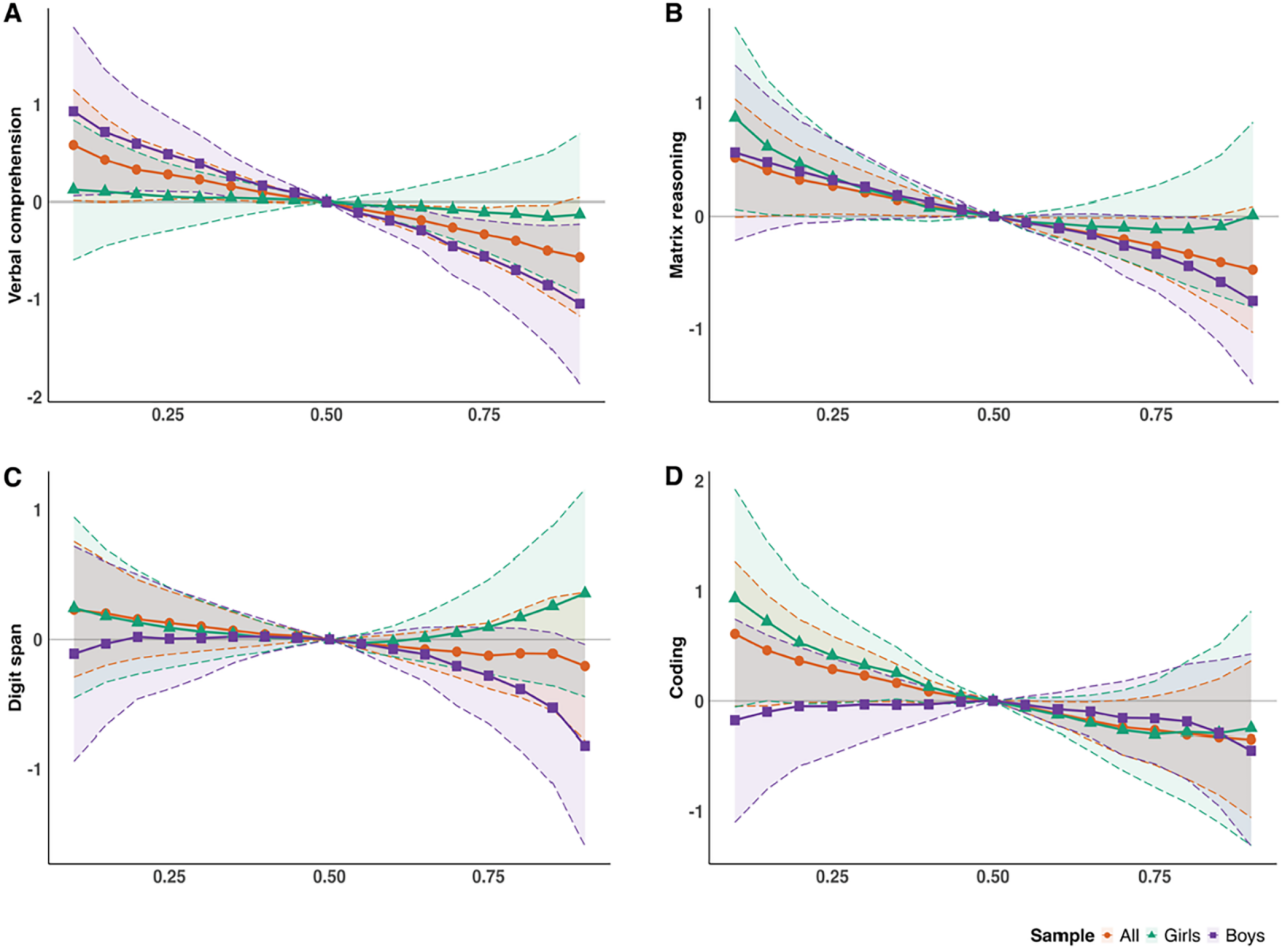
Overall effects of exposure to the mixture of phthalates, bisphenols, and organophosphate pesticides during pregnancy on cognitive performance scores in adolescence as estimated by hierarchical Bayesian kernel machine regression. The figures present estimated change in cognitive performance scores when all chemicals are at a set percentile on *x*-axis compared with when all chemicals are at their 50th percentile. Concentrations of chemicals were averaged across three time points during gestation, corrected for urinary dilution, log 2 transformed, and standardized. All models were adjusted for child age at cognitive performance assessment, child sex (when studying the total analysis sample), child migration background, maternal age, household income, maternal education, parity, maternal smoking during pregnancy, maternal prepregnancy BMI, and folic acid supplement use. The model of verbal score was additionally adjusted for maternal verbal IQ, and the models of other subtest scores were additionally adjusted for maternal nonverbal IQ.

We then explored the chemical groups and individual chemicals driving the overall associations between prenatal mixture exposure and cognitive performance. For verbal comprehension, no chemical group was identified as an important contributor to the overall mixture association (Figure 2A and Table S8). For matrix reasoning, we found that DBP (and its metabolite mBP) was the main contributor in girls (group PIP = 0.74; conditional PIP = 0.97), and DEHP (and its metabolite mECPP) was the primary contributor in boys (group PIP = 0.61; conditional PIP = 0.48) (Figure 2B and Table S8). Despite uncertainty in the overall association of prenatal chemical mixture exposure with digit span and coding scores, PIPs suggest that DBP and DEHP are the major contributors, respectively (Figure 2C,D and Table S8). From single-chemical analyses (Table S9), we found that metabolites of DBP and DAP were associated with verbal comprehension in boys, and metabolites of DEHP and DBP were associated with matrix reasoning in boys, which corresponds to chemicals with higher PIPs in the mixture analysis.

**Figure 2:**
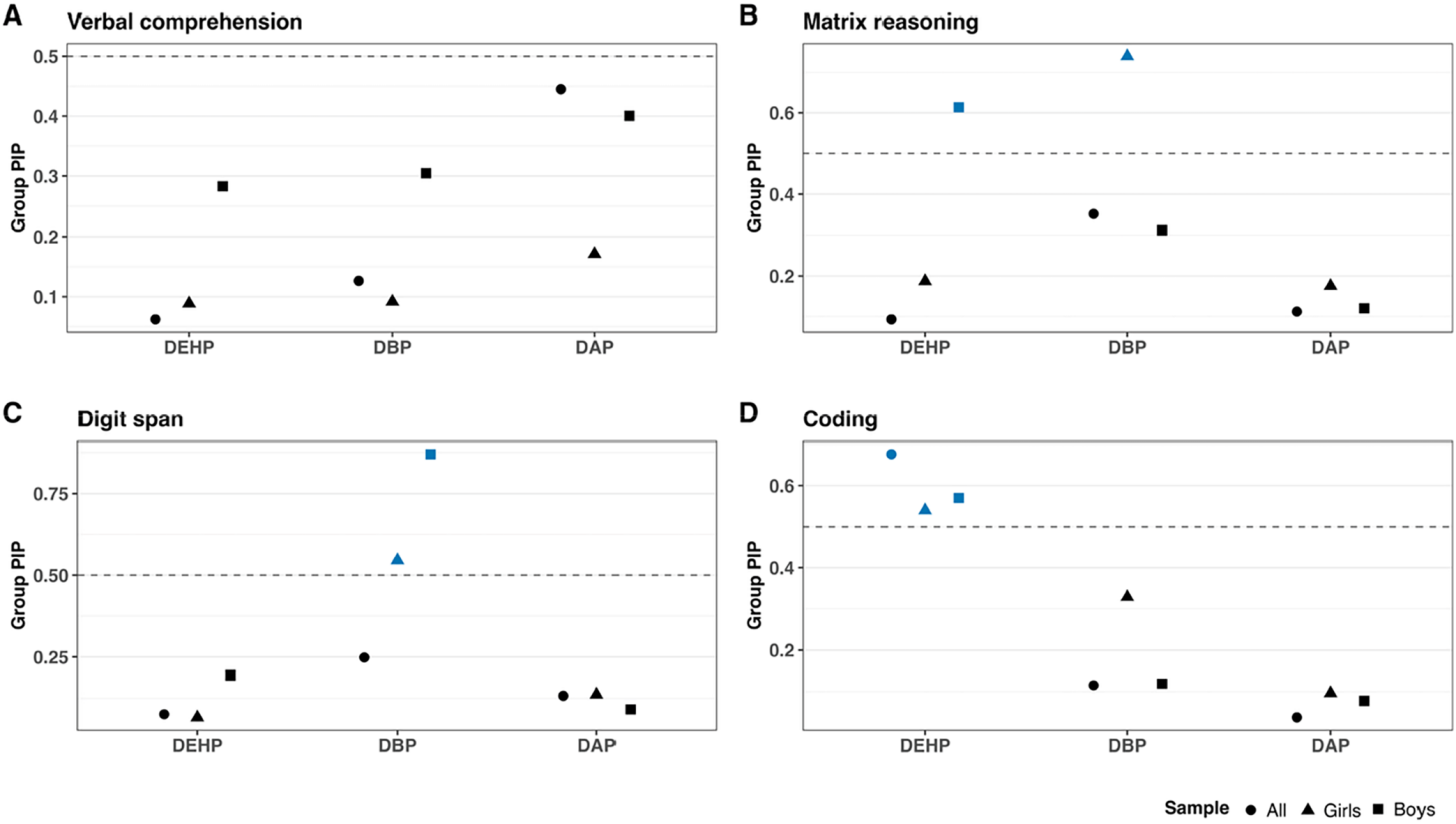
Posterior inclusion probability of prenatal chemical mixture exposure. Abbreviations: di­(2-ethyhexyl) phthalate, DEHP; dibutyl phthalate, DBP; dialkylphosphate, DAP. The figures show group posterior inclusion probabilities (PIP), representing the relative importance of each chemical group in relation to cognitive performance scores. We considered PIP ≥ 0.5 as the cutoff to flag chemical groups with important contribution to the overall mixture associations.

We found mostly linear associations of individual chemical exposures with verbal comprehension and matrix reasoning when fixing other chemicals at their median levels (Figure S6). The single-chemical associations provided no evidence of interactions among chemicals (Figure S7).

In hBKMR analyses of the selected EDC mixture exposure at age 6, higher mixture levels were associated with higher verbal comprehension scores at the age of 13 years (Figure 3A, orange line). For example, when chemicals were simultaneously set at their 75th percentile levels compared to their median levels, average verbal comprehension scores were 0.31 points higher (95% CrI: 0.01, 0.60). Sex-stratified analyses showed similar overall trends for girls (green line) and boys (purple line), with girls showing somewhat larger association estimates. There was no clear evidence for overall associations of the EDC mixture with matrix reasoning, digit span, or coding (Figure 2B–D). Regarding the contribution of components in the mixture to verbal comprehension, we found that DAP (and its metabolites DMP) was the main contributor for verbal comprehension (group PIP = 0.51; conditional PIP = 0.80) (Figure 4A and Table S10). Figure 4 also shows PIPs of chemical groups with other cognitive scales, even though the overall mixture associations with matrix reasoning, digit span, and coding were uncertain. We found no indication of nonlinearity of individual chemicals or interactions among chemicals for verbal comprehension (Figures S8 and S9). Results from the single-chemical analyses of childhood exposures showed that metabolites of DBP and DAP were associated with verbal comprehension (Table S11), consistent with chemicals with higher PIPs in the mixture analysis.

**Figure 3:**
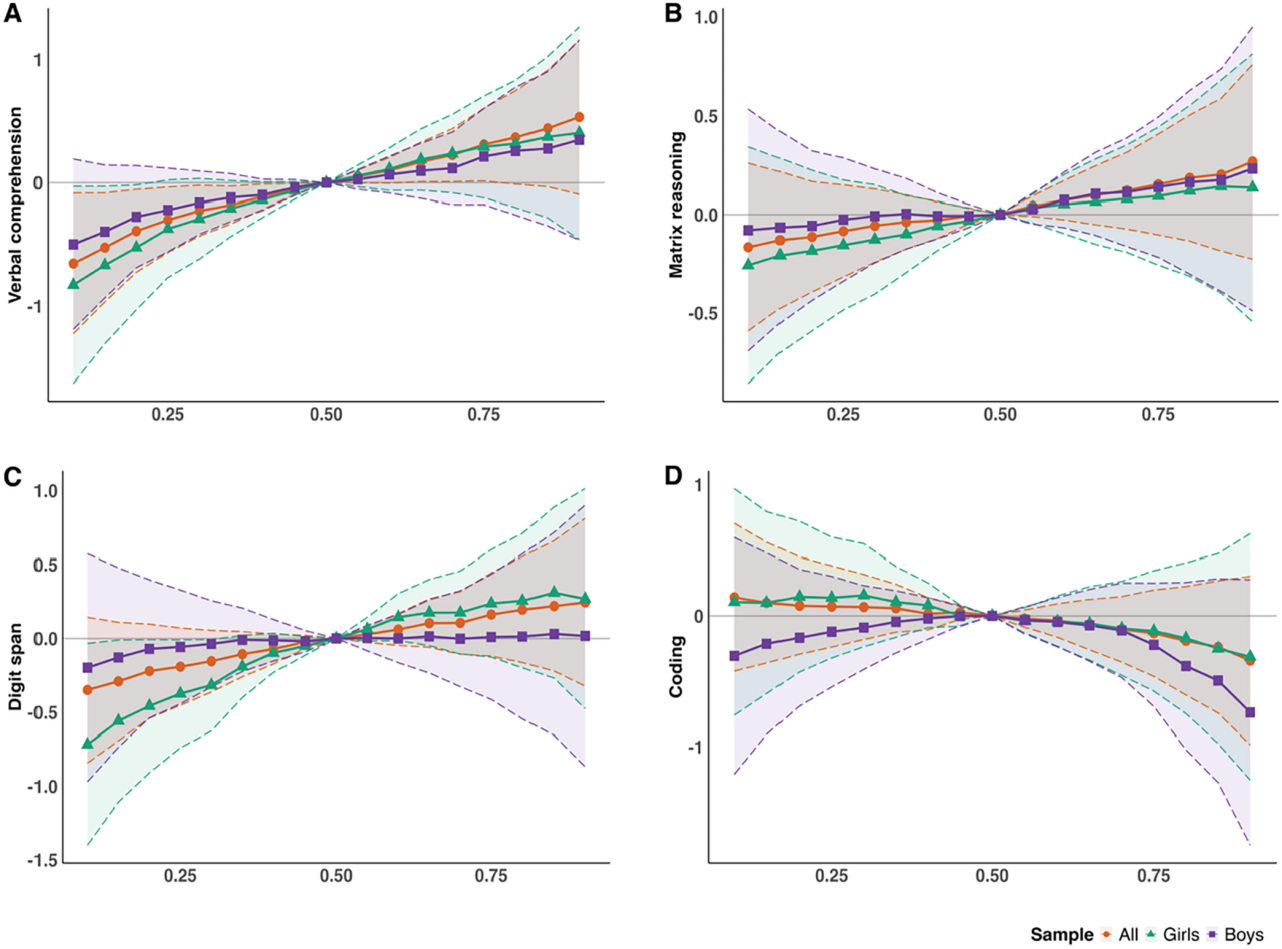
Overall effects of exposure to the mixture of phthalates, bisphenols, and organophosphate pesticides in childhood on cognitive performance scores in adolescence as estimated by hierarchical Bayesian kernel machine regression. The figures present estimated change in cognitive performance scores when all chemicals are at a set percentile on the *x*-axis compared with when all chemicals are at their 50th percentile. Concentrations of chemicals were corrected for urinary dilution, log 2 transformed, and standardized. All models were adjusted for child age at cognitive performance assessment, child sex (when studying the total analysis sample), child migration background, maternal age, household income, maternal education, parity, maternal smoking during pregnancy, maternal prepregnancy BMI, folic acid supplement use, and child BMI measured at age 5. The model of verbal score was additionally adjusted for maternal verbal IQ, and the models of other subtest scores were additionally adjusted for maternal nonverbal IQ.

**Figure 4:**
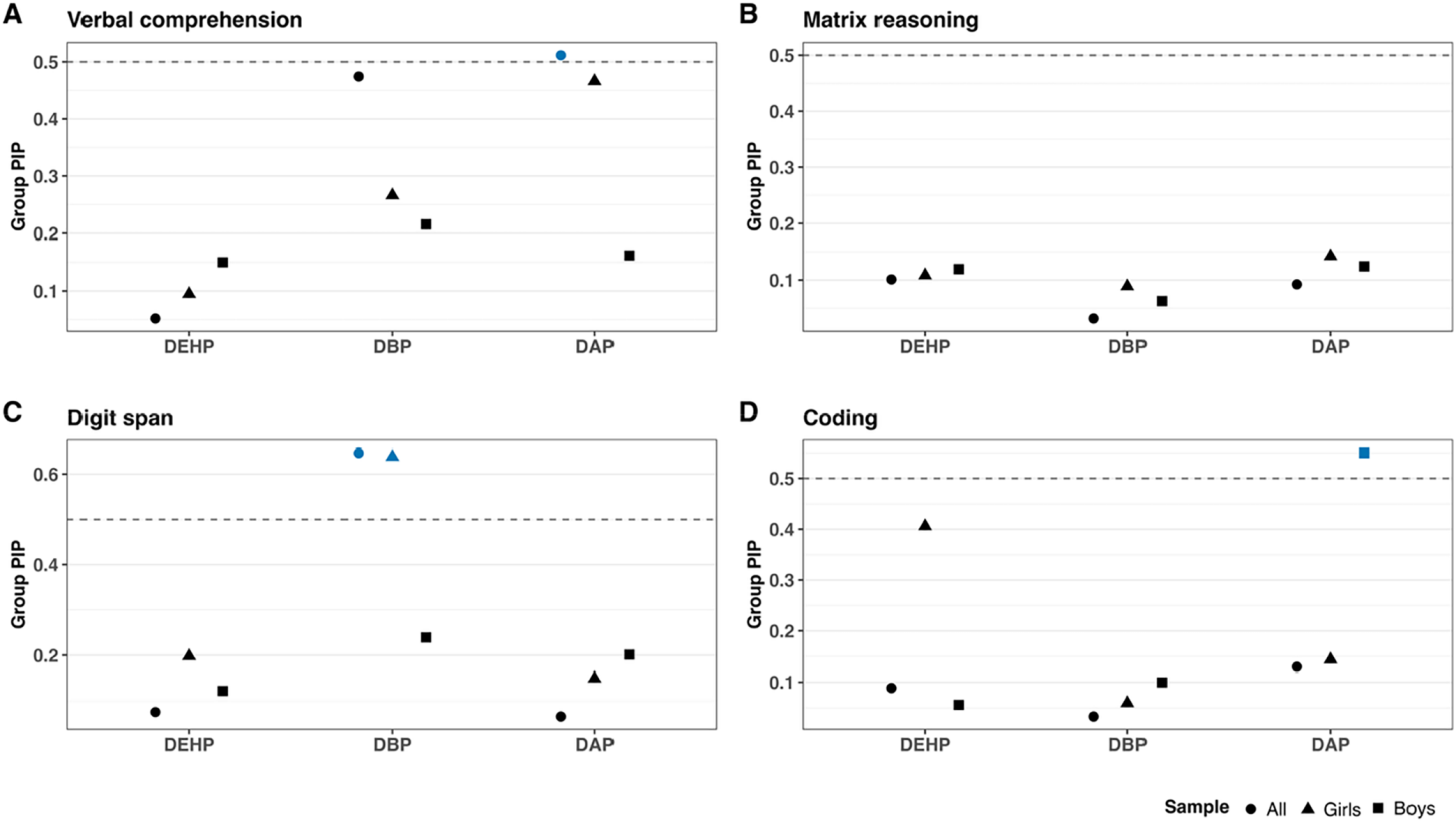
Posterior inclusion probability of childhood chemical mixutre exposure. Abbreviations: di­(2-ethyhexyl) phthalate, DEHP; dibutyl phthalate, DBP; dialkylphosphate, DAP. The figures show group posterior inclusion probabilities (PIP), representing the relative importance of each chemical group in relation to cognitive performance scores. We considered PIP ≥ 0.5 as the cutoff to flag chemical groups with important contribution to the overall mixture associations.

Results from the sensitivity analysis, which additionally adjusted for prenatal chemical exposure in the childhood mixture models, were consistent with those of the main analyses (Figure S10).

## Discussion

Using data from a longitudinal study from the general population, we provided new insights into long-term associations between prenatal and childhood exposure to an EDC mixture (BPA, phthalates, and OP pesticides) and domain-specific cognitive performance. We observed overall inverse associations of prenatal exposure to the EDC mixture with verbal comprehension and matrix reasoning in adolescents, with suggestive sex differences in terms of the magnitude and shape of the overall associations as well as the major contributing chemicals. In contrast, our data suggest a positive overall association between childhood EDC mixture exposure and verbal comprehension, independent of important confounders such as maternal verbal cognition. Major contributors to the prenatal or childhood mixture effects were DEHP, DBP, and nonspecific metabolites of OP pesticides. There was no clear evidence for overall associations of prenatal or childhood mixture exposure with digit span and coding scores. Furthermore, we found little indication of nonlinearity or interactions among chemicals in the associations of prenatal and childhood EDC mixture exposure with cognitive performance, suggesting additive and linear relationships between the EDC mixture and four domains of cognitive performance.

Our knowledge on potential mechanisms for brain influences of EDC exposure come from experimental research in animals and neuroimaging studies in humans. In vivo and in vitro studies indicated that phthalates, such as DEHP and DBP, exert developmental neurotoxicity through thyroid disruption, epigenetic modification, and sex hormone disruption,^41–43^ which may lead to impaired neurogenesis and increased apoptosis in the developing brain.^44,45^ In line with this, epidemiological studies have shown associations of prenatal phthalate exposure with thyroid function during early pregnancy.^46^ Moreover, pediatric neuroimaging studies have reported differences in brain morphological alterations mediating the link between prenatal phthalates exposure and offspring’s cognition and behaviors.^29,47^ Specifically, higher prenatal mIBP (a metabolite of DBP) and high-molecular-weight phthalates (including DEHP) were associated with smaller cerebral white matter volumes and altered frontoparietal white matter tracts that may support fluid intelligence.^29,48^ OP pesticides exert neurodevelopmental toxicity through disruption of cholinergic signaling processes, and by inducing oxidative stress, neuroinflammation, and epigenetic changes.^49–53^ Neuroimaging studies of children prenatally exposed to OP pesticides show changes in frontotemporal and parietal cortical morphology, differences in white matter microstructure, and altered cortical brain activation during executive function and language comprehension tasks a language test in childhood and adolescence.^53–56^ Taken together, these pathways may jointly contribute to the observed association between early-life chemical mixture exposure and adolescents’ cognitive performance.

We found that higher prenatal EDC mixture exposure was associated with lower verbal comprehension and matrix reasoning scores in adolescence, with phthalates as primary contributors, independent of maternal lifestyle during pregnancy, socioeconomic status, and maternal IQ. These findings are in line with previous studies conducted in European and North American cohorts, including one from the Generation R Study, which reported negative overall associations between prenatal exposure to an EDC mixture including common plasticizers and pesticides and cognitive performance in 3–8 years children.^15–17,57,58^ These studies consistently reported phthalates as key contributors to mixture effects. A recent single-chemical study in the Generation R cohort further supports these findings, showing that prenatal exposure to two phthalate metabolites (i.e., mBP and mEP) was associated with poorer verbal comprehension and matrix reasoning in adolescence.^29^ However, findings for domain-specific cognitive performance across studies remain mixed. Some studies observed inverse associations of the EDC mixture and performance IQ or nonverbal IQ,^16,17,58^ whereas others reported null associations with nonverbal IQ,^59,60^ or positive associations with verbal IQ (not significant).^58,60^ Heterogeneity in the timing of exposure assessment and concentrations in the population, chemical mixture composition, and confounder adjustment (e.g., maternal IQ) likely contributes to the discrepancies in findings. Our study extends prior work by investigating long-term joint associations between prenatal nonpersistent EDCs exposure and domain-specific cognitive outcomes in adolescence while adjusting for key confounders. The findings suggest that prenatal exposure to a mixture of common plasticizers and OP pesticides may have long-lasting and domain-specific impacts on adolescent cognition, particularly in verbal comprehension and nonverbal fluid reasoning.

For childhood EDC mixture exposure, we observed a positive trend with verbal comprehension, driven by nonspecific metabolites of OP pesticides. Results from the sensitivity analysis, in which childhood chemical mixture exposure models were additionally adjusted for prenatal chemical exposures, suggest that residual confounding by prenatal exposures is unlikely to explain the observed positive overall associations. Evidence on the associations between childhood EDCs exposure and cognitive outcomes is limited. One prospective birth cohort study in Korea (*n* = 47) examined early childhood exposure to a mixture of bisphenol A and three phthalates and children’s IQ at the age 5 years, reporting a negative overall association with mEOHP (a metabolite of DEHP) as the main contributor.^18^ Most childhood chemical exposure studies have been single-pollutant and cross-sectional, and they consistently report negative associations between phthalates and IQ,^20,61–64^ with limited evidence for OP pesticides.^51^ We extended current evidence by examining the longitudinal association of EDC mixture at the age of 6 years with cognitive performance in adolescence. The unexpected finding with childhood chemical exposure should be interpreted with caution for the following reasons. First, childhood exposure was assessed using one single spot urine samples and thus exposure misclassification in childhood exposure cannot be entirely ruled out. Second, while comparison of the samples with prenatal and childhood exposure data suggests that the two samples were similar in characteristics for which we had information on, potential selection bias and unmeasured confounding factors (e.g., parental perception of behavior problems, socioeconomic challenges, or the home learning environment)^65^ in the childhood exposure associations can influence the results. Lastly, exposure to pesticides happens usually through food in urban nonoccupational settings, which can also be an important source of nutrients supporting neurodevelopment. As such, positive residual confounding is also plausible if children with higher childhood phthalate, bisphenol, and OP pesticide metabolite levels in our analytic sample were disproportionately drawn from health conscious households that both raise OP pesticide exposure through greater fresh fruit/vegetable intake^24,66^ and avoid BPA by substituting other plasticizers,^67^ while also providing richer language stimulation that benefits verbal skills, creating a spurious positive association. Overall, although a true positive signal of phthalates and OP pesticides cannot be excluded, replication of this finding in larger longitudinal cohorts with repeated biospecimens and measures of the home environment is warranted.

We observed some sex differences in the associations of prenatal EDCs mixture exposure with adolescents’ verbal comprehension. The inverse association between prenatal chemical mixture exposure and verbal comprehension was mainly driven by associations in boys. Also, the chemicals driving the association of the prenatal EDC mixture with matrix reasoning varied between boys and girls: DEHP metabolites were the main driver in boys, and DBP metabolites were most important in girls. Mechanisms involving sex hormone disruption may underlie these differences, as fetal brain development and sexual differentiation largely depend on gonadal steroids during sensitive periods of development,^68^ and individual phthalate compounds can disrupt sex steroid pathways through compound-specific mechanisms in a sex-dependent way. For instance, animal studies indicate that DEHP shows antiandrogenic activity and interfere with brain aromatase in the developing brain, which may alter brain-derived estradiol during male-typical windows of brain organization that are important for synaptogenesis and myelination.^69–71^ In contrast, DBP disrupts estrogen-related signaling in the developing brain, including alterations in aromatase activity and estrogen receptor-β expression in the hippocampus,^43^ which may partly explain a stronger contribution of prenatal DBP exposure to the overall association with matrix reasoning in girls.

This study included a large sample size with repeated measures several EDCs across two life stages important for brain development, and we had information on important confounders, such as maternal IQ. The rich data structure allowed us to use of a flexible mixture modeling method that captures complex exposure–outcome responses and facilitate identifying chemicals of concern with reduced number of testings. Nevertheless, it is important to interpret our results within the context of several limitations. Phthalates, bisphenols, and OP pesticides are nonpersistent chemicals with short half-lives. Although we used three spot urine samples during pregnancy to proxy average exposure across gestation, measurement error may still have an implication in imprecise effect estimates.^72^ In addition, childhood exposure was assessed from a single spot urine sample, making estimates prone to attenuation bias from nondifferential exposure misclassification.^73^ Although we adjusted for several important confounder including socioeconomic status, maternal/child lifestyle factors, and mother’s nonverbal/verbal IQ, residual confounding by genetic factors (e.g., the paraoxonase-1 gene that plays a key role in the detoxification of OP pesticides and has been linked to child neurobehavioral development)^74^ and unmeasured environmental confounding (e.g., stimuli in home environment) remains possible given the observational nature of the study. In particular, our findings on childhood exposure should be interpretated with caution and replicated in other longitudinal cohort studies with repeated urine samples, diet, and home environment measures.

In conclusion, prenatal exposure to a mixture of common plasticizers and OP pesticides might have a modest and adverse effect on offspring’s neurodevelopment, especially on verbal comprehension and matrix reasoning during adolescence. We observed sex differences in the chemical group contributing to the overall mixture, suggesting boys and girls may differ in susceptibility to specific compounds, potentially through sex hormone pathways. More mechanistic studies are needed to confirm the biological relevance of our findings. In contrast, we observed a positive overall association of childhood exposure to a mixture of plasticizers and OP pesticides with verbal comprehension in adolescence. However, this finding was unexpected and should be interpreted with caution pending replication in other cohort studies.

## Supplementary Material



## Data Availability

Data described in the manuscript cannot be made publicly available because of confidentiality, but data can be requested and shared with a formal data-sharing agreement. Requests for data, code book, and analytic code can be directed to datamangementgenr@erasmusmc.nl.

## Notes

Table S1 presents the percentages of missing values in covariates; Table S2 presents chemical groups and associated metabolites; Table S3 shows the characteristics of full sample and samples of respondents with chemical measurements; Tables S4 and S5 present the descriptive statistics of metabolite concentration of chemical exposure during pregnancy by weeks of gestation and in childhood; Tables S6 and S7 show the posterior inclusion probability of prenatal and childhood chemical exposure obtained using BKMR with the full set of chemicals; Tables S8 and S10 show the posterior inclusion probability of prenatal and childhood chemical exposure obtained using BKMR with selected chemicals; Tables 9 and S11 present single-chemical associations of individual chemical exposure during pregnancy and in childhood with cognitive performance subtest scores; Figure S1 illustrates the flowchart of the study population; Figure S2 presents the directed acyclic graphs; Figures S3–S5 show correlation matrices for prenatal and childhood exposure biomarker concentrations; Table S6 (prenatal exposure) and S8 (childhood exposure) visualize univariate exposure–response functions for each metabolite with the other metabolites fixed at the median from BKMR; Tables S7 and S9 visualize single-metabolite associations during pregnancy and in childhood with cognitive performance scores from BKMR; and Figure S10 shows the overall effects of exposure to the chemical mixture in childhood on cognitive performance scores in models additionally adjusted for prenatal chemical exposure (PDF)
