# Supplementary material for: Associations of Exposure to Common Plasticizers and Organophosphate Pesticides during Pregnancy and in Childhood with Cognitive Performance in Adolescents: A Population-Based Study
Source: Environ Sci Technol. 2026 Jun 12;60(25):17652–65. doi: 10.1021/acs.est.5c18986 (PMC13325851; doi:10.1021/acs.est.5c18986)

Supporting Information

Associations of exposure to common plasticizers and organophosphate pesticides during pregnancy and in childhood with cognitive performance in adolescents: A population-based study

Yuchan Mou, Hanan El Marroun, Mengling Liu, Arash Derakhshan, Mònica Guxens, Vincent W. Jaddoe, Tonya White, Kurunthachalam Kannan, Suzanne Spaan, Anjoeka Pronk, Leonardo Trasande, Hening Tiemeier, Akhgar Ghassabian

The supporting information consists of 11 supplemental tables and 10 supplemental figures on 43 pages.

Table of Content

Table S 1. Percentages of missing values in covariates
Table S 2. Chemical groups and associated metabolites
Table S 3. Characteristics of full sample and samples of respondents with chemical measurements
Table S 4. Distributions of metabolite concentration of chemical exposure during pregnancy by weeks of gestation
Table S 5. Distributions of metabolite concentration of chemical exposure in childhood
Table S 6. Posterior inclusion probability of prenatal chemicals exposure obtained using BKMR with the full set of chemicals
Table S 7. Posterior inclusion probability of childhood chemicals exposure obtained using BKMR with the full set of chemicals
Table S 8. Posterior inclusion probability of prenatal chemicals exposure obtained using hBKMR with selected chemicals
Table S 9. Single-chemical associations of individual chemical exposure during pregnancy with cognitive performance subtest scores
Table S 10. Posterior inclusion probability of childhood chemicals exposure obtained using hBKMR with selected chemicals
Table S 11. Single-chemical associations of individual chemical exposure in childhood with cognitive performance subtest scores

Figure S 1. Flowchart of the study population
Figure S 2. Directed acyclic graphs
Figure S 3. Correlation matrix for prenatal exposure biomarker concentrations
Figure S 4. Correlation matrix for childhood exposure biomarker concentrations
Figure S 5. Correlation matrix for prenatal and childhood exposure biomarker concentrations
Figure S 6. Univariate exposure-response functions and 95% credible intervals for each metabolite with the other metabolites fixed at the median (prenatal exposure)
Figure S 7. Single metabolite of prenatal EDC mixture associations with cognitive performance scores in adolescence as estimated by BKMR
Figure S 8. Univariate exposure-response functions and 95% credible intervals for each metabolite with the other metabolites fixed at the median (childhood exposure)
Figure S 9. Single metabolite of childhood EDC mixture associations with cognitive performance scores in adolescence as estimated by BKMR
Figure S 10. Overall effects of exposure to the mixture of phthalates, bisphenols and organophosphate pesticides in childhood on cognitive performance scores in adolescence; model additionally adjusted for prenatal phthalic acid, bispenol A and non-specific dialkylphosphate metabolites

Table S 1. Percentages of missing values in covariates

	Mother-child pairs with exposure data during pregnancy n = 565	Mother-child pairs with exposure data during childhood n = 539
Maternal characteristics		
Age at enrollment	0.0%	0.0%
Household income per month	11.5%	11.3%
Education level	7.4%	7.8%
National origin	0.0%	0.0%
Parity	0.4%	0.4%
Pre-pregnancy BMI	12.7%	13.2%
Smoking during pregnancy	8.0%	8.2%
Folic acid use	19.7%	19.7%
Non-verbal IQ	2.1%	2.2%
Verbal IQ	17.0%	16.9%
Child characteristics		
Age at the cognitive assessment	0.0%	0.0%
Sex	0.0%	0.0%
Migration background	0.0%	0.0%
zBMI measured at age 5 years	0.0%	0.0%

Table S 2. Chemical groups and associated metabolites

Parent compounds/Chemical groups	Metabolites
Butyl benzyl phthalate (BBzP)	Monobenzyl phthalate (mBzP)
Di(2-ethyhexyl) phthalate (DEHP)	Mono-(2-ethyl-5-carboxypentyl) phthalate (mECPP)
	Mono-(2-ethyl-5-hydroxyhexyl) phthalate (mEHHP)
	Mono-(2-ethyl-5-oxohexyl) phthalate (mEOHP)
	Mono-[(2-carboxymethyl)hexyl] phthalate (mCMHP)
Dibutyl phthalate (DBP)	Monobutyl phthalate (mBP)
	Monoisobutyl phthalate (mIBP)
Diethyl phthalate (DE phthalate)	Monoethyl phthalate (mEP)
Dimethyl phthalate (DMP)	Monomethyl phthalate (mMP)
Di-n-octyl phthalate (DNOP)	Mono(3-carboxypropyl) phthalate (mCPP)
Bisphenols	Bisphenol A (BPA)
Dialkylphosphate (DAP)	Diethyl phosphate (DEP)
	Diethylthiophosphate (DETP)
	Dimethyldithiophosphate (DMDTP)
	Dimethylphosphate (DMP)
	Dimethylthiophosphate (DMTP)

Table S 3. Characteristics of full sample and samples of respondents with chemical measurements

	Full sample<br>N = 8879	Respondents with chemical measurements in pregnancy<br>n = 775	Respondents with chemical measurements in childhood<br>n = 742
Maternal characteristics			
Age at enrollment, mean (SD), years	29.7 (5.3)	30.7 (4.6)	30.7 (4.7)
Household income per month, N (%)			
< €1200	2290 (25.8)	122 (15.8)	119 (16.1)
€1200 - €2200	2335 (26.3)	181 (23.4)	171 (23.1)
> €2200	4253 (47.9)	471 (60.8)	451 (60.9)
Education level (Low), N (%)	4617 (52.0)	292 (37.7)	277 (37.4)
National origin (Dutch), N (%)	4270 (48.1)	444 (57.3)	428 (57.7)
Parity, median (Nulliparous), N (%)	4936 (55.6)	484 (62.5)	465 (62.7)
Pre-pregnancy BMI	23.7 (4.4)	23.5 (4.2)	23.5 (4.3)
Smoking during pregnancy, N (%)			
Never	6446 (72.6)	595 (76.9)	567 (76.5)
Until pregnancy was known	781 (8.8)	68 (8.8)	68 (9.2)
Continued	1651 (18.6)	110 (14.2)	106 (14.3)
Folic acid use, N (%)			
No	2814 (31.7)	144 (18.6)	138 (18.6)
Started the first 10 weeks	2743 (30.9)	258 (33.4)	247 (33.3)
Started periconceptional	3320 (37.4)	371 (47.9)	356 (48.1)

Non-verbal IQ, mean (SD)	8.7 (2.4)	9.3 (2.2)	9.3 (2.2)
Verbal IQ, mean (SD)	30.0 (12.3)	32.7 (11.9)	32.7 (12.0)
Child characteristics			
Age at the cognitive assessment, mean (SD), years	13.6 (0.4)	13.5 (0.3)	13.5 (0.3)
Sex (Girls), N (%)	4403 (49.6)	385 (49.7)	362 (48.8)
Migration background (Dutch), N (%)	4608 (51.9)	474 (61.2)	456 (61.5)
zBMI measured at age 5 years, mean (SD)	0.3 (1.0)	0.2 (0.9)	0.2 (0.9)
Verbal score, mean (SD)	9.3 (2.9)	9.6 (2.9)	9.6 (2.9)
Coding score, mean (SD)	12.7 (3.3)	12.8 (3.2)	12.8 (3.2)
Digit Span score, mean (SD)	9.3 (2.7)	9.5 (2.7)	9.5 (2.7)
Matrix reasoning score, mean (SD)	9.0 (2.6)	9.2 (2.5)	9.2 (2.5)

Values are mean (standard deviation, SD) for continuous variables with a normal distribution or valid numbers (%) for categorical variables. Missing data of covariates were imputed with multiple imputation (m = 30 imputations)

Table S 4. Distributions of metabolite concentration of chemical exposure during pregnancy by weeks of gestation

Metabolites[a]	Parent compounds	Minimum	10%	25%	50%	75%	90%	99%	Maximum	Percentage below the LOD	ICC[b]
mBzP	BBzP										0.31
<18 weeks		0.15	1.25	3.13	6.14	12.19	23.58	89.80	314.12	16.11	
18-25 weeks		0.20	1.04	2.19	5.25	9.69	21.26	117.21	363.64	0.53	
>25 weeks		0.16	0.45	0.99	2.27	4.40	8.70	32.93	128.00	3.72	
mCMHP	DEHP										0.15
<18 weeks		0.30	3.80	6.66	13.07	22.91	39.73	158.70	893.90	0.18	
18-25 weeks		0.24	1.20	2.35	4.36	7.68	12.92	64.76	5497.20	0.35	
>25 weeks		0.07	1.00	1.84	3.47	6.51	10.60	30.50	132.98	1.06	
mECPP	DEHP										0.30
<18 weeks		0.52	3.80	7.65	15.40	29.59	58.28	263.34	906.05	0.18	
18-25 weeks		0.48	2.90	5.69	10.46	19.78	33.76	161.41	93301.21	0.00	
>25 weeks		0.48	5.49	9.25	17.26	32.21	62.31	134.54	451.42	0.00	
mEHHP	DEHP										0.24
<18 weeks		0.09	2.06	5.21	10.98	22.06	41.33	139.33	827.19	0.35	
18-25 weeks		0.40	1.59	3.15	6.09	12.25	21.66	75.83	153.65	0.00	
>25 weeks		0.11	2.48	4.58	9.44	18.27	31.58	136.33	336.87	0.18	
mEOHP	DEHP										0.13
<18 weeks		0.08	1.35	3.22	7.52	15.37	30.16	96.47	613.93	0.00	
18-25 weeks		0.51	2.22	4.05	9.24	20.08	34.65	141.05	2021.66	0.00	
>25 weeks		0.39	1.97	3.01	5.81	12.38	20.19	102.14	167.67	0.18	
mBP	DBP										0.19
<18 weeks		0.32	3.24	6.70	16.07	31.53	64.04	211.97	2715.56	0.88	
18-25 weeks		0.96	2.74	5.15	10.25	19.34	35.25	132.30	211.07	0.00	
>25 weeks		0.20	2.68	5.19	10.22	19.86	41.21	168.09	823.15	0.35	
mIBP	DBP										0.36
<18 weeks		0.28	4.75	9.31	21.01	45.24	86.22	517.96	1826.57	0.35	
18-25 weeks		0.76	2.77	5.08	10.45	19.41	36.92	164.63	331.34	0.00	
>25 weeks		0.92	4.25	7.51	13.07	27.11	61.85	255.88	543.13	0.18	

mEP	DE phthalate										0.64
<18 weeks		1.33	14.03	33.44	112.25	366.37	1048.59	4011.28	9482.73	0.18	
18-25 weeks		1.53	9.53	26.75	77.75	252.86	657.96	3466.09	10214.42	0.00	
>25 weeks		1.42	16.85	40.29	122.85	406.85	1269.82	5576.19	8509.85	0.00	
mMP	DMP										0.50
<18 weeks		0.24	1.32	2.57	4.96	9.13	15.77	117.68	287.95	0.18	
18-25 weeks		0.08	1.15	1.95	3.97	6.83	12.33	142.61	343.11	0.18	
>25 weeks		0.12	1.20	2.06	4.12	8.19	15.21	212.47	498.82	0.35	
mCPP	DNOP										0.29
<18 weeks		0.01	0.35	0.67	1.30	2.46	4.25	20.17	120.23	0.00	
18-25 weeks		0.07	0.29	0.53	0.90	1.60	2.94	10.13	43.06	0.00	
>25 weeks		0.12	0.51	1.03	1.77	2.96	5.55	19.94	60.40	0.18	
BPA	BPA										0.09
<18 weeks		0.15	0.37	0.70	1.61	3.58	7.35	45.57	90.21	27.79	
18-25 weeks		0.15	0.48	0.90	1.72	3.55	7.31	41.31	109.48	6.19	
>25 weeks		0.15	0.39	0.76	1.58	2.85	6.25	29.32	64.85	11.86	
DEP	DAP										0.58
<18 weeks		-0.04	0.93	1.85	3.67	7.13	14.27	42.84	1035.69	3.01	
18-25 weeks		0.00	0.77	1.71	3.40	6.49	12.01	53.25	168.46	4.42	
>25 weeks		0.07	0.90	1.70	3.52	7.36	13.70	40.27	77.45	3.54	
DETP	DAP										0.35
<18 weeks		-0.08	0.08	0.35	1.00	2.37	6.12	27.74	117.24	12.04	
18-25 weeks		-0.08	0.10	0.34	0.86	2.22	4.48	20.55	99.87	10.97	
>25 weeks		-0.05	0.11	0.35	0.98	2.49	5.33	21.64	64.89	10.27	
DMDTP	DAP										0.35
<18 weeks		0.00	0.00	0.14	0.36	0.95	2.17	16.23	75.58	20.18	
18-25 weeks		0.00	0.06	0.15	0.37	0.94	2.27	9.86	20.69	17.70	
>25 weeks		0.00	0.05	0.15	0.36	0.86	2.55	19.46	38.01	17.70	
DMP	DAP										0.50
<18 weeks		0.12	3.44	6.07	12.07	21.55	40.27	103.36	173.79	0.18	
18-25 weeks		0.60	3.36	6.76	12.60	21.95	33.69	78.08	181.35	0.00	
>25 weeks		0.74	3.38	6.21	11.59	20.90	37.86	131.37	200.28	0.00	

DMTP	DAP										0.41
<18 weeks		0.00	1.97	4.44	10.72	20.98	44.05	125.86	276.24	3.36	
18-25 weeks		0.00	2.10	4.81	12.50	23.98	42.28	110.29	205.07	3.54	
>25 weeks		0.00	2.52	5.35	10.98	22.06	43.57	154.92	284.85	2.48	

Abbreviations: monobenzyl phthalate, mBzP; butyl benzyl phthalate, BBzP; mono-[(2-carboxymethyl)hexyl] phthalate, mCMHP; mono-(2-ethyl-5-carboxypentyl) phthalate, mECPP; mono-(2-ethyl-5-hydroxyhexyl) phthalate, mEHHP; mono-(2-ethyl-5-oxohexyl) phthalate, mEOHP; di(2-ethyhexyl) phthalate, DEHP; monobutyl phthalate, mBP; monoisobutyl phthalate, mIBP; dibutyl phthalate DBP; monoethyl phthalate, mEP; diethyl phthalate, DE phthalate; monomethyl phthalate, mMP; dimethyl phthalate, DMP; mono(3-carboxypropyl) phthalate, mCPP; di-n-octyl phthalate, DNOP; bisphenol A, BPA; diethyl phosphate, DEP; diethylthiophosphate, DETP; dimethyldithiophosphate, DMDTP; dimethylphosphate, DMP; dimethylthiophosphate, DMTP; dialkylphosphate, DAP; limit of detection, LOD; intraclass correlation coefficient, ICC.

[a] Descriptive statistics of concentration for metabolites are in the unit of ng/mL. Concentartion below the LOD are excluded.

[b] ICC for the three measurements across pregnancy were assessed using two-way mixed effects models with multiple measurements and absolute agreement.

Table S 5. Distributions of metabolite concentration of chemical exposure in childhood

Metabolites	Parent compounds	Min	10%	25%	50%	75%	90%	99%	Max	Percentage below the LOD
mCMHP	DEHP	0.23	1.28	2.37	4.03	6.65	10.98	29.04	110.98	0.00
mECPP	DEHP	0.45	2.41	4.53	9.16	16.28	29.98	74.21	182.98	0.00
mEHHP	DEHP	0.42	2.33	4.06	7.14	12.25	21.12	45.47	89.70	0.00
mEOHP	DEHP	0.25	1.22	2.21	3.91	6.98	11.72	30.05	53.70	0.00
mBP	DBP	0.32	1.67	3.50	6.86	12.95	25.69	79.68	341.85	1.86
mIBP	DBP	1.19	5.03	8.42	14.00	26.02	50.70	156.50	586.00	0.56
mEP	DE phthalate	0.82	3.31	6.27	14.20	35.45	116.40	951.36	2660.00	0.00
mMP	DMP	0.06	0.52	1.64	3.71	6.43	10.32	26.74	70.00	17.07
mCPP	DNOP	0.04	0.40	0.70	1.34	2.38	5.33	31.32	425.00	0.00
BPA	BPA	0.15	0.27	0.50	0.86	1.57	2.76	16.89	101.79	26.90
DEP	DAP	0.00	0.77	1.46	3.09	6.73	12.58	34.54	129.88	4.45
DETP	DAP	-0.05	0.01	0.10	0.31	0.89	2.00	8.44	14.01	29.68
DMP	DAP	0.00	1.91	3.72	7.09	12.56	21.89	56.52	125.37	0.56
DMTP	DAP	0.00	0.74	1.93	4.83	11.01	24.57	91.29	158.25	6.86

Abbreviations: mono-[(2-carboxymethyl)hexyl] phthalate, mCMHP; mono-(2-ethyl-5-carboxypentyl) phthalate, mECPP; mono-(2-ethyl-5-hydroxyhexyl) phthalate, mEHHP; mono-(2-ethyl-5-oxohexyl) phthalate, mEOHP; di(2-ethyhexyl) phthalate, DEHP; monobutyl phthalate, mBP; monoisobutyl phthalate, mIBP; dibutyl phthalate DBP; monoethyl phthalate, mEP; diethyl phthalate, DE phthalate; monomethyl phthalate, mMP; dimethyl phthalate, DMP; mono(3-carboxypropyl) phthalate, mCPP; di-n-octyl phthalate, DNOP; bisphenol A, BPA; diethyl phosphate, DEP; diethylthiophosphate, DETP; dimethylphosphate, DMP; dimethylthiophosphate, DMTP; dialkylphosphate, DAP; limit of detection, LOD.

Descriptive statistic of concentration for metabolites are in the unit of ng/mL. Concentartion below the LOD are excluded.

Table S 6. Posterior inclusion probability of prenatal chemicals exposure obtained using BKMR with the full set of chemicals

		Verbal comprehension			Matrix reasoning			Digit span			Coding		
Chemicals	Group	All	Boys	Girls	All	Boys	Girls	All	Boys	Girls	All	Boys	Girls
mBzP	BBzP	0.08<br>(1.00)	0.15<br>(1.00)	0.04<br>(1.00)	0.09<br>(1.00)	0.14<br>(1.00)	0.03<br>(1.00)	0.03<br>(1.00)	0.02<br>(1.00)	0.11<br>(1.00)	0.01<br>(1.00)	0.01<br>(1.00)	0.01<br>(1.00)
mCMHP	DEHP	0.03<br>(0.10)	0.16<br>(0.17)	0.04<br>(0.35)	0.05<br>(0.17)	**0.51**<br>**(0.09)**	0.04<br>(0.23)	0.03<br>(0.09)	0.09<br>(0.15)	0.03<br>(0.35)	**0.44**<br>**(0.88)**	**0.31**<br>**(0.07)**	**0.35**<br>**(0.28)**
mECPP	DEHP	0.03<br>(0.43)	0.16<br>(0.48)	0.04<br>(0.21)	0.05<br>(0.35)	**0.51**<br>**(0.58)**	0.04<br>(0.19)	0.03<br>(0.32)	0.09<br>(0.25)	0.03<br>(0.19)	**0.44**<br>**(0.01)**	**0.31**<br>**(0.32)**	**0.35**<br>**(0.26)**
mEHHP	DEHP	0.03<br>(0.28)	0.16<br>(0.19)	0.04<br>(0.19)	0.05<br>(0.30)	**0.51**<br>**(0.21)**	0.04<br>(0.27)	0.03<br>(0.25)	0.09<br>(0.35)	0.03<br>(0.20)	**0.44**<br>**(0.08)**	**0.31**<br>**(0.14)**	**0.35**<br>**(0.36)**
mEOHP	DEHP	0.03<br>(0.19)	0.16<br>(0.16)	0.04<br>(0.25)	0.05<br>(0.18)	**0.51**<br>**(0.12)**	0.04<br>(0.32)	0.03<br>(0.34)	0.09<br>(0.25)	0.03<br>(0.27)	**0.44**<br>**(0.03)**	**0.31**<br>**(0.46)**	**0.35**<br>**(0.10)**
mBP	DBP	0.05<br>(0.78)	0.16<br>(0.84)	0.03<br>(0.42)	**0.23**<br>**(0.51)**	**0.25**<br>**(0.83)**	**0.40**<br>**(0.95)**	0.11<br>(0.32)	**0.78**<br>**(0.99)**	**0.28**<br>**(0.14)**	0.07<br>(0.81)	0.06<br>(0.78)	**0.21**<br>**(0.61)**
mIBP	DBP	0.05<br>(0.22)	0.16<br>(0.16)	0.03<br>(0.58)	**0.23**<br>**(0.49)**	**0.25**<br>**(0.17)**	**0.40**<br>**(0.05)**	0.11<br>(0.68)	**0.78**<br>**(0.01)**	**0.28**<br>**(0.86)**	0.07<br>(0.19)	0.06<br>(0.22)	**0.21**<br>**(0.39)**
mEP	DE phthalate	0.01<br>(1.00)	0.02<br>(1.00)	0.04<br>(1.00)	0.02<br>(1.00)	0.02<br>(1.00)	0.04<br>(1.00)	0.05<br>(1.00)	0.07<br>(1.00)	0.01<br>(1.00)	0.02<br>(1.00)	0.03<br>(1.00)	0.08<br>(1.00)
mMP	DMP	0.02<br>(1.00)	0.04<br>(1.00)	0.03<br>(1.00)	0.02<br>(1.00)	0.05<br>(1.00)	0.02<br>(1.00)	0.01<br>(1.00)	0.06<br>(1.00)	0.02<br>(1.00)	0.01<br>(1.00)	0.03<br>(1.00)	0.03<br>(1.00)
mCPP	DNOP	0.06<br>(1.00)	0.07<br>(1.00)	0.02<br>(1.00)	0.02<br>(1.00)	0.02<br>(1.00)	0.10<br>(1.00)	0.02<br>(1.00)	0.03<br>(1.00)	0.07<br>(1.00)	0.01<br>(1.00)	0.03<br>(1.00)	0.02<br>(1.00)
BPA	BPA	0.01<br>(1.00)	0.05<br>(1.00)	0.02<br>(1.00)	0.02<br>(1.00)	0.02<br>(1.00)	0.03<br>(1.00)	0.03<br>(1.00)	0.01<br>(1.00)	0.05<br>(1.00)	0.01<br>(1.00)	0.03<br>(1.00)	0.02<br>(1.00)
DEP	DAP	**0.25**<br>**(0.02)**	**0.23**<br>**(0.03)**	0.07<br>(0.11)	0.05<br>(0.07)	0.04<br>(0.11)	0.09<br>(0.46)	0.06<br>(0.36)	0.04<br>(0.38)	0.08<br>(0.46)	0.02<br>(0.26)	0.04<br>(0.26)	0.05<br>(0.28)
DETP	DAP	**0.25**<br>**(0.13)**	**0.23**<br>**(0.06)**	0.07<br>(0.14)	0.05<br>(0.30)	0.04<br>(0.43)	0.09<br>(0.13)	0.06<br>(0.14)	0.04<br>(0.18)	0.08<br>(0.11)	0.02<br>(0.25)	0.04<br>(0.15)	0.05<br>(0.29)
DMDTP	DAP	**0.25**<br>**(0.03)**	**0.23**<br>**(0.08)**	0.07<br>(0.25)	0.05<br>(0.09)	0.04<br>(0.11)	0.09<br>(0.09)	0.06<br>(0.07)	0.04<br>(0.13)	0.08<br>(0.09)	0.02<br>(0.22)	0.04<br>(0.16)	0.05<br>(0.22)
DMP	DAP	**0.25**<br>**(0.12)**	**0.23**<br>**(0.05)**	0.07<br>(0.25)	0.05<br>(0.37)	0.04<br>(0.16)	0.09<br>(0.23)	0.06<br>(0.19)	0.04<br>(0.12)	0.08<br>(0.17)	0.02<br>(0.15)	0.04<br>(0.14)	0.05<br>(0.15)

DMTP	DAP	**0.25 (0.71)**	**0.23 (0.78)**	0.07 (0.25)	0.05 (0.17)	0.04 (0.20)	0.09 (0.10)	0.06 (0.22)	0.04 (0.19)	0.08 (0.17)	0.02 (0.12)	0.04 (0.29)	0.05 (0.06)

Abbreviations: monobenzyl phthalate, mBzP; butyl benzyl phthalate, BBzP; mono-[(2-carboxymethyl)hexyl] phthalate, mCMHP; mono-(2-ethyl-5-carboxypentyl) phthalate, mECPP; mono-(2-ethyl-5-hydroxyhexyl) phthalate, mEHHP; mono-(2-ethyl-5-oxohexyl) phthalate, mEOHP; di(2-ethyhexyl) phthalate, DEHP; monobutyl phthalate, mBP; monoisobutyl phthalate, mIBP; dibutyl phthalate DBP; monoethyl phthalate, mEP; diethyl phthalate, DE phthalate; monomethyl phthalate, mMP; dimethyl phthalate, DMP; mono(3-carboxypropyl) phthalate, mCPP; di-n-octyl phthalate, DNOP; bisphenol A, BPA; diethyl phosphate, DEP; diethylthiophosphate, DETP; dimethyldithiophosphate, DMDTP; dimethylphosphate, DMP; dimethylthiophosphate, DMTP; dialkylphosphate, DAP. Values are presented as group posterior inclusion probability (PIP) with conditional PIP shown in parentheses. Group PIPs above 0.20 in bold. Within each group, all conditional PIPs sum to 1. The group PIP reflects the relative importance of exposure biomarker group to the mixture–outcome association, and the conditional PIP reflects the relative importance of each metabolite component within the group.

Table S 7. Posterior inclusion probability of childhood chemicals exposure obtained using BKMR with the full set of chemicals

		Verbal comprehension			Matrix reasoning			Digit span			Coding		
Chemicals	Group	All	Boys	Girls	All	Boys	Girls	All	Boys	Girls	All	Boys	Girls
mCMHP	DEHP	0.02	0.07	0.04	0.05	0.05	0.05	0.03	0.05	0.09	0.05	0.03	**0.22**
		(0.21)	(0.16)	(0.21)	(0.18)	(0.31)	(0.19)	(0.23)	(0.17)	(0.31)	(0.48)	(0.26)	**(0.63)**
mECPP	DEHP	0.02	0.07	0.04	0.05	0.05	0.05	0.03	0.05	0.09	0.05	0.03	**0.22**
		(0.22)	(0.40)	(0.16)	(0.53)	(0.26)	(0.38)	(0.10)	(0.34)	(0.07)	(0.16)	(0.26)	**(0.07)**
mEHHP	DEHP	0.02	0.07	0.04	0.05	0.05	0.05	0.03	0.05	0.09	0.05	0.03	**0.22**
		(0.26)	(0.20)	(0.27)	(0.13)	(0.19)	(0.25)	(0.27)	(0.22)	(0.28)	(0.15)	(0.25)	**(0.15)**
mEOHP	DEHP	0.02	0.07	0.04	0.05	0.05	0.05	0.03	0.05	0.09	0.05	0.03	**0.22**
		(0.31)	(0.24)	(0.36)	(0.16)	(0.24)	(0.19)	(0.39)	(0.26)	(0.33)	(0.20)	(0.23)	**(0.16)**
mBP	DBP	**0.33**	0.11	0.14	0.01	0.02	0.04	**0.46**	0.11	**0.52**	0.01	0.05	0.03
		**(0.99)**	(0.91)	(0.91)	(0.67)	(0.55)	(0.63)	**(0.01)**	(0.11)	**(0.02)**	(0.50)	(0.67)	(0.45)
mIBP	DBP	**0.33**	0.11	0.14	0.01	0.02	0.04	**0.46**	0.11	**0.52**	0.01	0.05	0.03
		**(0.01)**	(0.09)	(0.09)	(0.33)	(0.45)	(0.37)	**(0.99)**	(0.89)	**(0.98)**	(0.50)	(0.33)	(0.55)
mEP	DE phthalate	0.01	0.05	0.03	0.01	0.03	0.02	0.15	0.10	0.20	0.03	0.11	0.02
		(1.00)	(1.00)	(1.00)	(1.00)	(1.00)	(1.00)	(1.00)	(1.00)	(1.00)	(1.00)	(1.00)	(1.00)
mMP	DMP	0.15	0.05	0.05	0.04	0.15	0.02	0.01	0.04	0.06	0.01	0.03	0.02
		(1.00)	(1.00)	(1.00)	(1.00)	(1.00)	(1.00)	(1.00)	(1.00)	(1.00)	(1.00)	(1.00)	(1.00)
mCPP	DNOP	0.01	0.02	0.03	0.01	0.02	0.06	0.16	0.05	0.23	0.01	0.02	0.01
		(1.00)	(1.00)	(1.00)	(1.00)	(1.00)	(1.00)	(1.00)	(1.00)	(1.00)	(1.00)	(1.00)	(1.00)
BPA	BPA	0.03	0.04	0.03	0.01	0.04	0.02	0.02	0.02	0.21	0.02	0.02	0.01
		(1.00)	(1.00)	(1.00)	(1.00)	(1.00)	(1.00)	(1.00)	(1.00)	(1.00)	(1.00)	(1.00)	(1.00)
DEP	DAP	**0.30**	0.08	**0.29**	0.04	0.06	0.06	0.03	0.10	0.10	0.05	**0.37**	0.08
		**(0.03)**	(0.13)	**(0.27)**	(0.23)	(0.23)	(0.25)	(0.37)	(0.51)	(0.55)	(0.34)	**(0.09)**	(0.18)
DETP	DAP	**0.30**	0.08	**0.29**	0.04	0.06	0.06	0.03	0.10	0.10	0.05	**0.37**	0.08
		**(0.02)**	(0.16)	**(0.09)**	(0.45)	(0.42)	(0.28)	(0.33)	(0.16)	(0.28)	(0.33)	**(0.33)**	(0.12)
DMP	DAP	**0.30**	0.08	**0.29**	0.04	0.06	0.06	0.03	0.10	0.10	0.05	**0.37**	0.08
		**(0.80)**	(0.50)	**(0.48)**	(0.16)	(0.17)	(0.26)	(0.14)	(0.16)	(0.09)	(0.10)	**(0.42)**	(0.60)
DMTP	DAP	**0.30**	0.08	**0.29**	0.04	0.06	0.06	0.03	0.10	0.10	0.05	**0.37**	0.08
		**(0.14)**	(0.21)	**(0.16)**	(0.16)	(0.18)	(0.21)	(0.16)	(0.17)	(0.07)	(0.22)	**(0.15)**	(0.10)

Abbreviations: mono-[(2-carboxymethyl)hexyl] phthalate, mCMHP; mono-(2-ethyl-5-carboxypentyl) phthalate, mECPP; mono-(2-ethyl-5-hydroxyhexyl) phthalate, mEHHP; mono-(2-ethyl-5-oxohexyl) phthalate, mEOHP; di(2-ethyhexyl) phthalate, DEHP;

monobutyl phthalate, mBP; monoisobutyl phthalate, mIBP; dibutyl phthalate DBP; monoethyl phthalate, mEP; diethyl phthalate, DE phthalate; monomethyl phthalate, mMP; dimethyl phthalate, DMP; mono(3-carboxypropyl) phthalate, mCPP; di-n-octyl phthalate, DNOP; bisphenol A, BPA; diethyl phosphate, DEP; diethylthiophosphate, DETP; dimethylphosphate, DMP; dimethylthiophosphate, DMTP; dialkylphosphate, DAP.
Values are presented as group posterior inclusion probability (PIP), with conditional PIP shown in parentheses. Group PIPs above 0.20 in bold. Within each group, all conditional PIPs sum to 1. The group PIP reflects the relative importance of exposure biomarker group to the mixture–outcome association, and the conditional PIP reflects the relative importance of each metabolite component within the group.

Table S 8. Posterior inclusion probability of prenatal chemicals exposure obtained using hBKMR with selected chemicals

		Verbal comprehension			Matrix reasoning			Digit span			Coding		
Chemicals	Group	All	Boys	Girls	All	Boys	Girls	All	Boys	Girls	All	Boys	Girls
mCMHP	DEHP	0.06 (0.13)	0.28 (0.19)	0.09 (0.38)	0.09 (0.16)	**0.61 (0.13)**	0.19 (0.23)	0.08 (0.11)	0.20 (0.14)	0.07 (0.35)	**0.68 (0.90)**	**0.57 (0.08)**	**0.54 (0.30)**
mECPP	DEHP	0.06 (0.38)	0.28 (0.51)	0.09 (0.20)	0.09 (0.34)	**0.61 (0.48)**	0.19 (0.10)	0.08 (0.31)	0.20 (0.28)	0.07 (0.17)	**0.68 (0.01)**	**0.57 (0.33)**	**0.54 (0.27)**
mEHHP	DEHP	0.06 (0.28)	0.28 (0.18)	0.09 (0.17)	0.09 (0.30)	**0.61 (0.25)**	0.19 (0.21)	0.08 (0.24)	0.20 (0.36)	0.07 (0.21)	**0.68 (0.07)**	**0.57 (0.15)**	**0.54 (0.34)**
mEOHP	DEHP	0.06 (0.22)	0.28 (0.12)	0.09 (0.25)	0.09 (0.20)	**0.61 (0.14)**	0.19 (0.47)	0.08 (0.35)	0.20 (0.22)	0.07 (0.27)	**0.68 (0.02)**	**0.57 (0.44)**	**0.54 (0.09)**
mBP	DBP	0.13 (0.79)	0.30 (0.86)	0.09 (0.44)	0.35 (0.49)	0.31 (0.82)	**0.74 (0.97)**	0.25 (0.32)	**0.87 (0.99)**	**0.55 (0.15)**	0.12 (0.83)	0.12 (0.73)	0.33 (0.68)
mIBP	DBP	0.13 (0.21)	0.30 (0.14)	0.09 (0.56)	0.35 (0.51)	0.31 (0.18)	**0.74 (0.03)**	0.25 (0.68)	**0.87 (0.01)**	**0.55 (0.85)**	0.12 (0.17)	0.12 (0.27)	0.33 (0.32)
DEP	DAP	0.45 (0.02)	0.40 (0.02)	0.17 (0.10)	0.11 (0.06)	0.12 (0.16)	0.18 (0.47)	0.13 (0.36)	0.09 (0.42)	0.14 (0.49)	0.04 (0.34)	0.08 (0.29)	0.10 (0.28)
DETP	DAP	0.45 (0.13)	0.40 (0.06)	0.17 (0.15)	0.11 (0.26)	0.12 (0.36)	0.18 (0.12)	0.13 (0.14)	0.09 (0.17)	0.14 (0.09)	0.04 (0.20)	0.08 (0.15)	0.10 (0.24)
DMDTP	DAP	0.45 (0.03)	0.40 (0.09)	0.17 (0.23)	0.11 (0.09)	0.12 (0.12)	0.18 (0.07)	0.13 (0.08)	0.09 (0.10)	0.14 (0.14)	0.04 (0.22)	0.08 (0.14)	0.10 (0.21)
DMP	DAP	0.45 (0.10)	0.40 (0.04)	0.17 (0.26)	0.11 (0.40)	0.12 (0.19)	0.18 (0.26)	0.13 (0.24)	0.09 (0.12)	0.14 (0.18)	0.04 (0.13)	0.08 (0.12)	0.10 (0.18)
DMTP	DAP	0.45 (0.72)	0.40 (0.79)	0.17 (0.26)	0.11 (0.18)	0.12 (0.17)	0.18 (0.07)	0.13 (0.18)	0.09 (0.20)	0.14 (0.10)	0.04 (0.10)	0.08 (0.30)	0.10 (0.09)

Abbreviations: mono-[(2-carboxymethyl)hexyl] phthalate, mCMHP; mono-(2-ethyl-5-carboxypentyl) phthalate, mECPP; mono-(2-ethyl-5-hydroxyhexyl) phthalate, mEHHP; mono-(2-ethyl-5-oxohexyl) phthalate, mEOHP; di(2-ethyhexyl) phthalate, DEHP; monobutyl phthalate, mBP; monoisobutyl phthalate, mIBP; dibutyl phthalate DBP; diethyl phosphate, DEP; diethylthiophosphate, DETP; dimethyldithiophosphate, DMDTP; dimethylphosphate, DMP; dimethylthiophosphate, DMTP; dialkylphosphate, DAP.
Values are presented as group posterior inclusion probability (PIP) with conditional PIP shown in parentheses. Group PIPs above 0.50 in bold. Within each group, all conditional PIPs sum to 1. The group PIP reflects the relative importance of exposure biomarker group to the mixture–outcome association, and the conditional PIP reflects the relative importance of each metabolite component within the group.

Table S 9. Single-chemical associations of individual chemical exposure during pregnancy with cognitive performance subtest scores

Metabolites	Parent compounds	Verbal comprehension	Matrix reasoning	Digit span	Coding
Averaged pregnancy					
mBzP	BBzP	-0.13 (-0.36, 0.1)	0.11 (-0.1, 0.32)	0.17 (-0.05, 0.39)	-0.01 (-0.28, 0.25)
Girls		-0.10 (-0.45, 0.24)	0.16 (-0.15, 0.47)	**0.31 (0.01, 0.6)**	-0.07 (-0.45, 0.31)
Boys		-0.12 (-0.44, 0.2)	0.02 (-0.27, 0.32)	0.03 (-0.31, 0.37)	0.07 (-0.31, 0.44)
mCMHP	DEHP	-0.02 (-0.25, 0.2)	-0.08 (-0.29, 0.14)	-0.02 (-0.24, 0.2)	-0.26 (-0.52, 0.01)
Girls		0.20 (-0.14, 0.54)	0.07 (-0.23, 0.38)	0.19 (-0.1, 0.48)	-0.17 (-0.54, 0.2)
Boys		-0.23 (-0.55, 0.1)	-0.25 (-0.55, 0.04)	-0.24 (-0.57, 0.1)	-0.36 (-0.73, 0.02)
mECPP	DEHP	-0.13 (-0.36, 0.1)	-0.18 (-0.39, 0.03)	-0.10 (-0.32, 0.12)	-0.08 (-0.35, 0.18)
Girls		0.10 (-0.24, 0.44)	0.06 (-0.24, 0.37)	0.07 (-0.23, 0.36)	0.03 (-0.34, 0.41)
Boys		-0.35 (-0.67, -0.04)	**-0.45 (-0.74, -0.15)**	-0.27 (-0.61, 0.07)	-0.19 (-0.57, 0.19)
mEHHP	DEHP	-0.11 (-0.34, 0.12)	-0.16 (-0.37, 0.05)	-0.13 (-0.35, 0.09)	-0.24 (-0.5, 0.02)
Girls		0.05 (-0.29, 0.39)	0.04 (-0.27, 0.34)	0.07 (-0.22, 0.36)	-0.20 (-0.57, 0.18)
Boys		-0.28 (-0.6, 0.05)	**-0.40 (-0.69, -0.1)**	**-0.34 (-0.68, 0)**	-0.32 (-0.7, 0.05)
mEOHP	DEHP	-0.01 (-0.24, 0.22)	-0.13 (-0.34, 0.08)	-0.11 (-0.33, 0.11)	-0.23 (-0.49, 0.03)
Girls		0.17 (-0.17, 0.51)	0.07 (-0.23, 0.37)	0.07 (-0.22, 0.36)	-0.15 (-0.53, 0.22)
Boys		-0.16 (-0.48, 0.17)	**-0.38 (-0.67, -0.08)**	-0.31 (-0.65, 0.03)	-0.32 (-0.7, 0.05)
mBP	DBP	-0.22 (-0.45, 0.02)	**-0.22 (-0.43, -0.01)**	-0.14 (-0.36, 0.09)	**-0.33 (-0.6, -0.06)**
Girls		-0.04 (-0.38, 0.31)	-0.17 (-0.48, 0.14)	0.01 (-0.28, 0.31)	**-0.46 (-0.84, -0.08)**
Boys		**-0.36 (-0.69, -0.03)**	**-0.33 (-0.63, -0.03)**	-0.30 (-0.64, 0.05)	-0.28 (-0.66, 0.1)
mIBP	DBP	-0.13 (-0.36, 0.1)	-0.19 (-0.4, 0.03)	0.18 (-0.04, 0.4)	-0.17 (-0.44, 0.09)
Girls		-0.02 (-0.37, 0.32)	-0.15 (-0.46, 0.17)	0.27 (-0.03, 0.57)	-0.38 (-0.76, 0)
Boys		-0.22 (-0.55, 0.11)	-0.27 (-0.56, 0.03)	0.09 (-0.25, 0.43)	0.00 (-0.37, 0.38)
mEP	DE phthalate	0.07 (-0.16, 0.31)	-0.12 (-0.33, 0.1)	-0.13 (-0.35, 0.09)	0.11 (-0.15, 0.38)
Girls		0.03 (-0.32, 0.37)	-0.14 (-0.46, 0.17)	0.03 (-0.27, 0.33)	**0.40 (0.02, 0.78)**
Boys		0.15 (-0.18, 0.47)	-0.10 (-0.39, 0.2)	-0.26 (-0.6, 0.08)	-0.19 (-0.57, 0.18)
mMP	DMP	0.01 (-0.22, 0.24)	0.06 (-0.15, 0.27)	0.05 (-0.17, 0.27)	-0.05 (-0.31, 0.22)
Girls		-0.10 (-0.44, 0.24)	0.11 (-0.2, 0.41)	-0.10 (-0.39, 0.19)	-0.29 (-0.66, 0.08)
Boys		0.10 (-0.22, 0.43)	0.00 (-0.3, 0.3)	0.18 (-0.16, 0.52)	0.16 (-0.21, 0.54)

mCPP	DNOP	-0.23 (-0.45, 0)	0.10 (-0.11, 0.31)	0.11 (-0.11, 0.33)	-0.19 (-0.45, 0.08)
Girls		-0.14 (-0.47, 0.2)	0.22 (-0.08, 0.52)	**0.29 (0, 0.57)**	-0.14 (-0.52, 0.23)
Boys		-0.28 (-0.61, 0.04)	-0.08 (-0.38, 0.22)	-0.07 (-0.41, 0.27)	-0.22 (-0.6, 0.16)
BPA	BPA	0.01 (-0.22, 0.24)	-0.04 (-0.25, 0.17)	0.05 (-0.17, 0.27)	0.00 (-0.26, 0.26)
Girls		0.08 (-0.26, 0.42)	0.05 (-0.26, 0.35)	0.15 (-0.13, 0.44)	0.16 (-0.21, 0.54)
Boys		-0.04 (-0.37, 0.29)	-0.21 (-0.5, 0.09)	-0.05 (-0.39, 0.29)	-0.18 (-0.56, 0.2)
DEP	DAP	-0.12 (-0.36, 0.11)	-0.04 (-0.26, 0.18)	-0.13 (-0.36, 0.09)	0.08 (-0.19, 0.35)
Girls		-0.10 (-0.44, 0.25)	-0.15 (-0.46, 0.15)	-0.26 (-0.55, 0.03)	0.02 (-0.36, 0.4)
Boys		-0.13 (-0.48, 0.21)	0.03 (-0.28, 0.35)	0.01 (-0.35, 0.37)	0.18 (-0.22, 0.58)
DETP	DAP	-0.18 (-0.41, 0.06)	-0.11 (-0.32, 0.11)	-0.17 (-0.4, 0.05)	-0.09 (-0.36, 0.17)
Girls		-0.16 (-0.5, 0.17)	-0.16 (-0.47, 0.14)	-0.15 (-0.44, 0.13)	-0.12 (-0.49, 0.25)
Boys		-0.19 (-0.53, 0.16)	-0.03 (-0.35, 0.29)	-0.22 (-0.58, 0.15)	-0.13 (-0.54, 0.27)
DMDTP	DAP	-0.16 (-0.39, 0.07)	-0.06 (-0.28, 0.15)	-0.12 (-0.34, 0.11)	0.02 (-0.24, 0.29)
Girls		-0.03 (-0.37, 0.31)	-0.15 (-0.46, 0.15)	-0.10 (-0.39, 0.2)	-0.03 (-0.4, 0.34)
Boys		-0.29 (-0.63, 0.04)	0.02 (-0.29, 0.33)	-0.15 (-0.51, 0.2)	-0.01 (-0.4, 0.39)
DMP	DAP	-0.22 (-0.45, 0.01)	-0.13 (-0.34, 0.09)	-0.19 (-0.42, 0.03)	0.08 (-0.19, 0.34)
Girls		-0.19 (-0.53, 0.16)	-0.10 (-0.41, 0.21)	-0.19 (-0.49, 0.1)	0.21 (-0.17, 0.59)
Boys		-0.23 (-0.56, 0.09)	-0.19 (-0.49, 0.11)	-0.23 (-0.58, 0.11)	-0.06 (-0.44, 0.32)
DMTP	DAP	**-0.35 (-0.58, -0.11)**	-0.15 (-0.37, 0.06)	-0.20 (-0.43, 0.02)	-0.05 (-0.32, 0.21)
Girls		-0.22 (-0.56, 0.12)	-0.11 (-0.41, 0.2)	-0.14 (-0.43, 0.15)	0.00 (-0.37, 0.38)
Boys		**-0.48 (-0.81, -0.14)**	-0.23 (-0.54, 0.08)	-0.30 (-0.65, 0.06)	-0.21 (-0.61, 0.18)

Abbreviations: monobenzyl phthalate, mBzP; butyl benzyl phthalate, BBzP; mono-[(2-carboxymethyl)hexyl] phthalate, mCMHP; mono-(2-ethyl-5-carboxypentyl) phthalate, mECPP; mono-(2-ethyl-5-hydroxyhexyl) phthalate, mEHHP; mono-(2-ethyl-5-oxohexyl) phthalate, mEOHP; di(2-ethyhexyl) phthalate, DEHP; monobutyl phthalate, mBP; monoisobutyl phthalate, mIBP; dibutyl phthalate DBP; monoethyl phthalate, mEP; diethyl phthalate, DE phthalate; monomethyl phthalate, mMP; dimethyl phthalate, DMP; mono(3-carboxypropyl) phthalate, mCPP; di-n-octyl phthalate, DNOP; bisphenol A, BPA; diethyl phosphate, DEP; diethylthiophosphate, DETP; dimethyldithiophosphate, DMDTP; dimethylphosphate, DMP; dimethylthiophosphate, DMTP; dialkylphosphate, DAP.
Concentrations of exposure biomarkers were log2 transformed and standardized into z-scores. All models were adjusted for child age at cognitive functioning assessment, child sex, child migration background, maternal age, household income, maternal education, parity, maternal smoking during pregnancy, maternal pre-pregnancy BMI, and folic acid supplement use. The model of verbal score was additionally adjusted for maternal verbal IQ, and the models of other subtest scores were additionally adjusted for maternal non-verbal IQ.

Table S 10. Posterior inclusion probability of childhood chemicals exposure obtained using hBKMR with selected chemicals

		Verbal comprehension			Matrix reasoning			Digit span			Coding		
Chemicals	Group	All	Boys	Girls	All	Boys	Girls	All	Boys	Girls	All	Boys	Girls
mCMHP	DEHP	0.05 (0.24)	0.15 (0.17)	0.09 (0.18)	0.10 (0.19)	0.12 (0.31)	0.11 (0.19)	0.08 (0.21)	0.12 (0.18)	0.20 (0.32)	0.09 (0.47)	0.06 (0.23)	0.41 (0.62)
mECPP	DEHP	0.05 (0.19)	0.15 (0.36)	0.09 (0.15)	0.10 (0.57)	0.12 (0.29)	0.11 (0.36)	0.08 (0.09)	0.12 (0.33)	0.20 (0.07)	0.09 (0.17)	0.06 (0.23)	0.41 (0.07)
mEHHP	DEHP	0.05 (0.26)	0.15 (0.21)	0.09 (0.25)	0.10 (0.12)	0.12 (0.17)	0.11 (0.25)	0.08 (0.26)	0.12 (0.21)	0.20 (0.26)	0.09 (0.16)	0.06 (0.27)	0.41 (0.15)
mEOHP	DEHP	0.05 (0.31)	0.15 (0.26)	0.09 (0.42)	0.10 (0.12)	0.12 (0.23)	0.11 (0.20)	0.08 (0.43)	0.12 (0.28)	0.20 (0.35)	0.09 (0.19)	0.06 (0.26)	0.41 (0.16)
mBP	DBP	0.47 (0.99)	0.22 (0.91)	0.27 (0.90)	0.03 (0.65)	0.06 (0.55)	0.09 (0.64)	**0.65 (0.01)**	0.24 (0.11)	**0.64 (0.02)**	0.03 (0.42)	0.10 (0.66)	0.06 (0.46)
mIBP	DBP	0.47 (0.01)	0.22 (0.09)	0.27 (0.10)	0.03 (0.35)	0.06 (0.45)	0.09 (0.36)	**0.65 (0.99)**	0.24 (0.89)	**0.64 (0.98)**	0.03 (0.58)	0.10 (0.34)	0.06 (0.54)
DEP	DAP	**0.51 (0.03)**	0.16 (0.12)	0.47 (0.28)	0.09 (0.24)	0.12 (0.22)	0.14 (0.25)	0.07 (0.31)	0.20 (0.48)	0.15 (0.53)	0.13 (0.35)	**0.55 (0.11)**	0.14 (0.19)
DETP	DAP	**0.51 (0.02)**	0.16 (0.16)	0.47 (0.08)	0.09 (0.49)	0.12 (0.42)	0.14 (0.29)	0.07 (0.38)	0.20 (0.19)	0.15 (0.29)	0.13 (0.36)	**0.55 (0.36)**	0.14 (0.16)
DMP	DAP	**0.51 (0.80)**	0.16 (0.51)	0.47 (0.46)	0.09 (0.14)	0.12 (0.17)	0.14 (0.27)	0.07 (0.15)	0.20 (0.15)	0.15 (0.09)	0.13 (0.09)	**0.55 (0.36)**	0.14 (0.56)
DMTP	DAP	**0.51 (0.15)**	0.16 (0.22)	0.47 (0.18)	0.09 (0.13)	0.12 (0.19)	0.14 (0.19)	0.07 (0.16)	0.20 (0.18)	0.15 (0.08)	0.13 (0.21)	**0.55 (0.17)**	0.14 (0.09)

Abbreviations: mono-[(2-carboxymethyl)hexyl] phthalate, mCMHP; mono-(2-ethyl-5-carboxypentyl) phthalate, mECPP; mono-(2-ethyl-5-hydroxyhexyl) phthalate, mEHHP; mono-(2-ethyl-5-oxohexyl) phthalate, mEOHP; di(2-ethyhexyl) phthalate, DEHP; monobutyl phthalate, mBP; monoisobutyl phthalate, mIBP; dibutyl phthalate DBP; diethyl phosphate, DEP; diethylthiophosphate, DETP; dimethylphosphate, DMP; dimethylthiophosphate, DMTP; dialkylphosphate, DAP.

Values are presented as group posterior inclusion probability (PIP) with conditional PIP shown in parentheses. Group PIPs above 0.50 in bold. Within each group, all conditional PIPs sum to 1. The group PIP reflects the relative importance of exposure biomarker group to the mixture–outcome association, and the conditional PIP reflects the relative importance of each metabolite component within the group.

Table S 11. Single-chemical associations of individual chemical exposure in childhood with cognitive performance subtest scores

Metabolites	Parent compounds	Verbal comprehension	Matrix reasoning	Digit span	Coding
Childhood					
mCMHP	DEHP	0.05 (-0.19, 0.28)	-0.03 (-0.24, 0.19)	-0.06 (-0.29, 0.17)	-0.26 (-0.53, 0.02)
Girls		0.06 (-0.29, 0.4)	0.07 (-0.23, 0.38)	-0.11 (-0.42, 0.2)	**-0.50 (-0.89, -0.11)**
Boys		0.05 (-0.29, 0.39)	-0.10 (-0.42, 0.21)	-0.02 (-0.38, 0.34)	-0.05 (-0.44, 0.34)
mECPP	DEHP	0.11 (-0.12, 0.35)	0.13 (-0.08, 0.35)	0.03 (-0.2, 0.26)	-0.06 (-0.34, 0.21)
Girls		-0.01 (-0.35, 0.34)	0.18 (-0.13, 0.48)	-0.03 (-0.34, 0.28)	-0.11 (-0.5, 0.29)
Boys		0.23 (-0.1, 0.56)	0.07 (-0.23, 0.38)	0.06 (-0.29, 0.42)	-0.04 (-0.43, 0.34)
mEHHP	DEHP	0.11 (-0.12, 0.34)	0.05 (-0.16, 0.27)	-0.01 (-0.24, 0.22)	-0.07 (-0.34, 0.21)
Girls		0.08 (-0.27, 0.42)	0.07 (-0.24, 0.38)	-0.03 (-0.34, 0.28)	-0.13 (-0.52, 0.27)
Boys		0.14 (-0.19, 0.48)	0.04 (-0.27, 0.34)	-0.01 (-0.36, 0.34)	0.02 (-0.36, 0.41)
mEOHP	DEHP	0.11 (-0.12, 0.35)	0.08 (-0.13, 0.29)	-0.02 (-0.25, 0.21)	-0.10 (-0.37, 0.17)
Girls		0.03 (-0.31, 0.38)	0.05 (-0.26, 0.36)	-0.08 (-0.4, 0.23)	-0.15 (-0.55, 0.24)
Boys		0.19 (-0.14, 0.53)	0.11 (-0.2, 0.42)	0.02 (-0.33, 0.38)	-0.02 (-0.41, 0.36)
mBP	DBP	**0.34 (0.11, 0.56)**	-0.01 (-0.22, 0.2)	0.04 (-0.18, 0.27)	-0.11 (-0.37, 0.16)
Girls		**0.37 (0.04, 0.7)**	-0.10 (-0.4, 0.2)	0.07 (-0.24, 0.37)	0.16 (-0.23, 0.54)
Boys		0.32 (0, 0.65)	0.07 (-0.23, 0.37)	0.04 (-0.31, 0.38)	-0.31 (-0.68, 0.07)
mIBP	DBP	-0.09 (-0.32, 0.14)	0.02 (-0.2, 0.23)	**0.31 (0.07, 0.54)**	-0.03 (-0.31, 0.25)
Girls		-0.15 (-0.49, 0.2)	-0.04 (-0.34, 0.27)	0.27 (-0.04, 0.58)	-0.12 (-0.52, 0.27)
Boys		-0.05 (-0.39, 0.29)	0.06 (-0.26, 0.37)	0.33 (-0.03, 0.69)	0.12 (-0.28, 0.51)
mEP	DEP	-0.03 (-0.27, 0.22)	-0.11 (-0.33, 0.11)	-0.07 (-0.31, 0.17)	-0.17 (-0.46, 0.11)
Girls		-0.04 (-0.4, 0.32)	-0.03 (-0.35, 0.3)	-0.15 (-0.48, 0.17)	-0.11 (-0.52, 0.3)
Boys		-0.02 (-0.36, 0.32)	-0.17 (-0.49, 0.15)	0.02 (-0.34, 0.39)	-0.29 (-0.68, 0.11)
mMP	DMP	0.13 (-0.1, 0.36)	-0.15 (-0.36, 0.06)	-0.05 (-0.27, 0.18)	-0.04 (-0.31, 0.23)
Girls		0.17 (-0.17, 0.51)	-0.06 (-0.36, 0.25)	0.05 (-0.26, 0.36)	-0.05 (-0.44, 0.34)
Boys		0.07 (-0.25, 0.4)	-0.29 (-0.59, 0.01)	-0.20 (-0.54, 0.15)	0.04 (-0.34, 0.41)
mCPP	DNOP	-0.10 (-0.33, 0.13)	0.01 (-0.2, 0.23)	-0.09 (-0.32, 0.14)	-0.05 (-0.32, 0.22)
Girls		-0.16 (-0.5, 0.18)	0.01 (-0.3, 0.31)	-0.22 (-0.53, 0.08)	-0.13 (-0.52, 0.26)
Boys		-0.05 (-0.38, 0.28)	-0.01 (-0.32, 0.3)	0.03 (-0.32, 0.38)	0.04 (-0.35, 0.42)
BPA	BPA	0.08 (-0.15, 0.32)	0.06 (-0.15, 0.28)	-0.15 (-0.38, 0.09)	-0.12 (-0.39, 0.15)

Girls		0.10 (-0.25, 0.44)	-0.06 (-0.37, 0.25)	-0.28 (-0.6, 0.03)	-0.12 (-0.52, 0.28)
Boys		0.10 (-0.24, 0.44)	0.22 (-0.09, 0.53)	-0.01 (-0.36, 0.35)	-0.03 (-0.42, 0.36)
DEP	DAP	0.13 (-0.11, 0.36)	0.07 (-0.14, 0.29)	0.02 (-0.21, 0.25)	0.09 (-0.18, 0.36)
Girls		0.18 (-0.16, 0.53)	0.11 (-0.2, 0.41)	0.27 (-0.04, 0.57)	0.20 (-0.19, 0.59)
Boys		0.07 (-0.27, 0.4)	0.04 (-0.27, 0.35)	-0.22 (-0.57, 0.14)	0.04 (-0.35, 0.43)
DETP	DAP	0.09 (-0.14, 0.32)	0.19 (-0.02, 0.4)	0.04 (-0.19, 0.27)	-0.13 (-0.4, 0.14)
Girls		0.21 (-0.13, 0.55)	0.16 (-0.14, 0.47)	0.23 (-0.07, 0.54)	-0.16 (-0.55, 0.23)
Boys		-0.05 (-0.39, 0.28)	0.19 (-0.12, 0.49)	-0.13 (-0.48, 0.22)	-0.13 (-0.52, 0.25)
DMP	DAP	**0.28 (0.05, 0.52)**	0.03 (-0.19, 0.24)	-0.02 (-0.25, 0.21)	-0.09 (-0.37, 0.18)
Girls		0.30 (-0.03, 0.64)	-0.02 (-0.32, 0.28)	0.03 (-0.27, 0.33)	-0.02 (-0.4, 0.37)
Boys		0.25 (-0.09, 0.59)	0.04 (-0.28, 0.35)	-0.07 (-0.43, 0.3)	-0.14 (-0.54, 0.26)
DMTP	DAP	0.22 (-0.02, 0.45)	0.06 (-0.15, 0.27)	-0.06 (-0.29, 0.17)	-0.05 (-0.32, 0.22)
Girls		0.23 (-0.11, 0.56)	0.11 (-0.19, 0.41)	0.05 (-0.26, 0.35)	-0.04 (-0.42, 0.35)
Boys		0.18 (-0.15, 0.51)	0.00 (-0.31, 0.3)	-0.17 (-0.51, 0.18)	-0.11 (-0.5, 0.27)

Abbreviations: mono-[(2-carboxymethyl)hexyl] phthalate, mCMHP; mono-(2-ethyl-5-carboxypentyl) phthalate, mECPP; mono-(2-ethyl-5-hydroxyhexyl) phthalate, mEHHP; mono-(2-ethyl-5-oxohexyl) phthalate, mEOHP; di(2-ethyhexyl) phthalate, DEHP; monobutyl phthalate, mBP; monoisobutyl phthalate, mIBP; dibutyl phthalate DBP; monoethyl phthalate, mEP; diethyl phthalate, DE phthalate; monomethyl phthalate, mMP; dimethyl phthalate, DMP; mono(3-carboxypropyl) phthalate, mCPP; di-n-octyl phthalate, DNOP; bisphenol A, BPA; diethyl phosphate, DEP; diethylthiophosphate, DETP; dimethylphosphate, DMP; dimethylthiophosphate, DMTP; dialkylphosphate, DAP.

Concentrations of exposure biomarkers were log2 transformed and standardized into z-scores. All models were adjusted for child age at cognitive functioning assessment, child sex, child migration background, maternal age, household income, maternal education, parity, maternal smoking during pregnancy, maternal pre-pregnancy BMI, and folic acid supplement use. The model of verbal score was additionally adjusted for maternal verbal IQ, and the models of other subtest scores were additionally adjusted for maternal non-verbal IQ.

Figure S 1. Flowchart of the study population

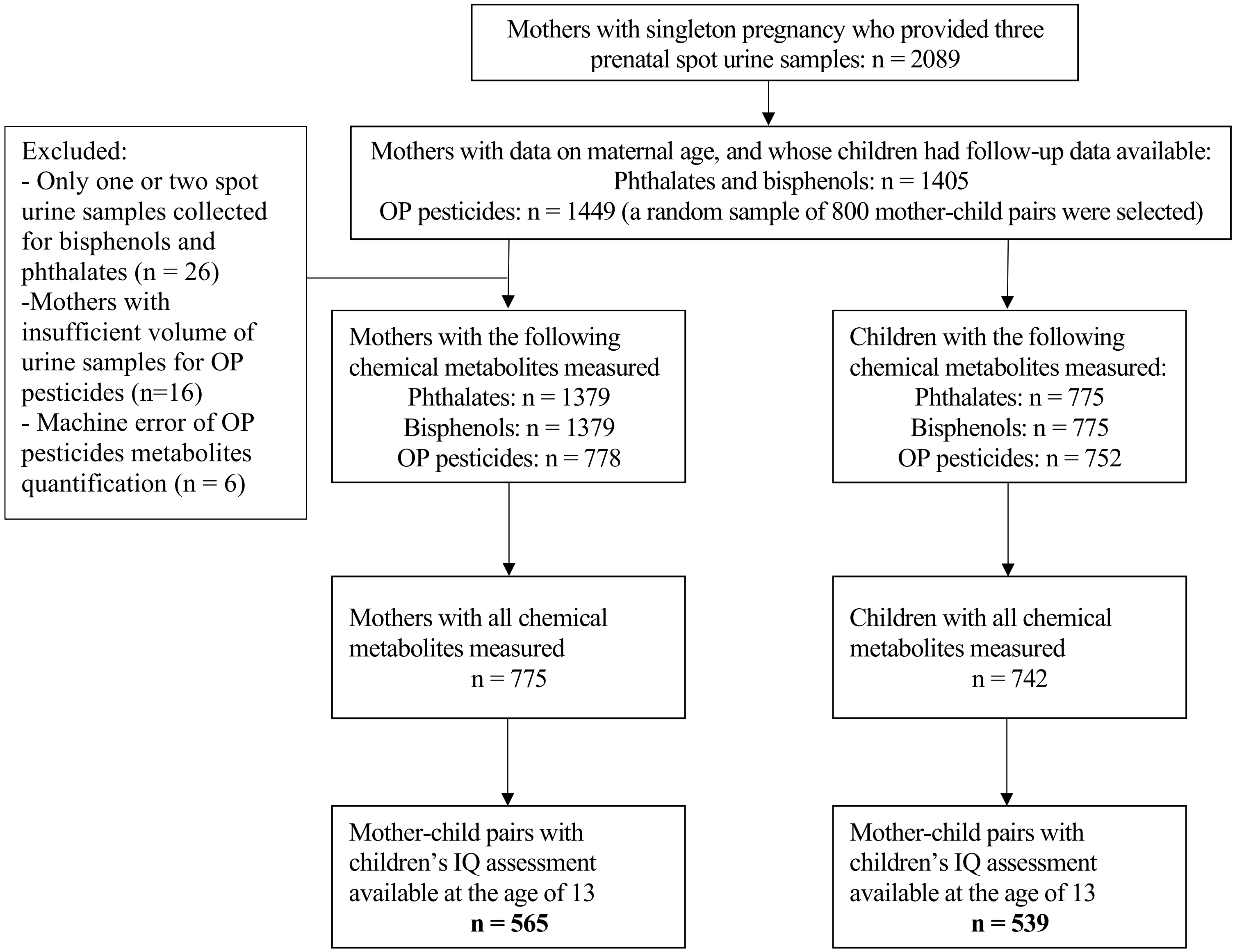

Figure S 2. Directed acyclic graphs

A) The associations between exposure to EDC mixture during pregnancy and IQ in adolescence

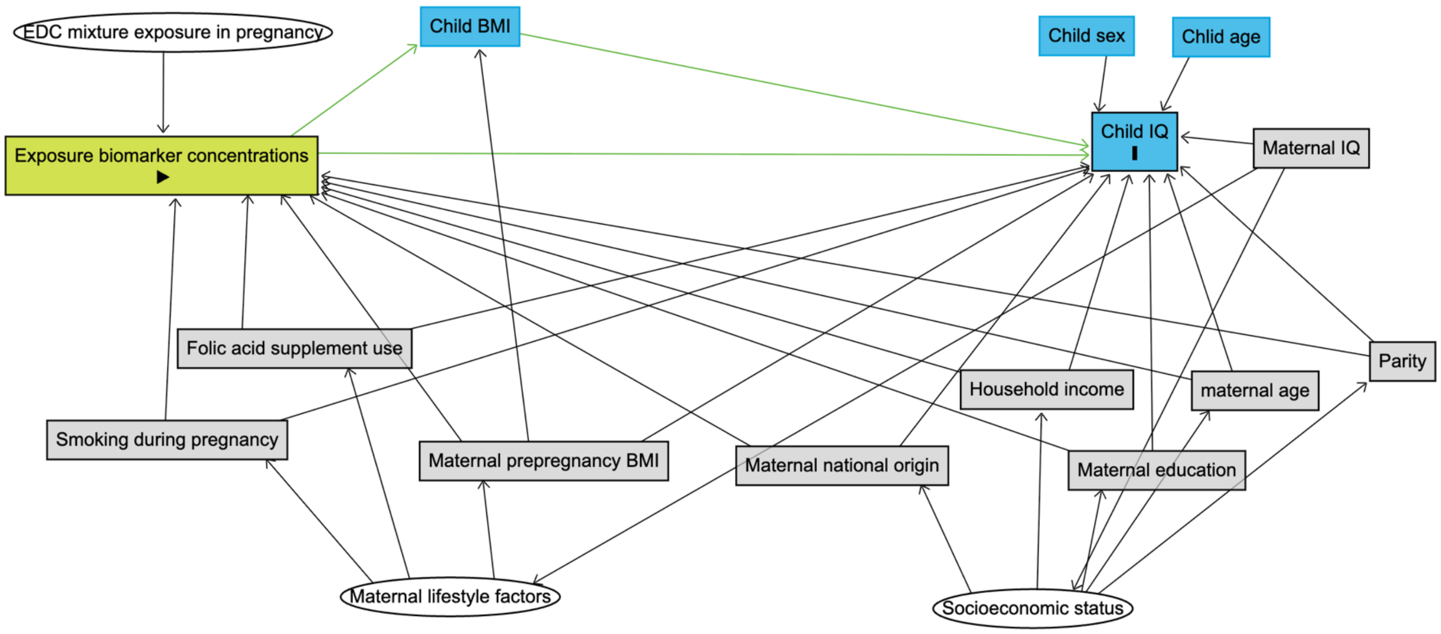

Figure S 2. Directed acyclic graphs (cont'd)

B) The associations between exposure to EDC mixture in childhood and IQ in adolescence

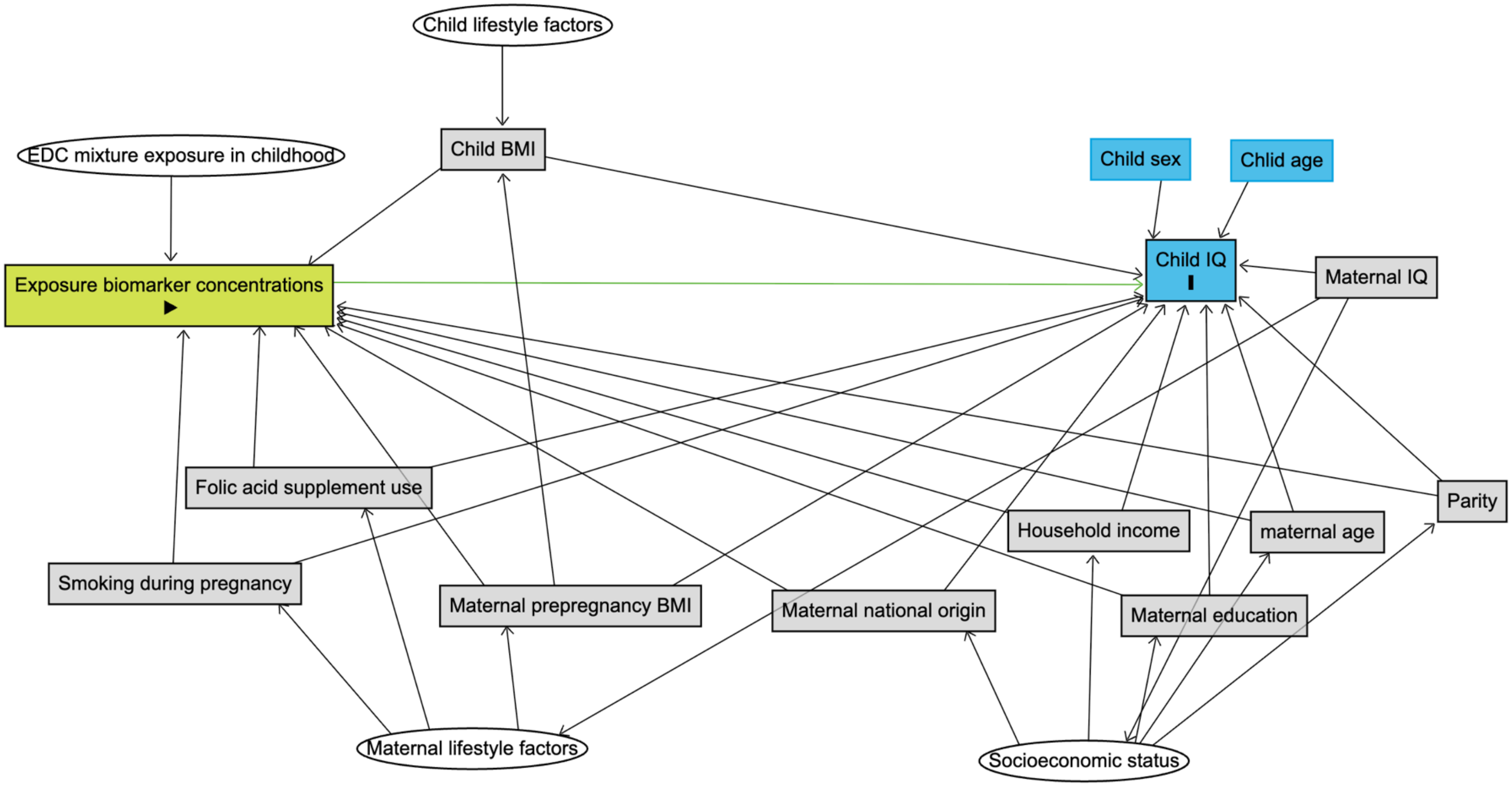

Figure S 3. Correlation matrix for prenatal exposure biomarker concentrations

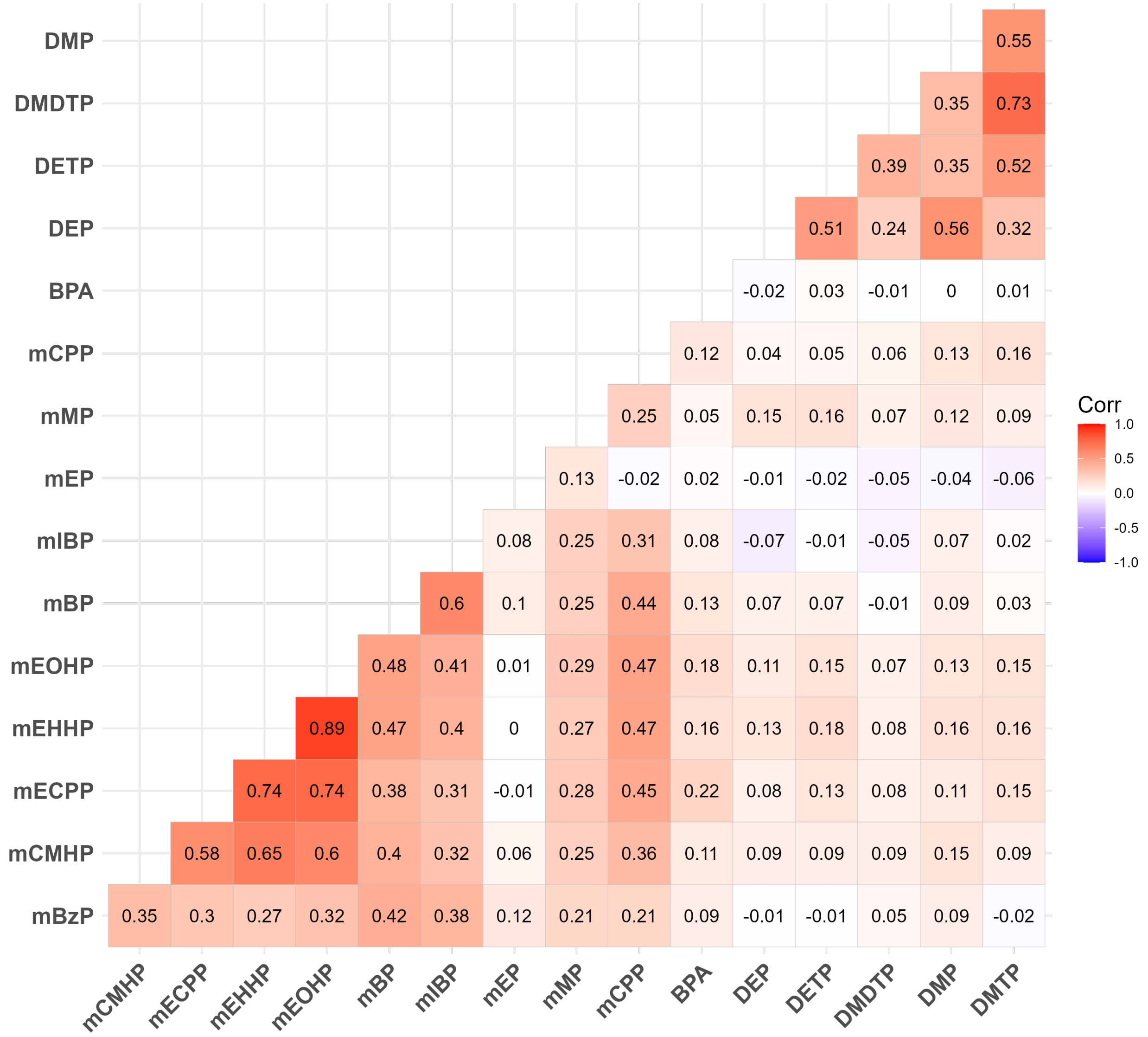

Figure S 4. Correlation matrix for childhood exposure biomarker concentrations

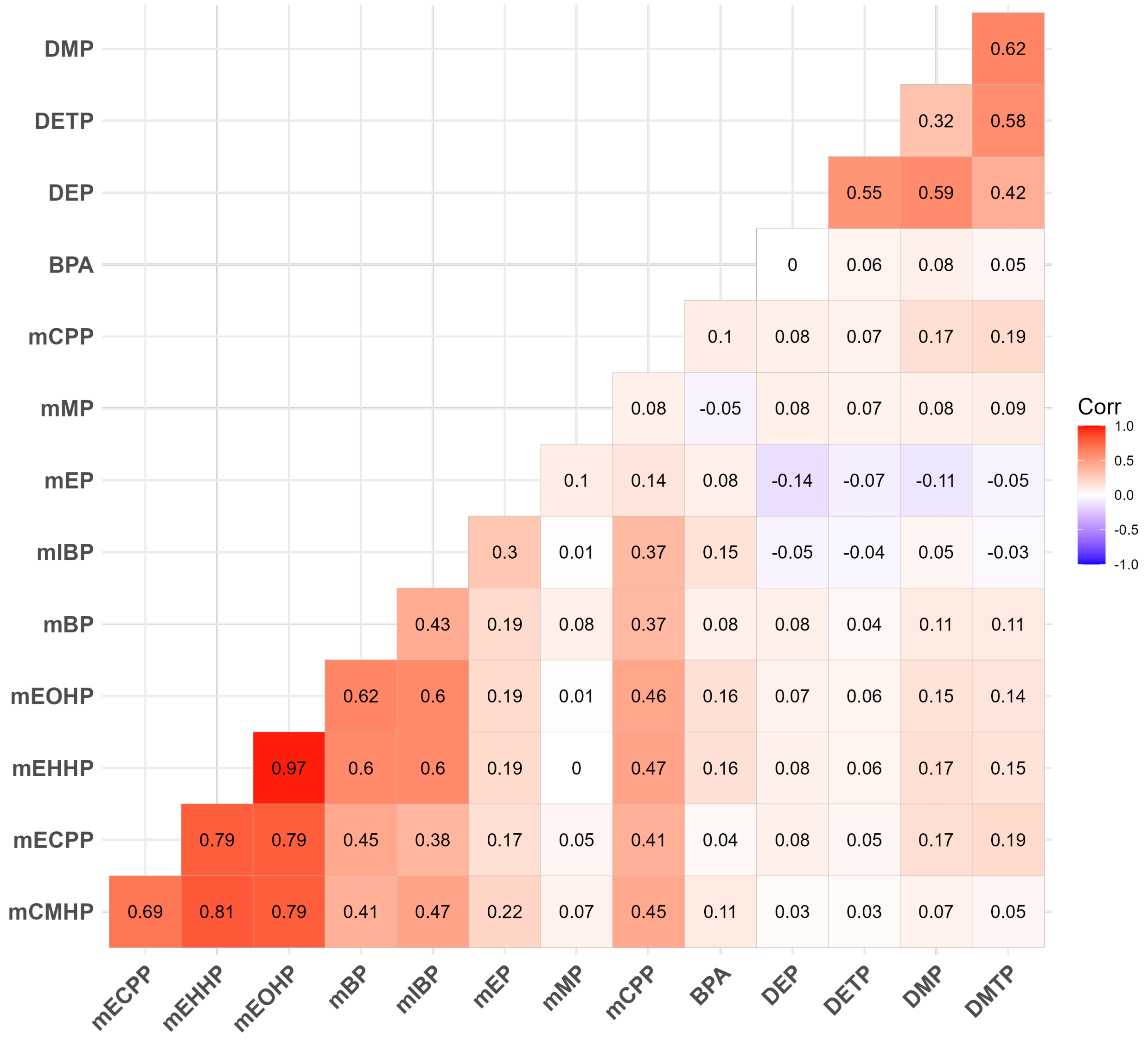

Figure S 5. Correlation matrix for prenatal and childhood exposure biomarker concentrations

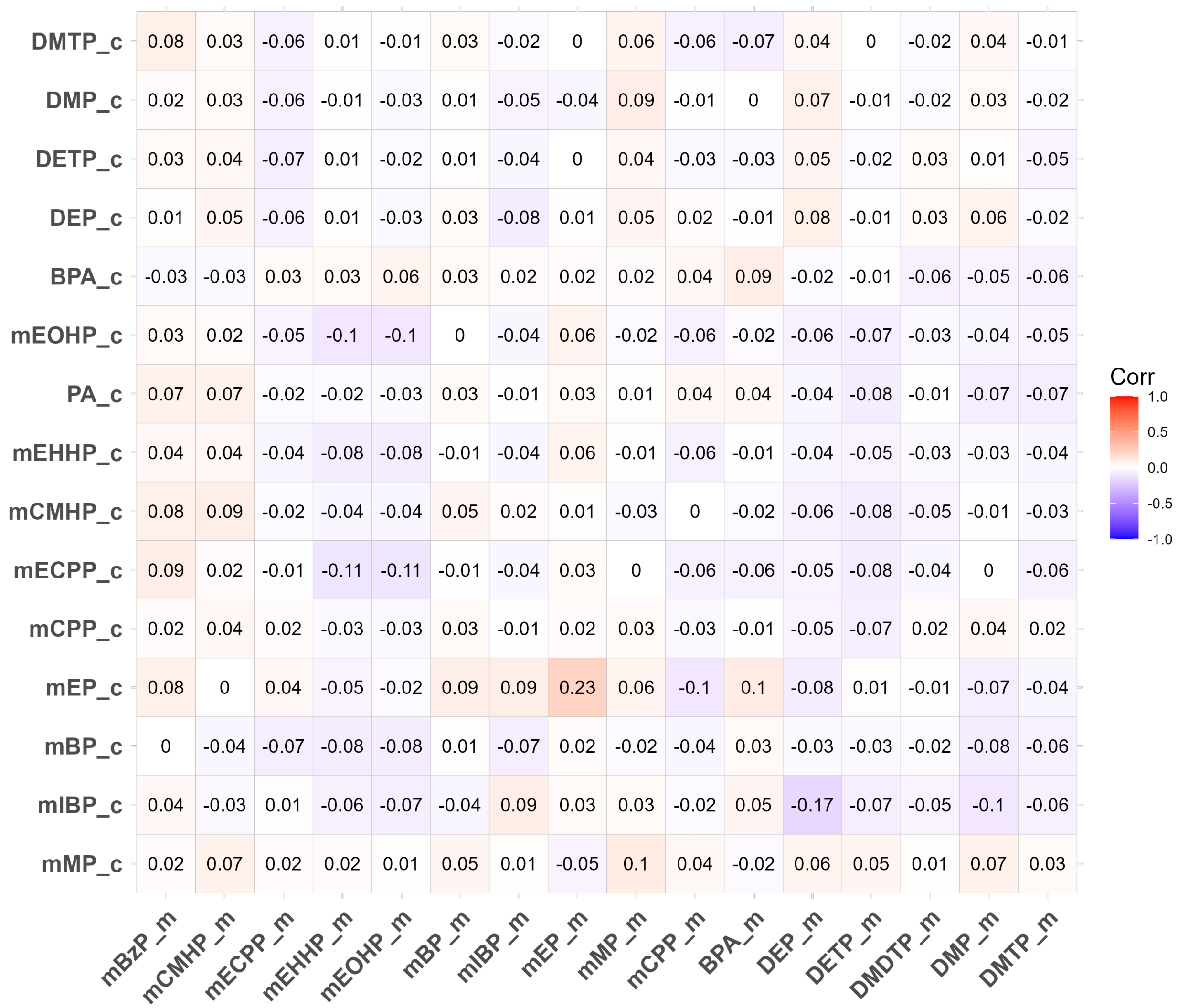

Figure S 6. Univariate exposure-response functions and 95% credible intervals for each biomarker with the other biomarkers fixed at the median (prenatal exposure)

A) Verbal comprehension

Total

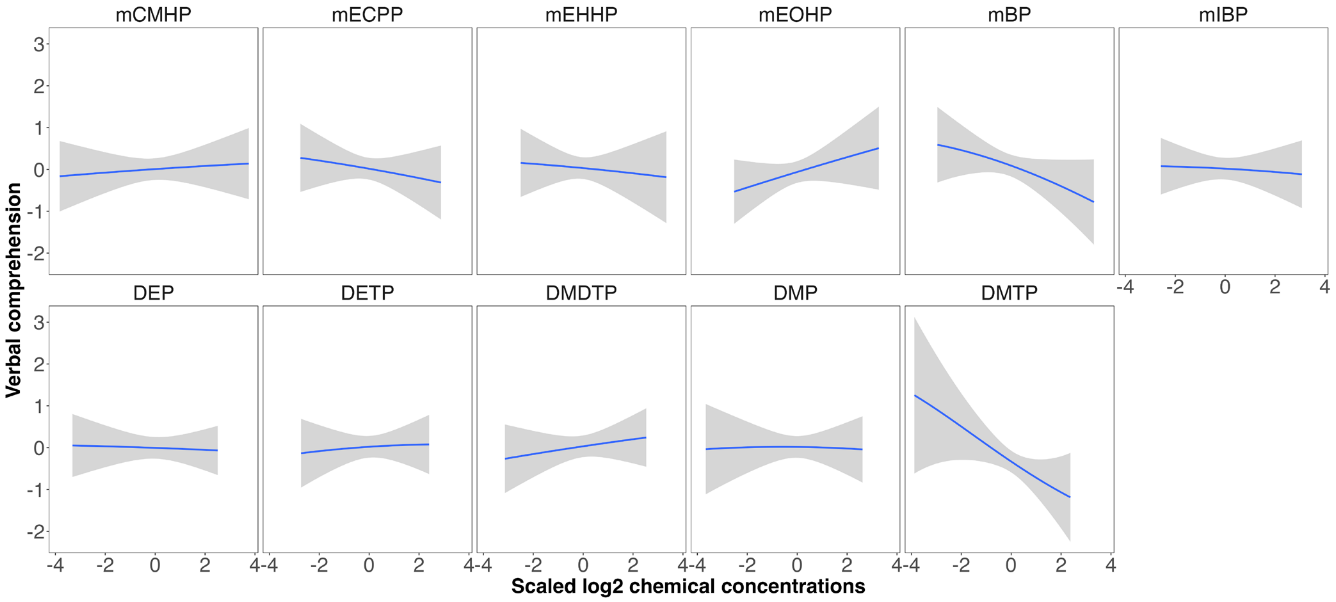

Girls

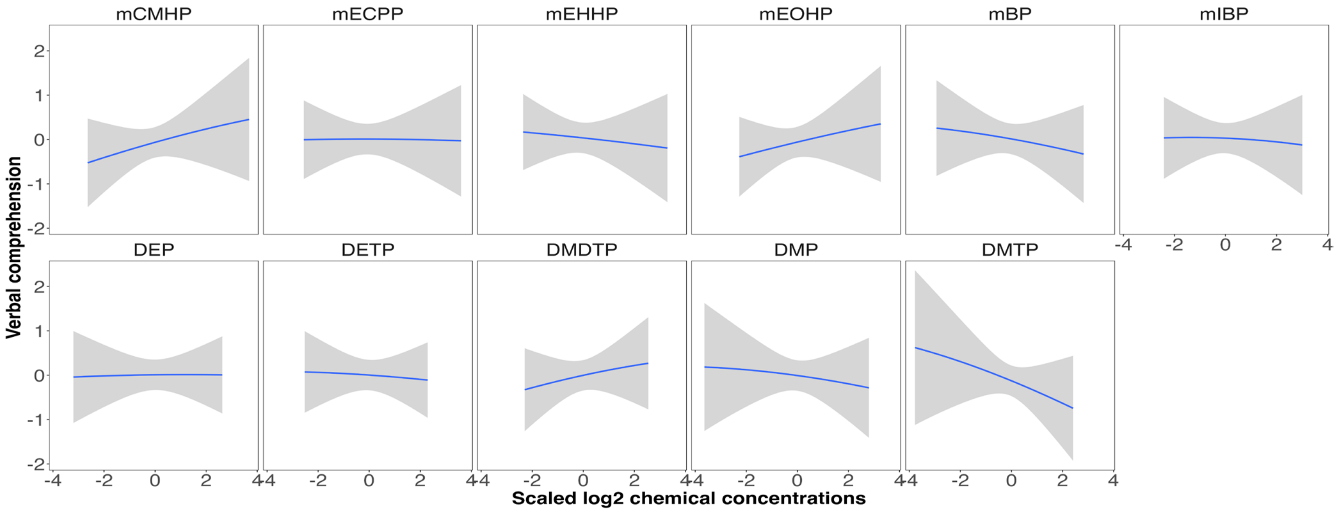

Boys

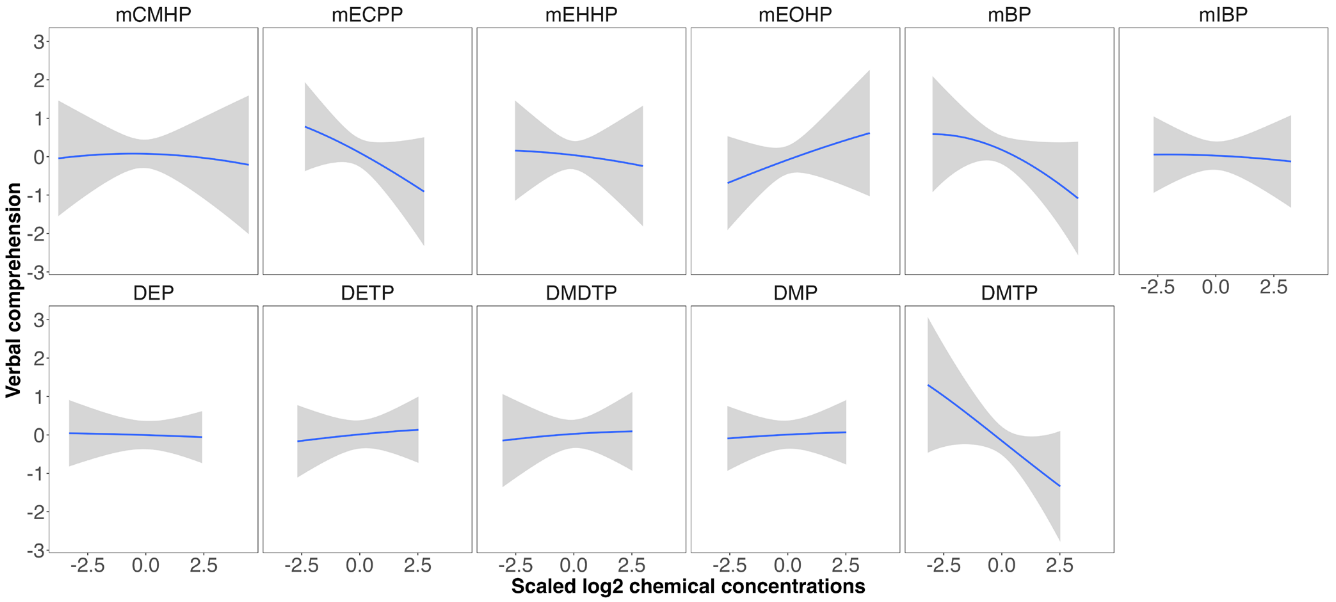

B) Matrix reasoning

Total

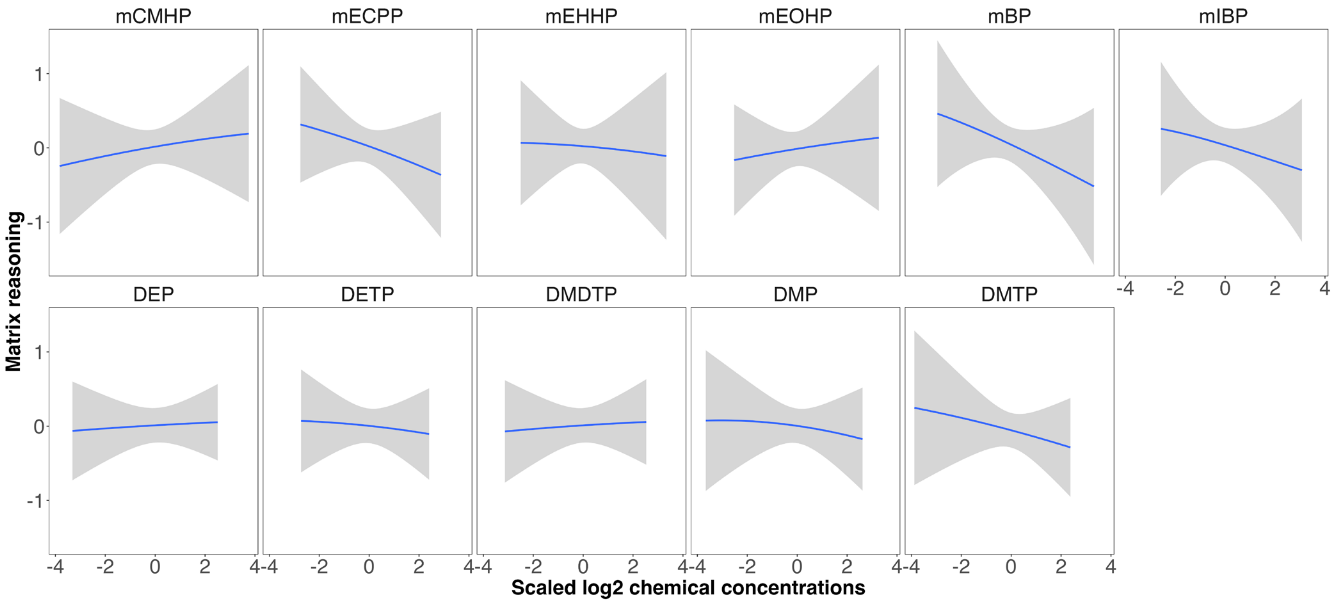

Girls

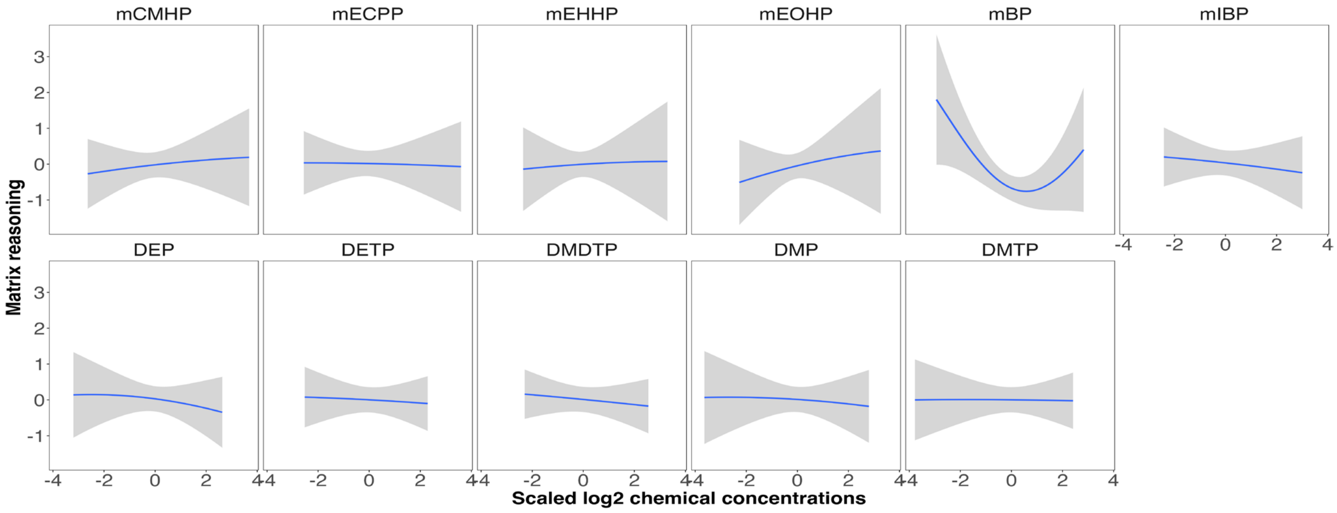

Boys

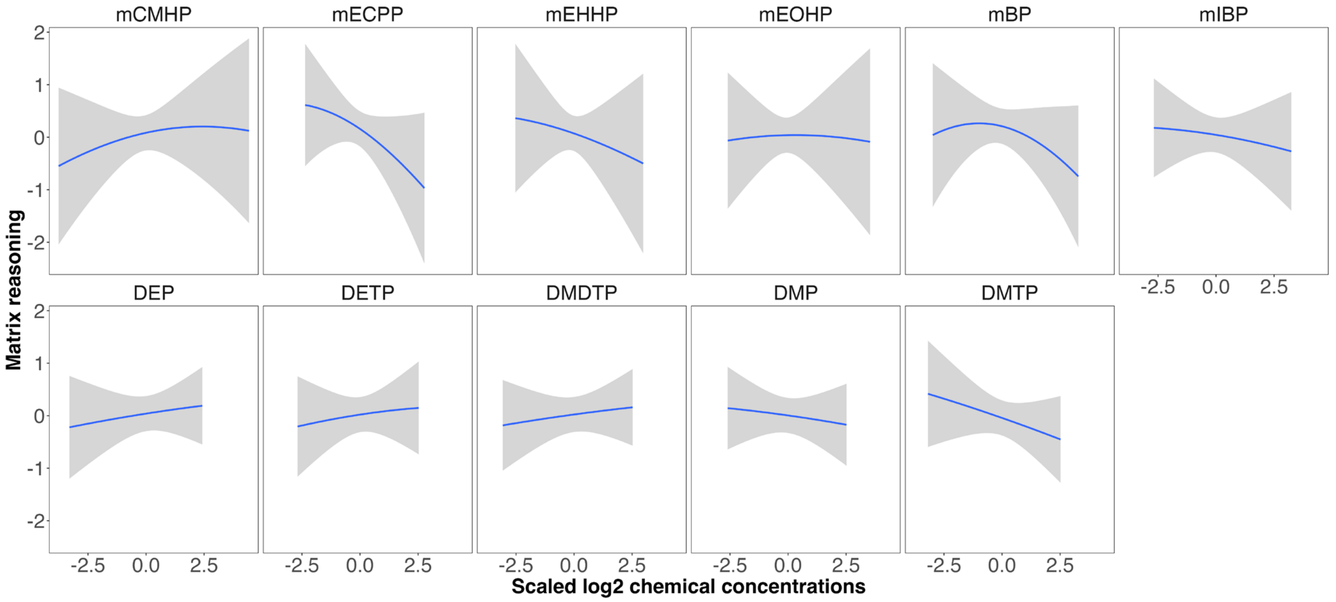

C) Digit span

Total

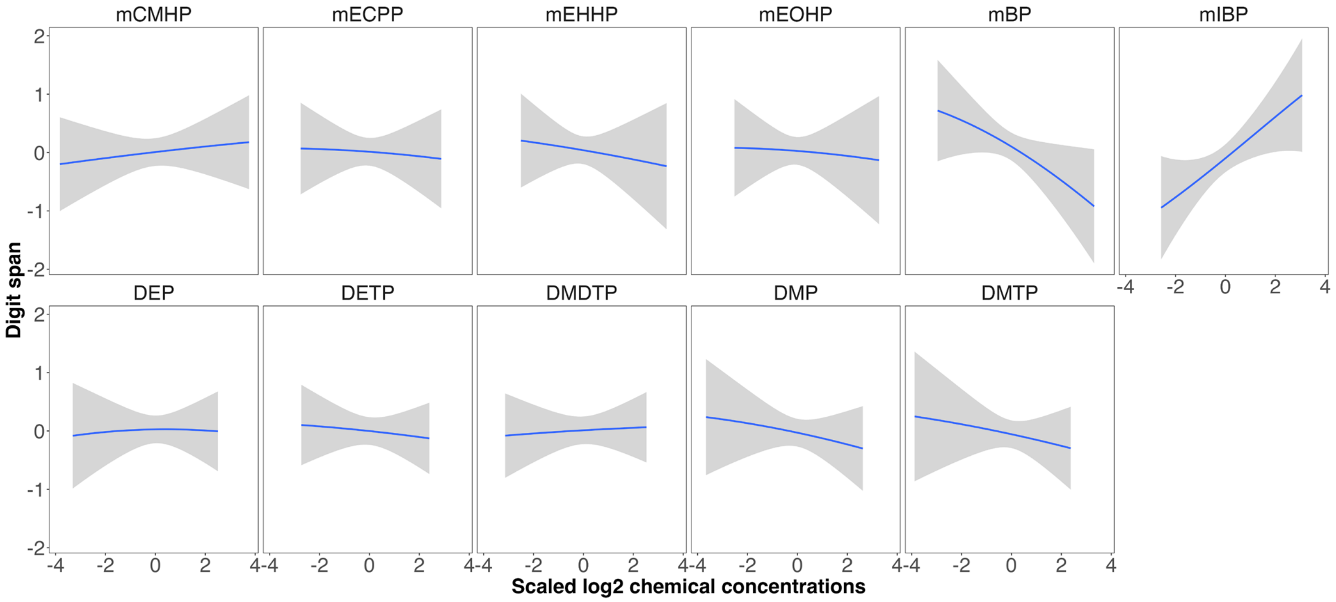

Girls

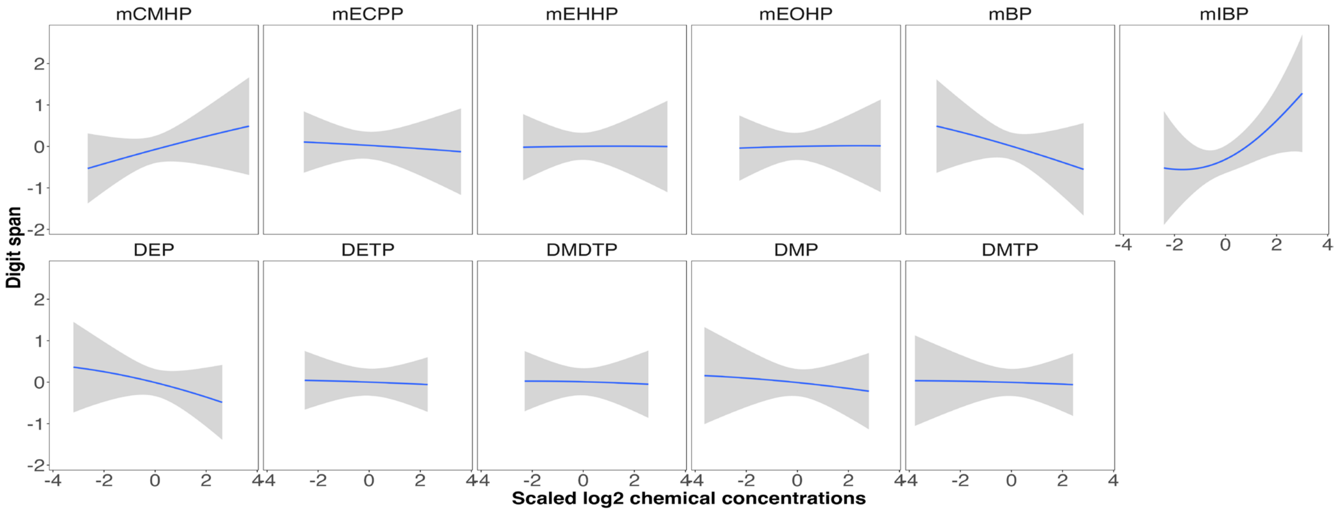

Boys

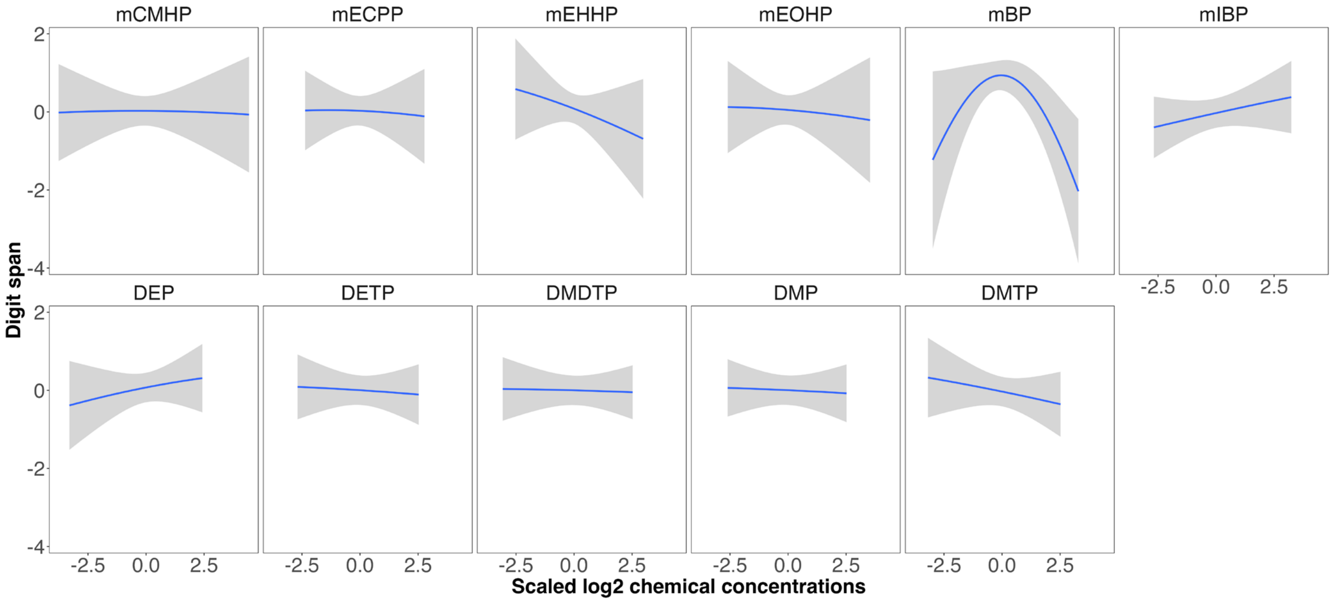

D) Coding

Total

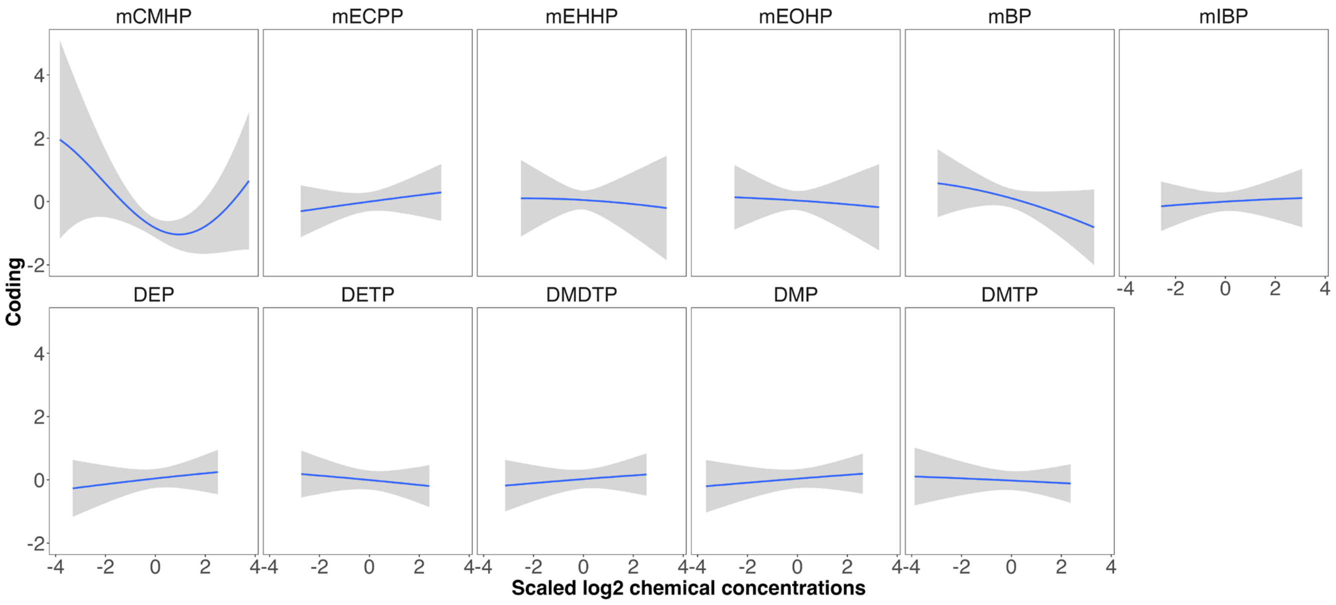

Girls

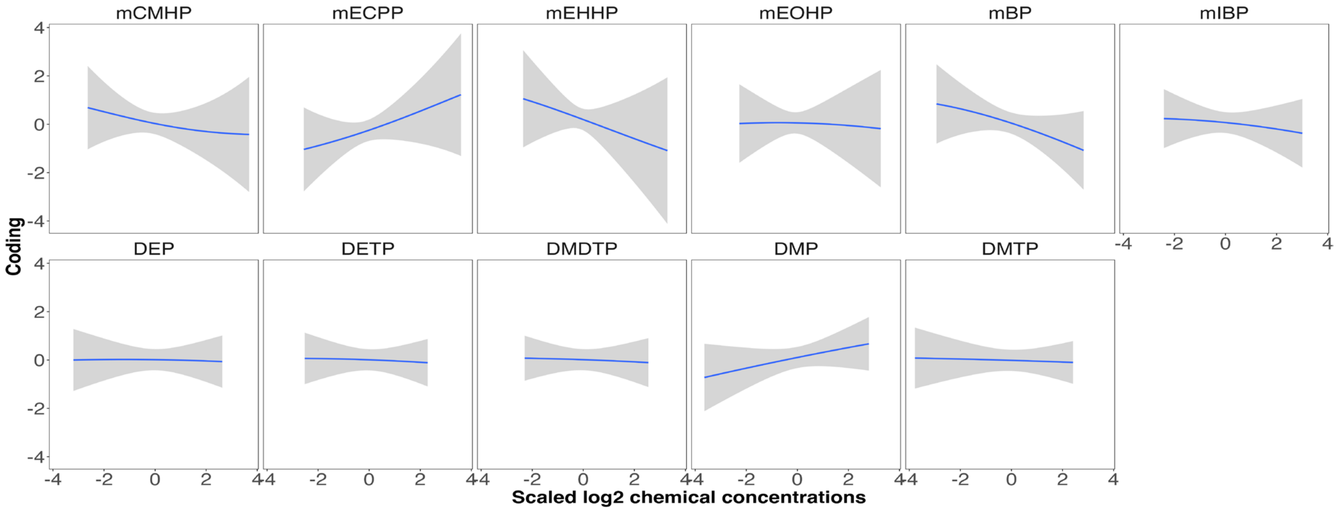

Boys

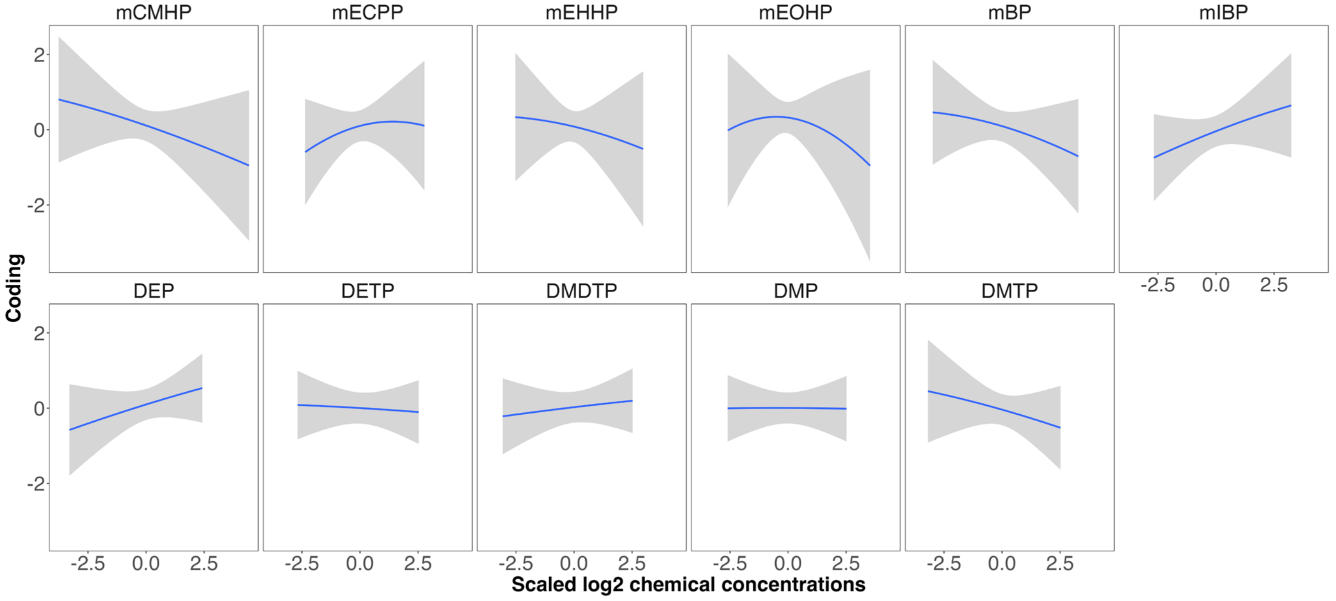

Figure S 7. Single biomarker of prenatal EDC mixture associations with cognitive performance scores in adolescence as estimated by BKMR

A) Verbal comprehension

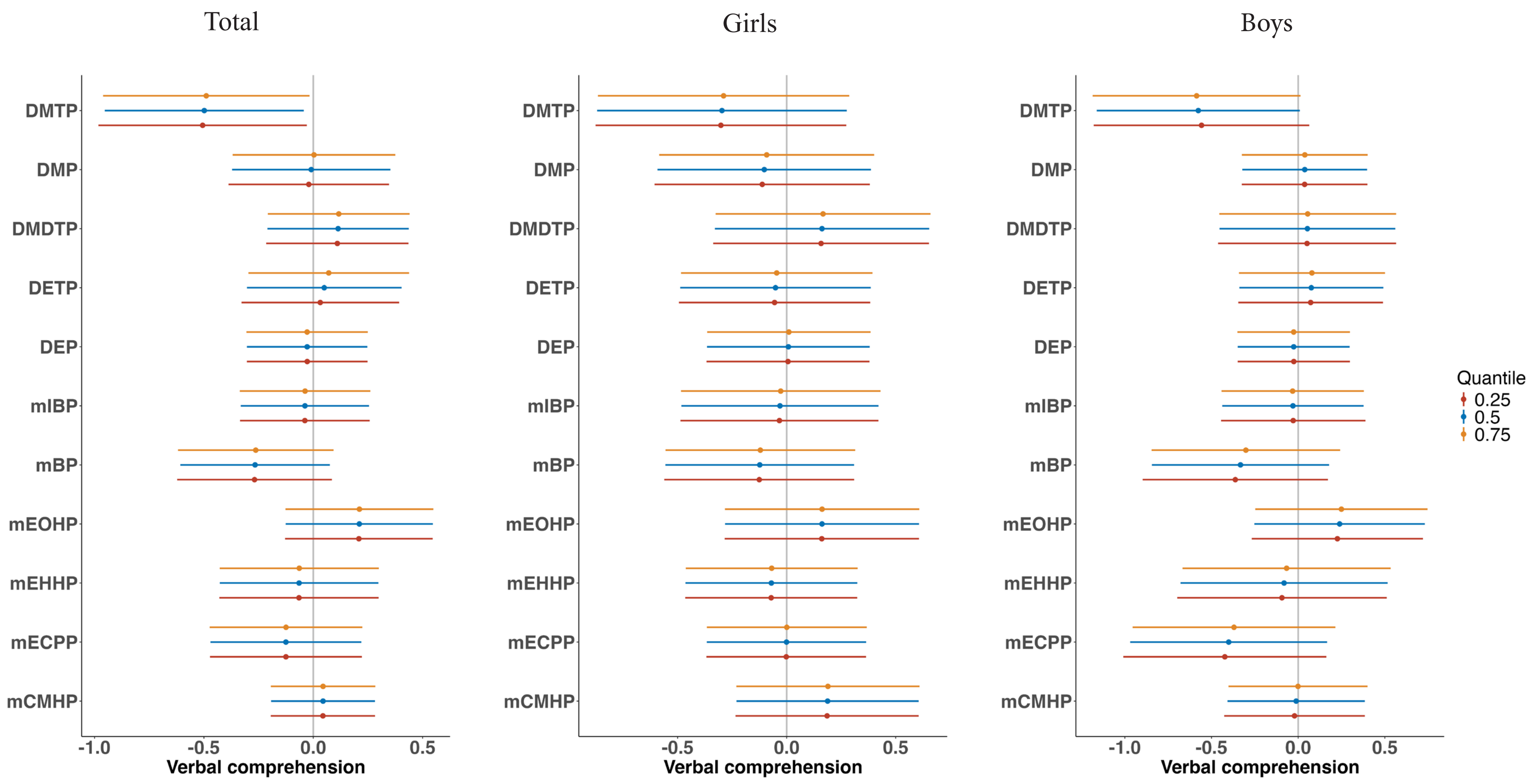

B) Matrix reasoning

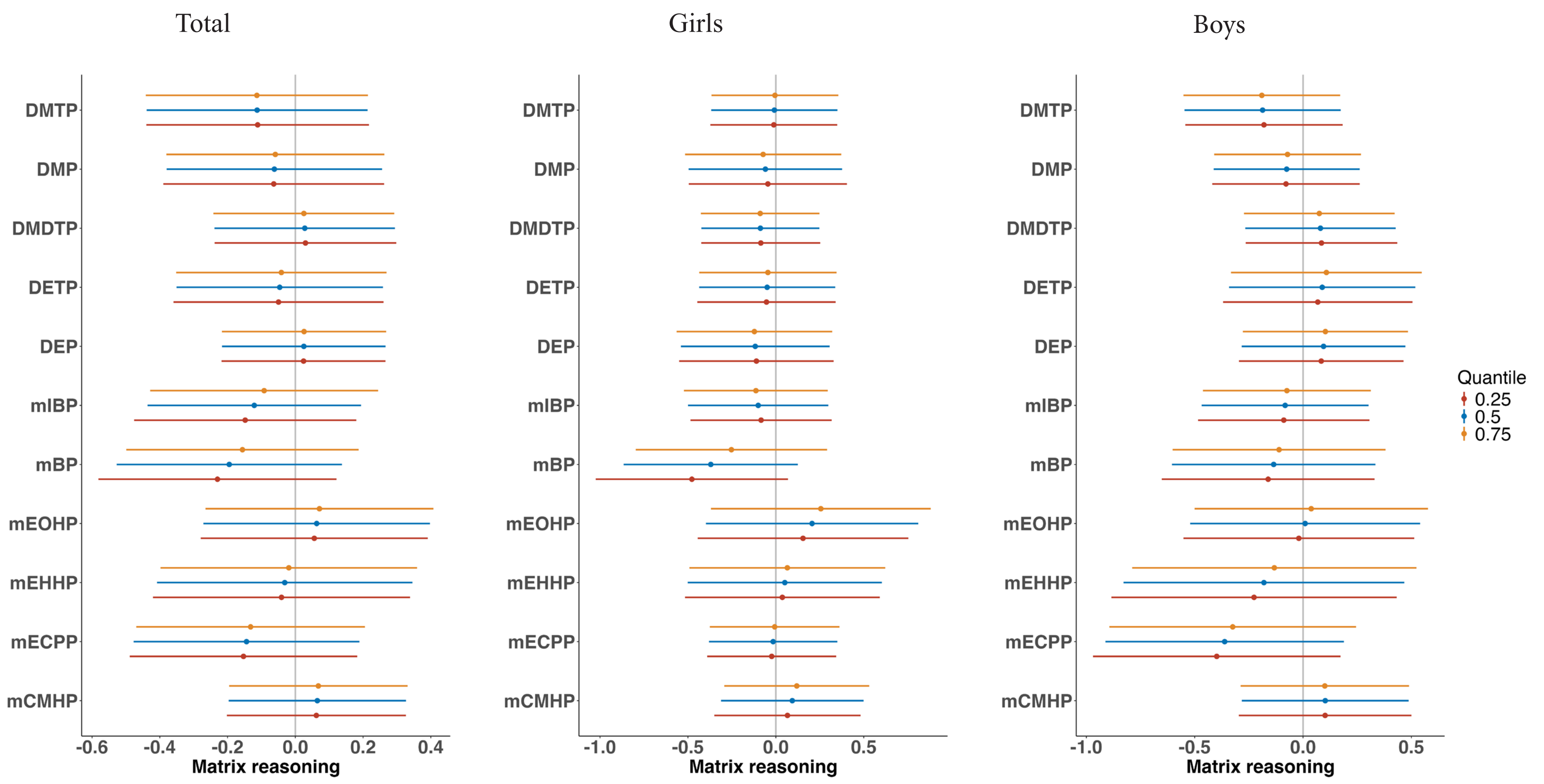

C) Digit span

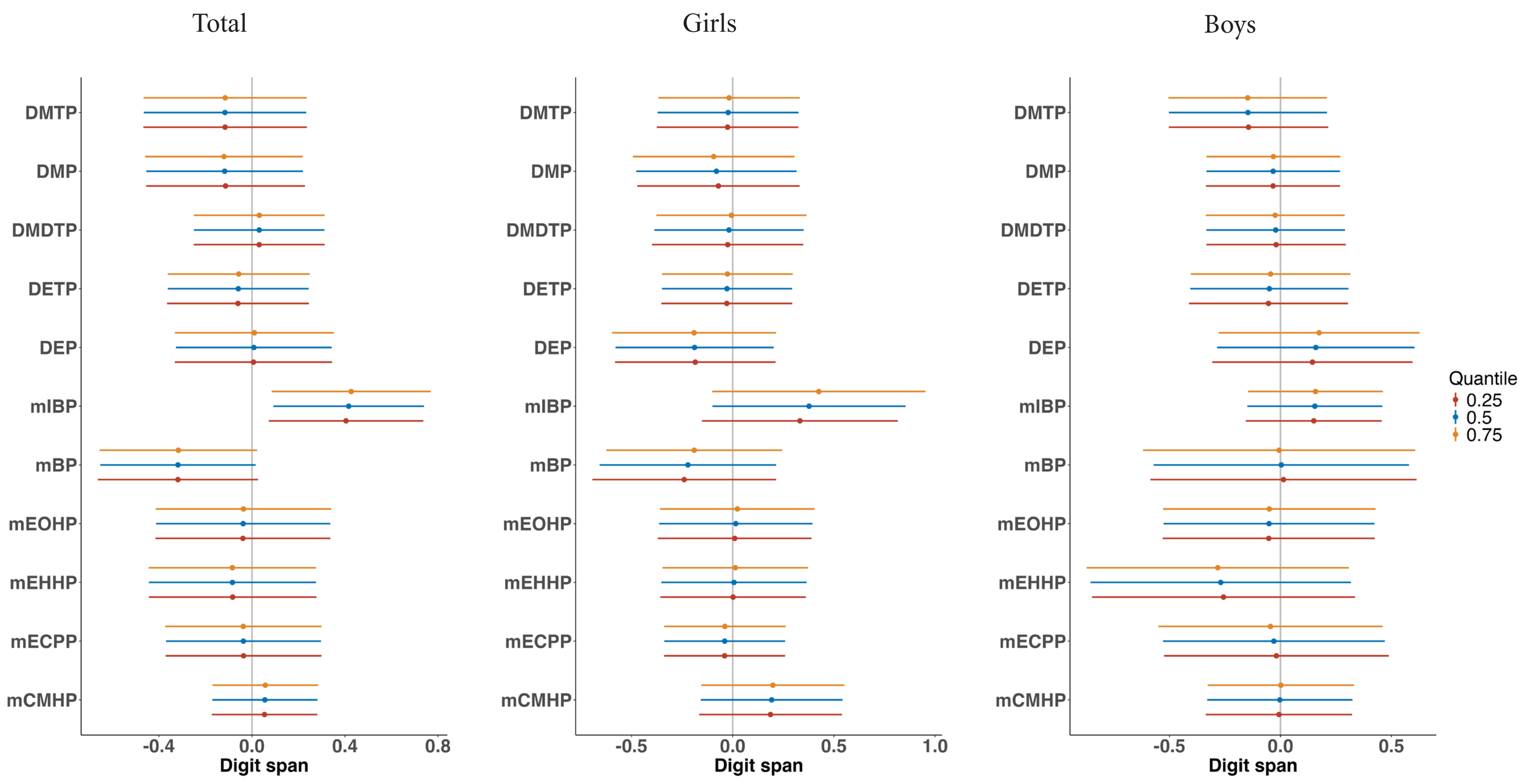

Total
Girls
Boys
DMTP
DMP
DMDTP
DETP
DEP
mIBP
mBP
mEOHP
mEHHP
mECPP
mCMHP
-0.4
0.0
0.4
0.8
Digit span
-0.5
0.0
0.5
1.0
Digit span
-0.5
0.0
0.5
Digit span
Quantile
0.25
0.5
0.75

D) Coding

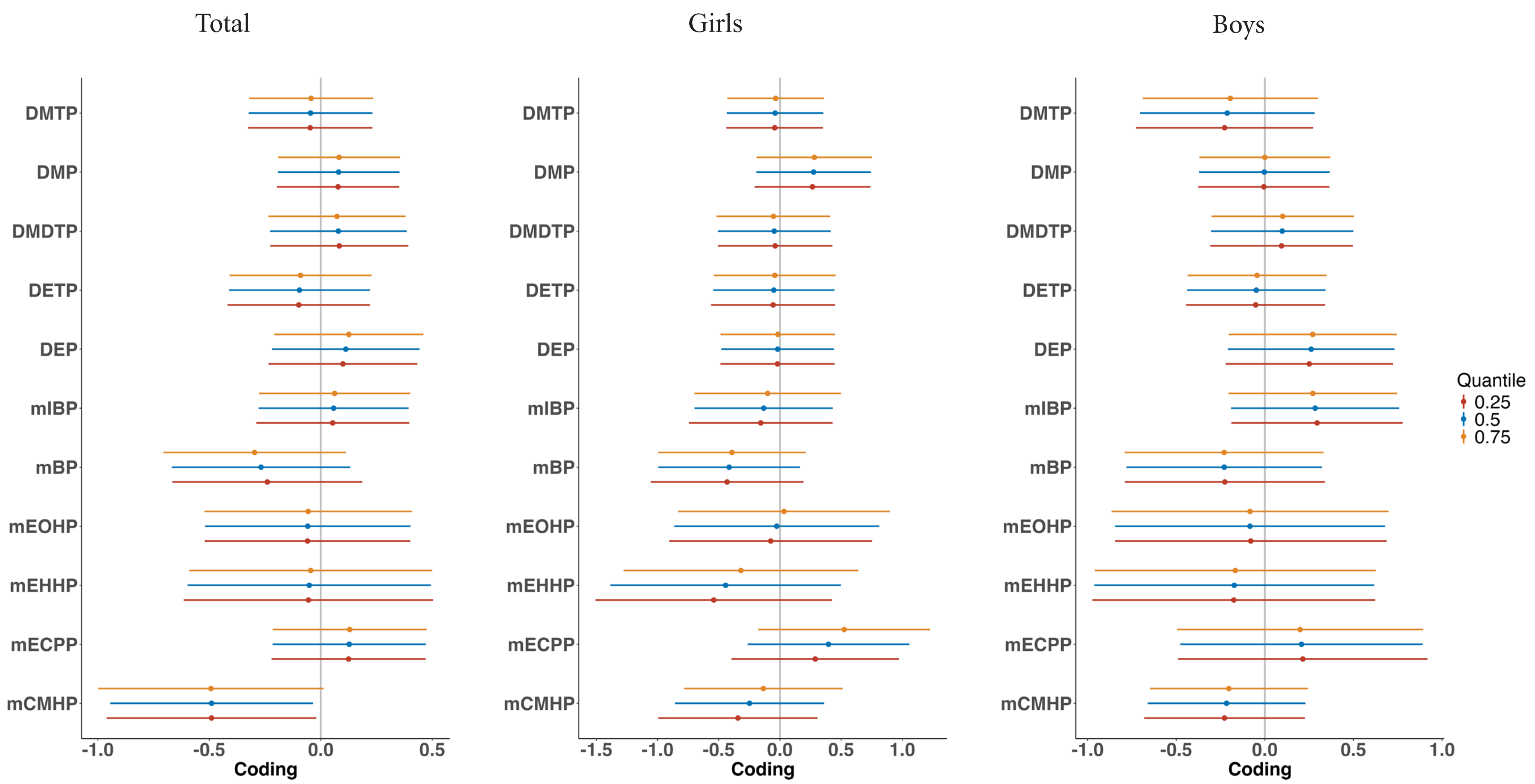

Estimates represent the effect of an IQR change in a single metabolite on cognitive performance scores when all other metabolites are fixed at their 25th, 50th, or 75th percentiles separately.

Figure S 8. Univariate exposure-response functions and 95% credible intervals for each biomarker with the other biomarkers fixed at the median (childhood exposure)

A) Verbal comprehension

Total

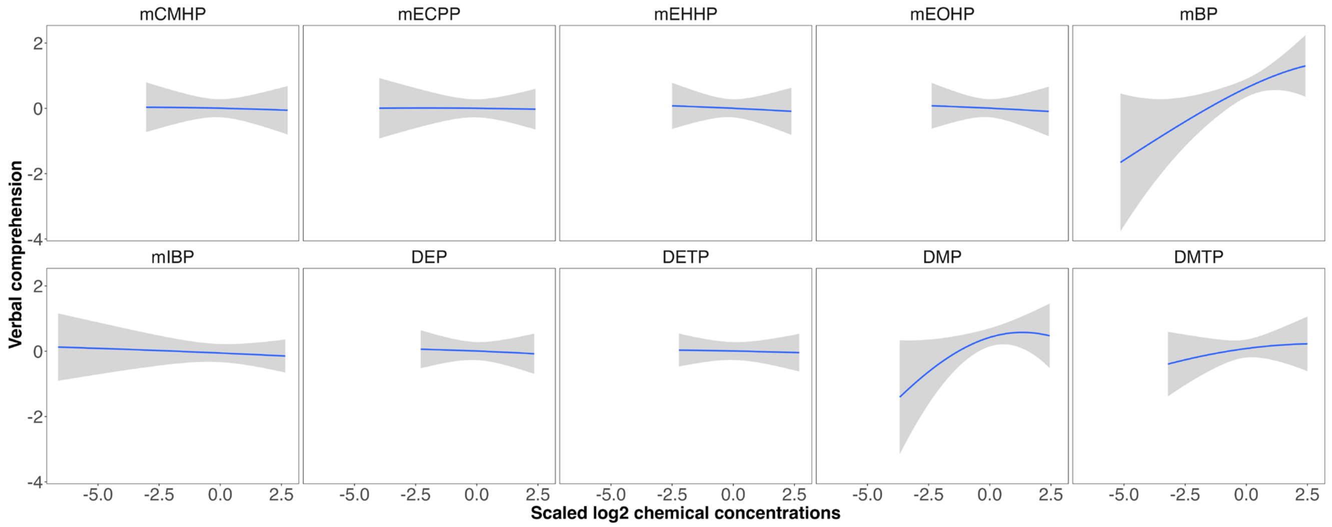

Girls

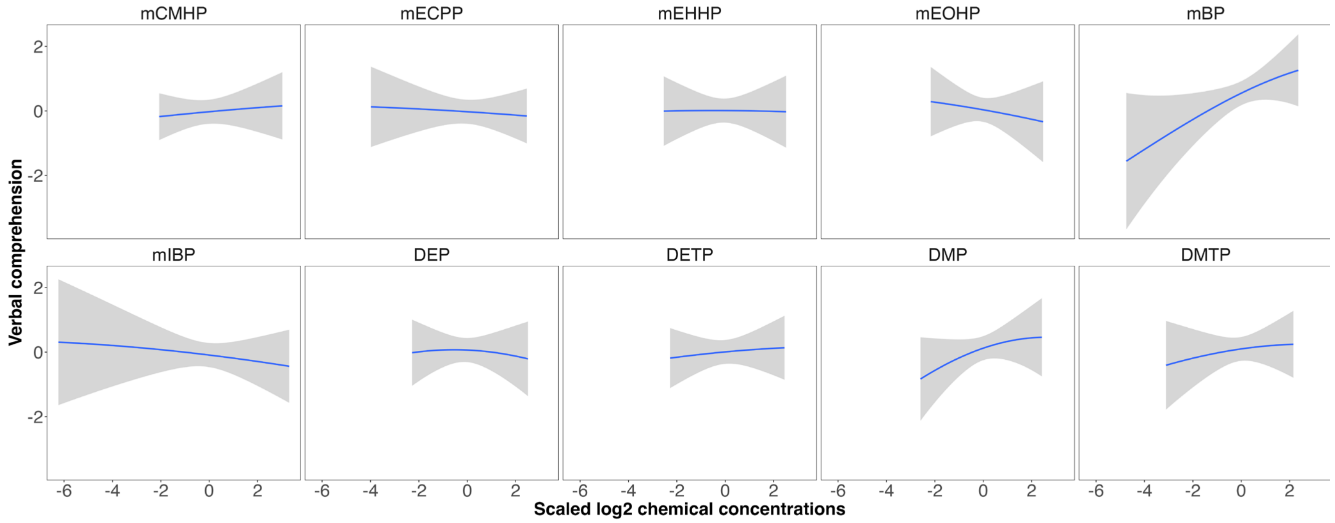

Boys

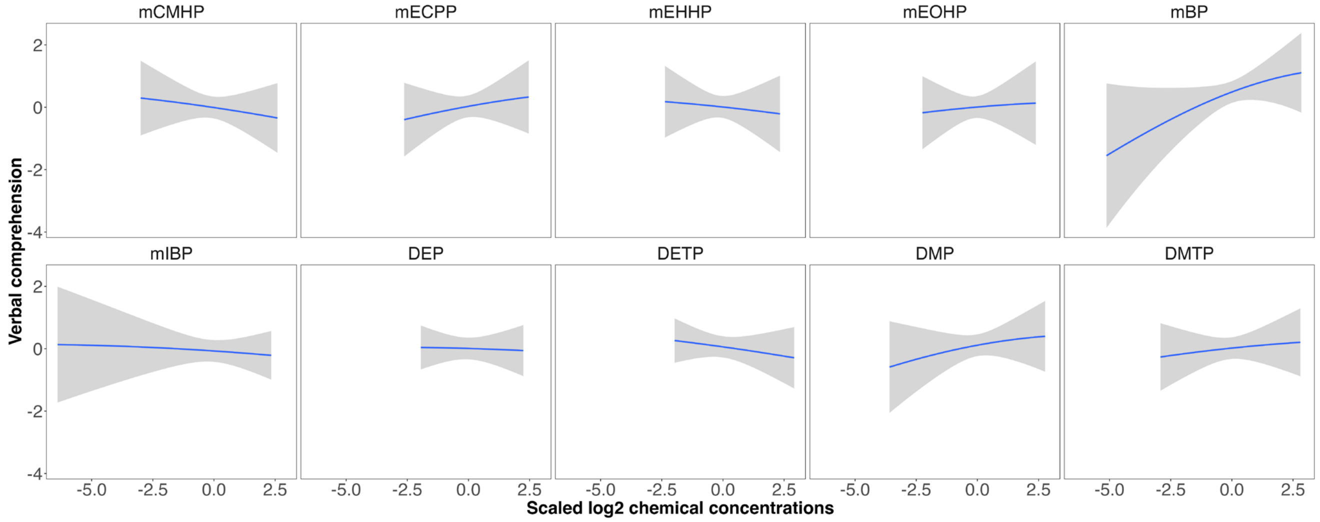

B) Matrix reasoning

Total

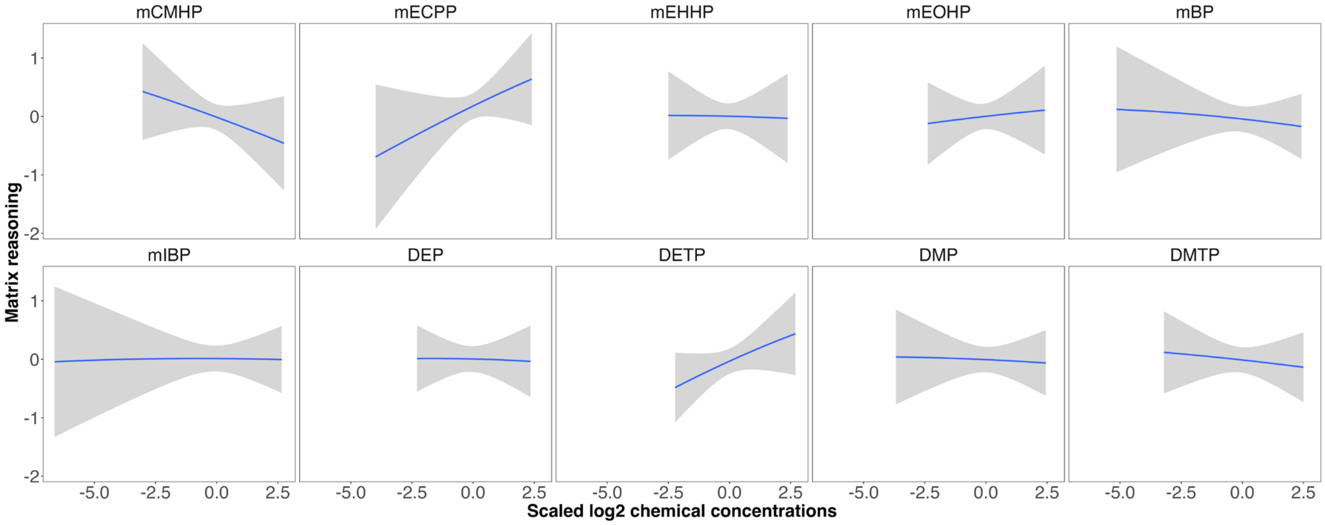

Girls

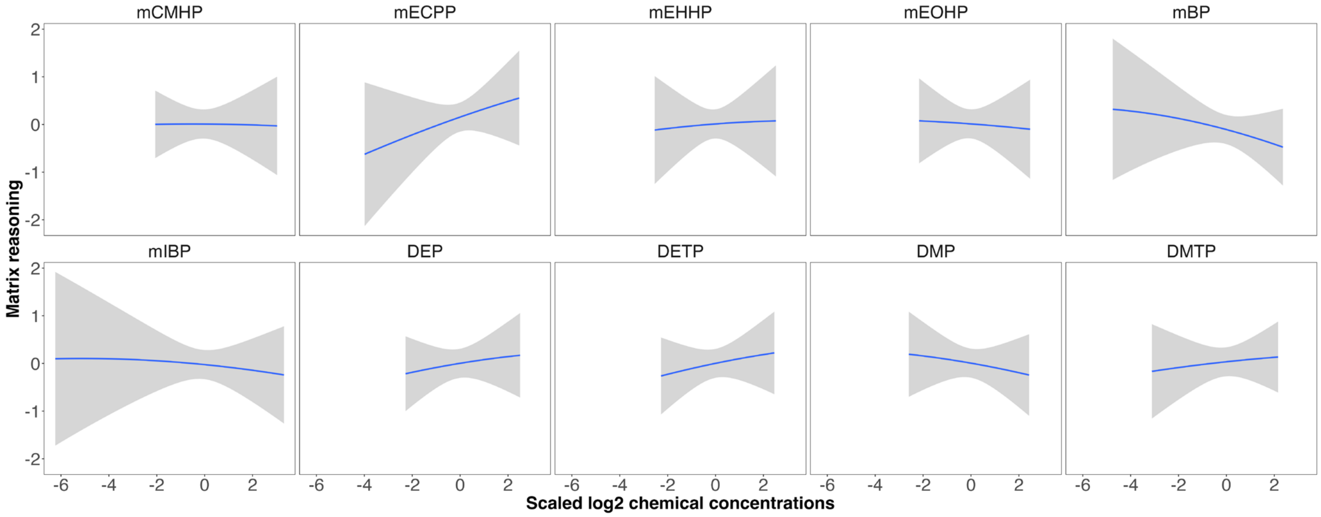

Boys

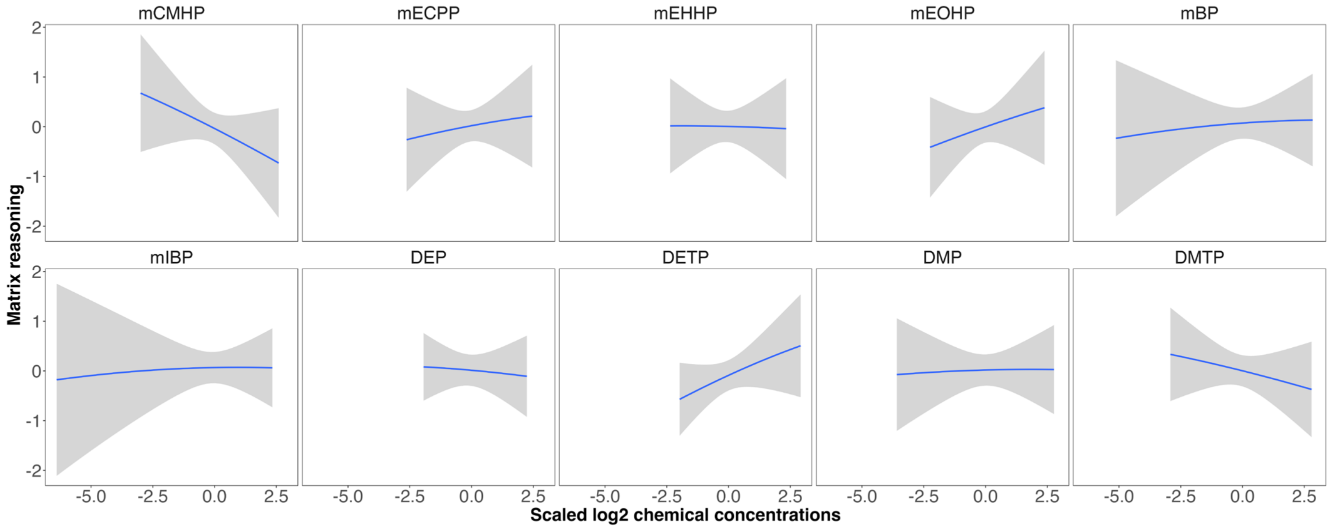

C) Digit span

Total

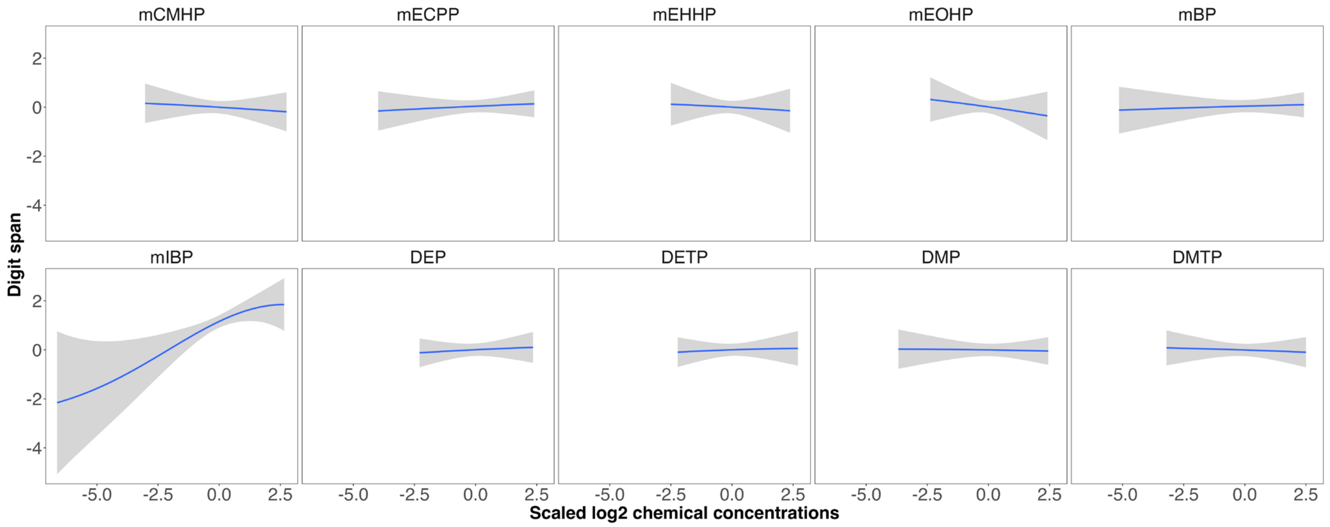

Girls

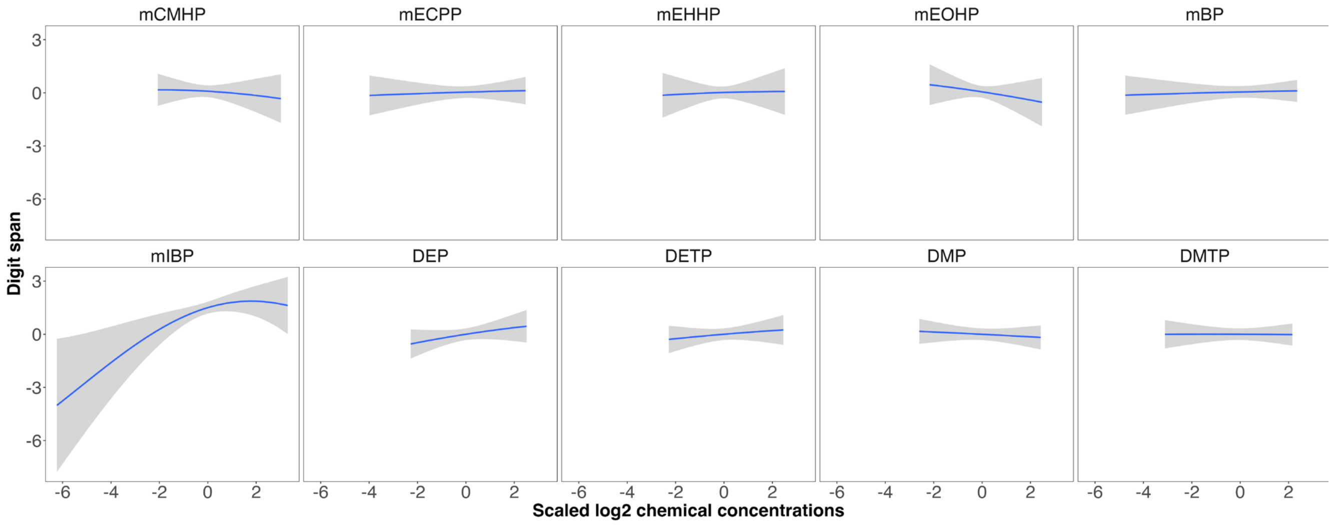

Boys

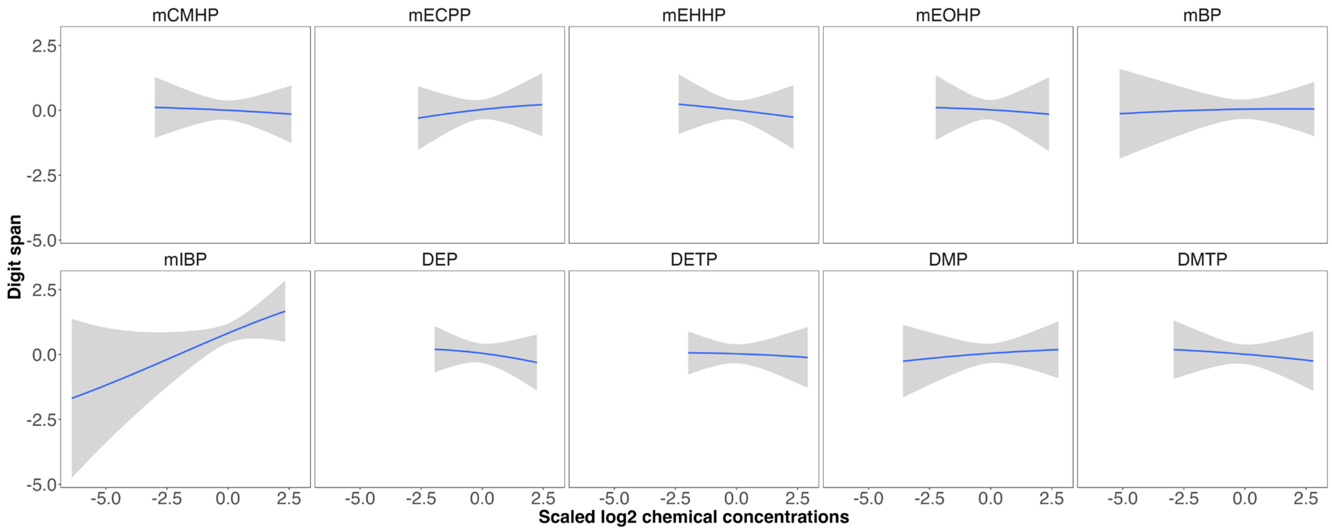

D) Coding

Total

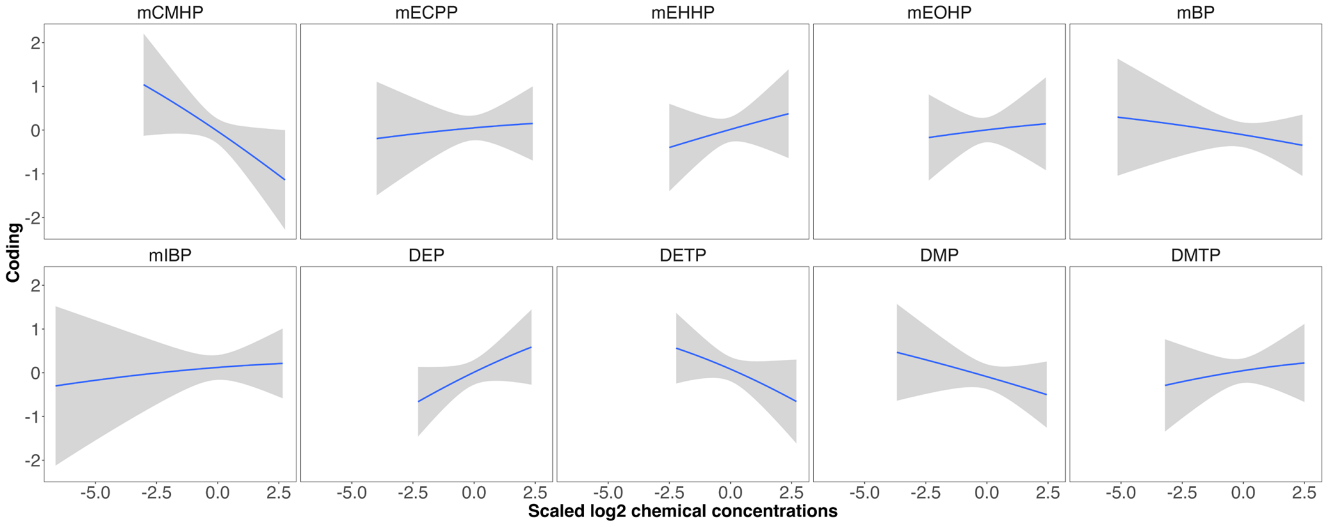

Girls

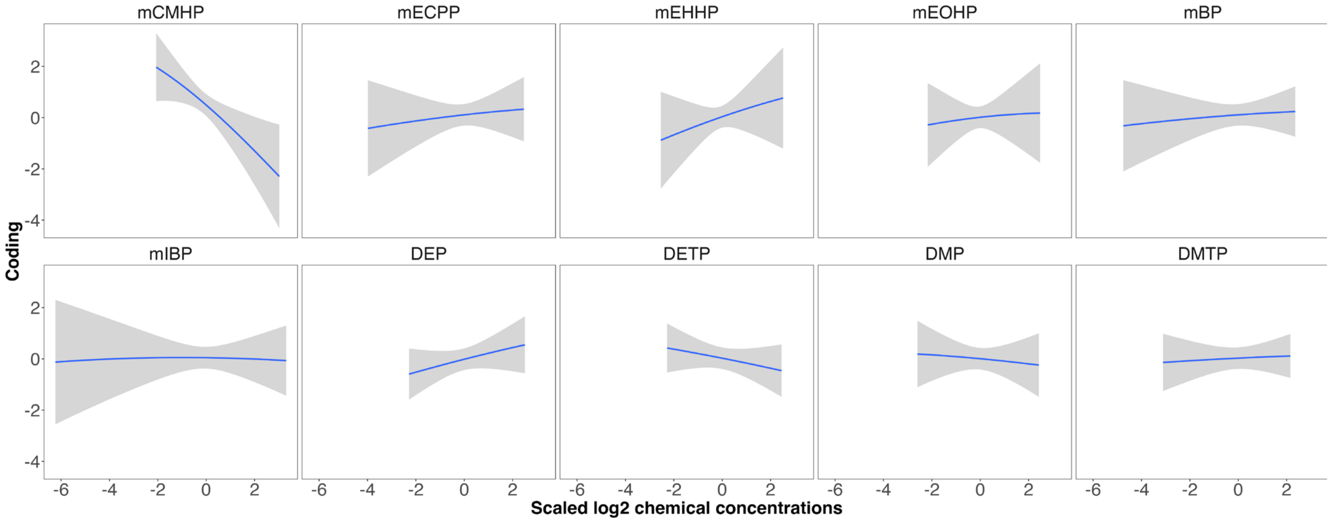

Boys

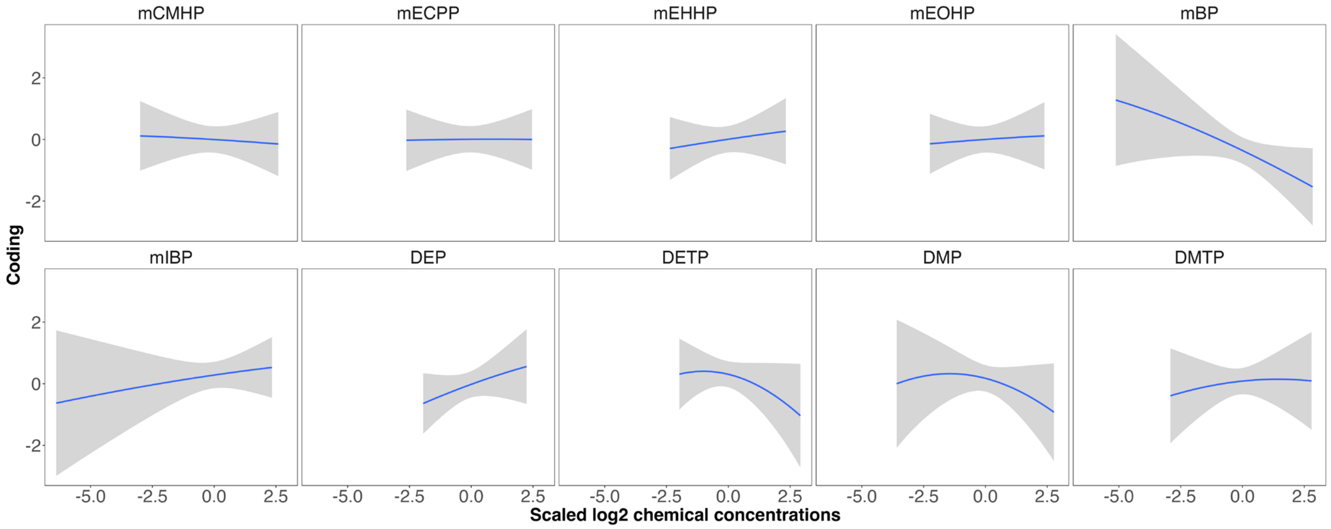

Figure S 9. Single biomarker of childhood EDC mixture associations with cognitive performance scores in adolescence as estimated by BKMR

A) Verbal comprehension

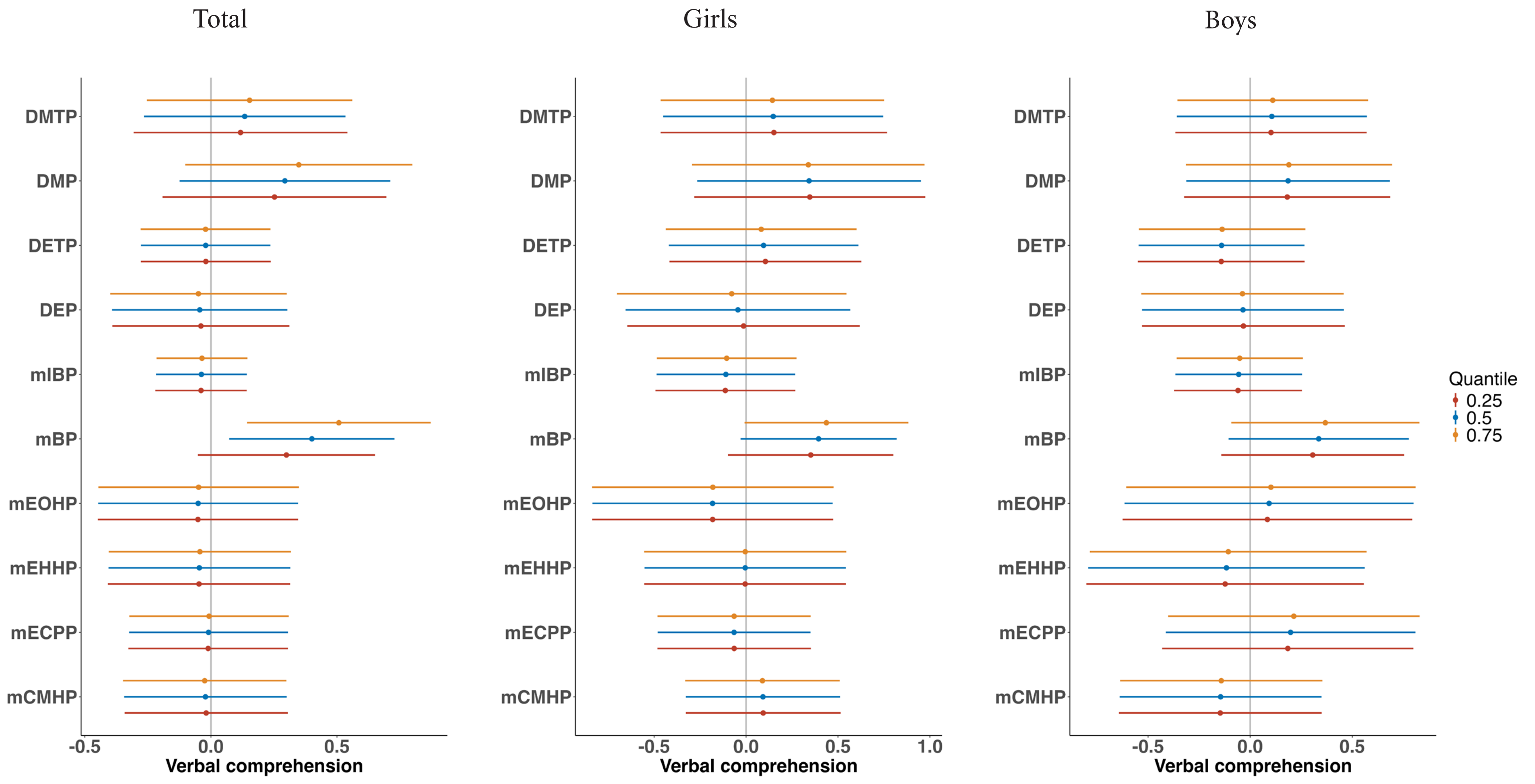

B) Matrix reasoning

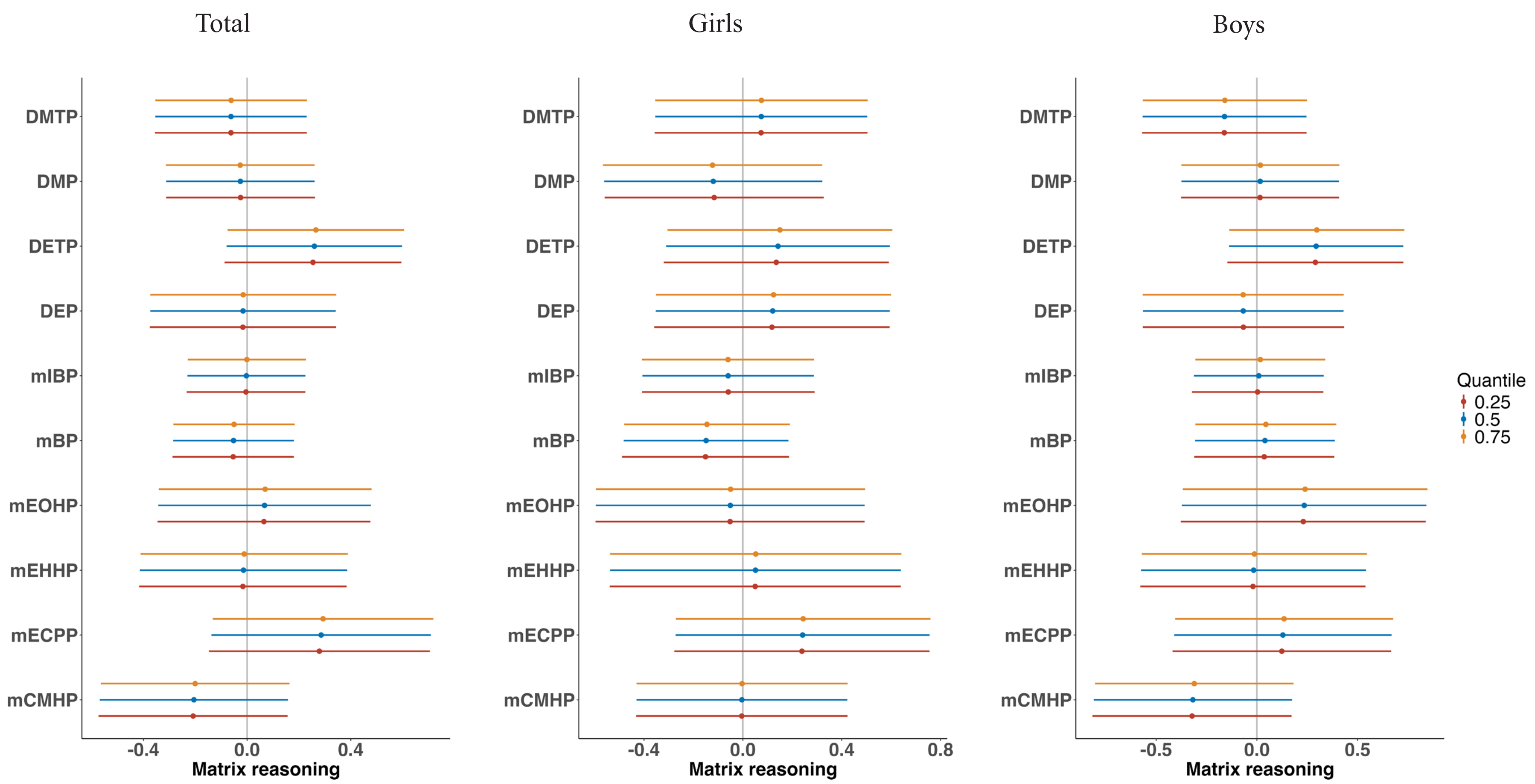

C) Digit span

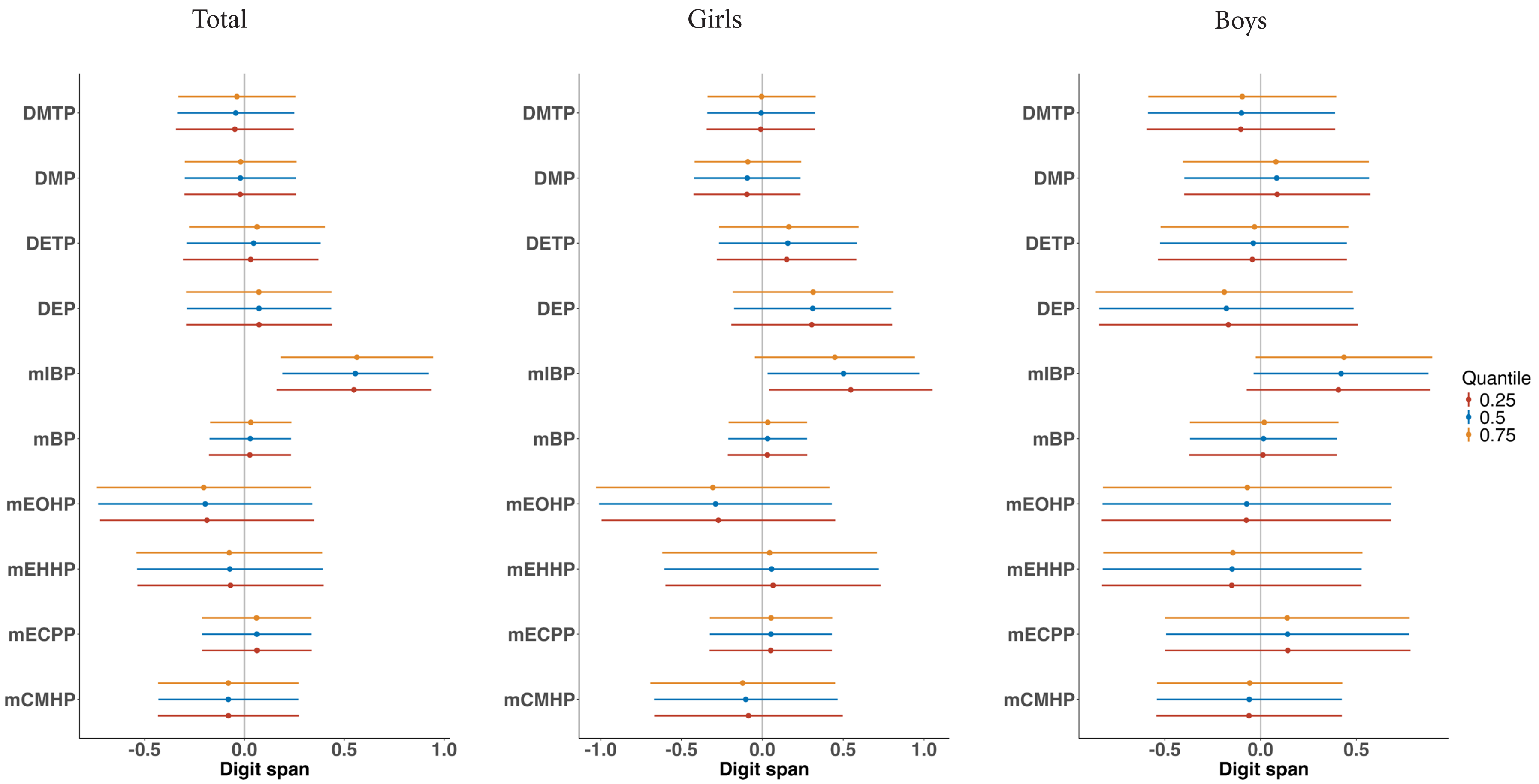

Total
Girls
Boys
DMTP
DMP
DETP
DEP
mIBP
mBP
mEOHP
mEHHP
mECPP
mCMHP
-0.5
0.0
0.5
1.0
-1.0
Digit span
Quantile
0.25
0.5
0.75

D) Coding

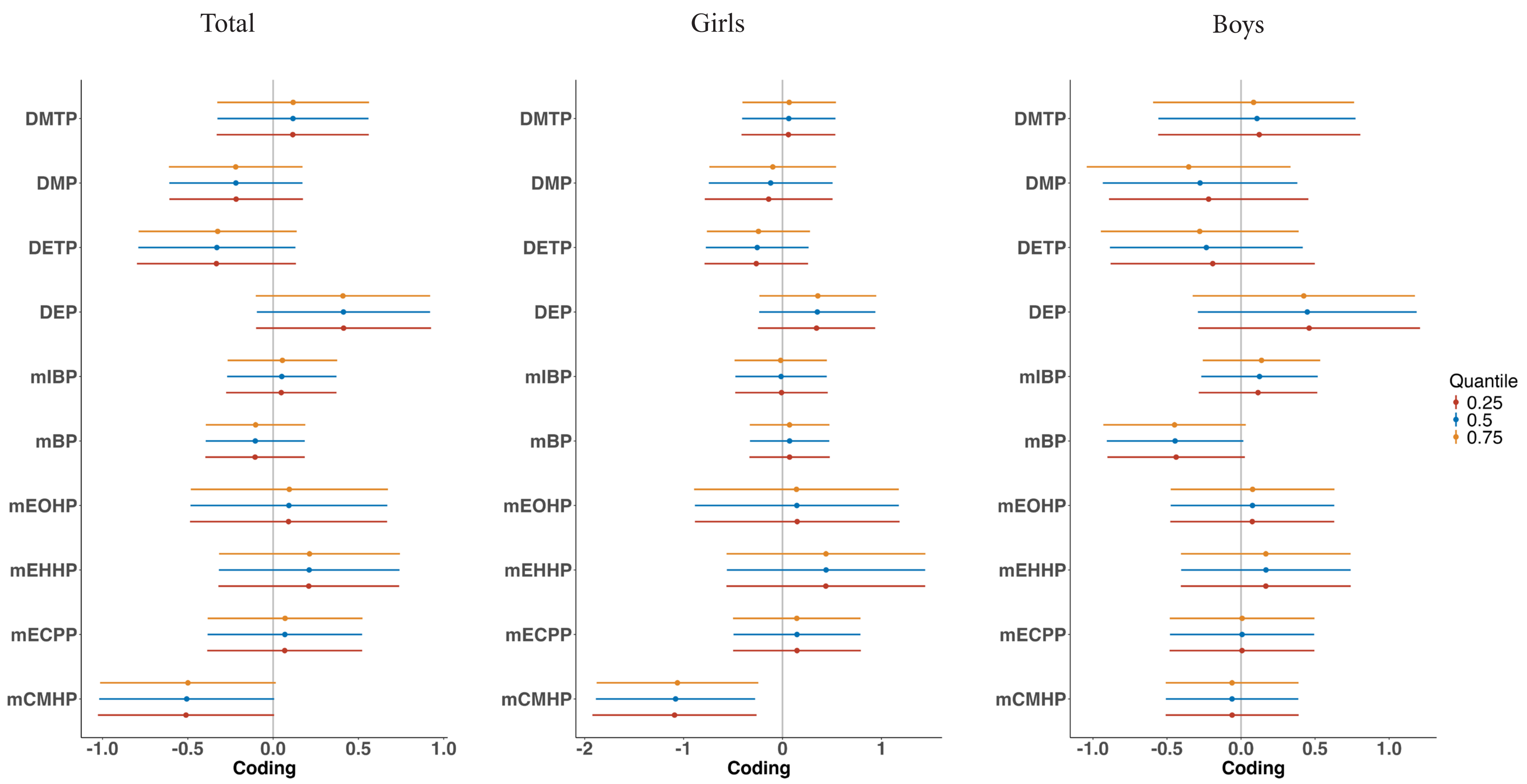

Estimates represent the effect of an IQR change in a single metabolite on cognitive performance scores when all other metabolites are fixed at their 25th, 50th, or 75th percentiles separately.

Figure S10. Overall effects of exposure to the mixture of phthalates, bisphenols and organophosphate pesticides in childhood on cognitive performance scores in adolescence; model additionally adjusted for prenatal phthalic acid, bispenol A and non-specific dialkylphosphate metabolites

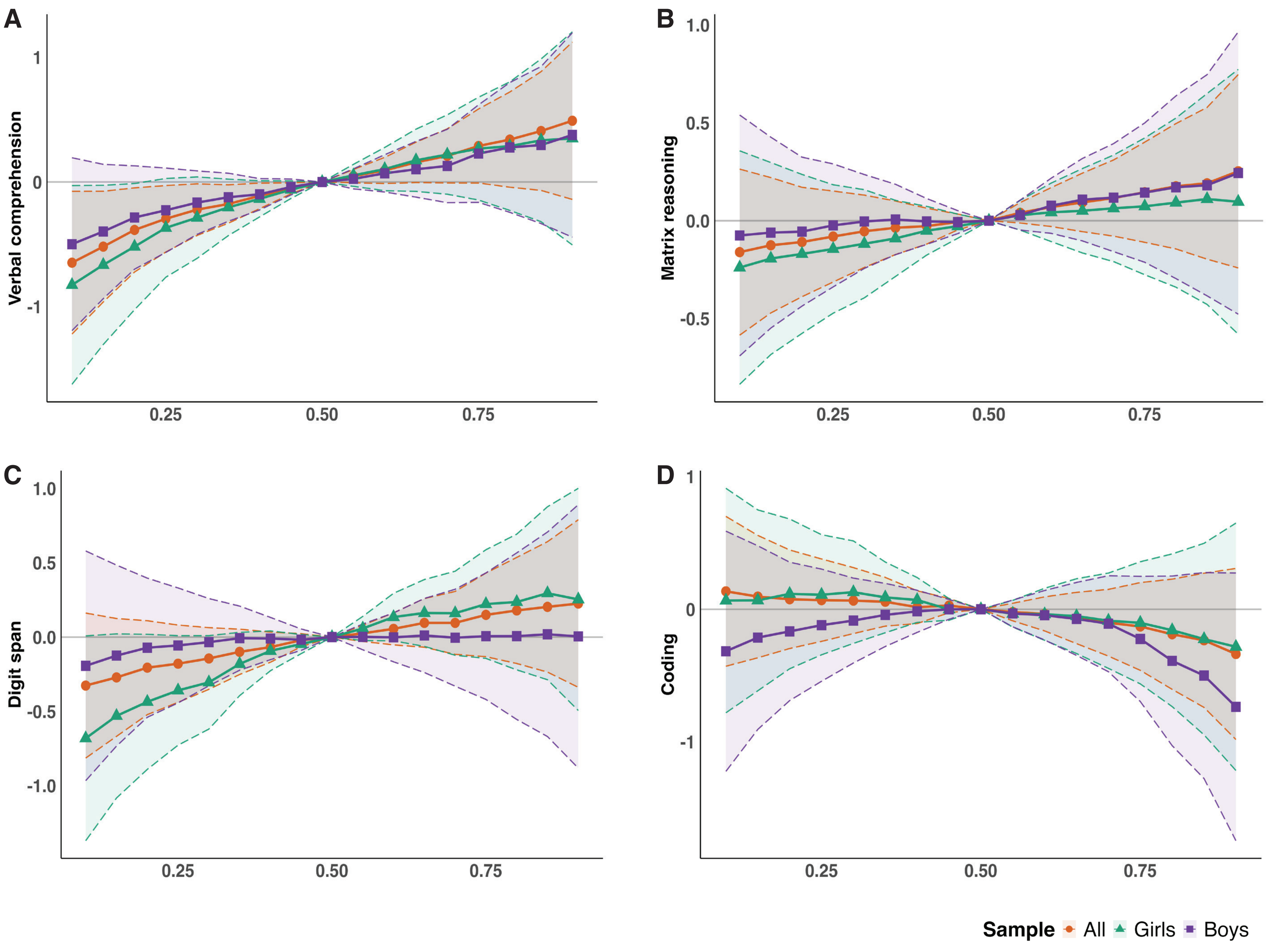

Supplement: Supplementary file 1 [file es5c18986_si_001.pdf]
